# Investigation of Evolutionary History and Origin of the Tre1 Family Suggests a Role in Regulating Hemocytes Cells Infiltration of the Blood–Brain Barrier

**DOI:** 10.3390/insects12100882

**Published:** 2021-09-29

**Authors:** Norwin Kubick, Pavel Klimovich, Irmina Bieńkowska, Piotr Poznanski, Marzena Łazarczyk, Mariusz Sacharczuk, Michel-Edwar Mickael

**Affiliations:** 1 Department of Biochemistry and Molecular Cell Biology (IBMZ), University Medical Center Hamburg-Eppendorf, Martinistraße 52, 20246 Hamburg, Germany; n.kubick@uke.de; 2 Department of Immunology, PM Forskningscentreum, 17854 Ekerö, Sweden; klimovichpavlusha@gmail.com; 3 Department of Experimental Genomics, Institute of Animal Biotechnology and Genetics, Polish Academy of Science, Postępu 36A, 05-552 Subcarpathia, Poland; i.bienkowska@igbzpan.pl (I.B.); piotr.poznanski91@gmail.com (P.P.); m.lazarczyk@igbzpan.pl (M.Ł.); m.sacharczuk@igbzpan.pl (M.S.)

**Keywords:** paracellular, transcellular, diapedesis, Tre1

## Abstract

**Simple Summary:**

Understanding the evolutionary association between immune cells and the blood–brain barrier (BBB) is vital to develop therapeutic approaches. In *Drosophila*, glial cells form the BBB that regulates the access of hemocytes to the brain. It is still not known which diapedesis route hemocytes cells follow. In vertebrates, paracellular migration is dependent on PECAM1, while transcellular migration is dependent on the expression of CAV1. The drosophila genome lacks both genes. The Tre1 family (Tre1, *moody*, and Dmel_CG4313) contribute to regulating transepithelial migration in *Drosophila*. However, its evolutionary history is not known. We performed phylogenetic analysis to reconstruct the evolutionary history of the Tre1 family. We found Dmel_CG4313 only in insects. Tre1 exists only in invertebrates and is highly conserved. *moody* evolutionary history is more spread as it appears from Cnidaria up to mammals and is less conserved. The Tre1 family origin seems to be related to opsins. We have identified an SH3 motif in Tre1, *moody*, and Dmel_CG4313. SH3 regulates actin movement in a Rho-dependent manner in PECAM1. Our results suggest that the Tre1 family could be playing an important role in paracellular diapedesis in *Drosophila*. Thus, targeting the Tre1 family could help us regulate access to the brain.

**Abstract:**

Understanding the evolutionary relationship between immune cells and the blood–brain barrier (BBB) is important to devise therapeutic strategies. In vertebrates, immune cells follow either a paracellular or a transcellular pathway to infiltrate the BBB. In *Drosophila*, glial cells form the BBB that regulates the access of hemocytes to the brain. However, it is still not known which diapedesis route hemocytes cells follow. In vertebrates, paracellular migration is dependent on PECAM1, while transcellular migration is dependent on the expression of CAV1. Interestingly *Drosophila* genome lacks both genes. Tre1 family (Tre1, *moody*, and Dmel_CG4313) play a diverse role in regulating transepithelial migration in *Drosophila*. However, its evolutionary history and origin are not yet known. We performed phylogenetic analysis, together with HH search, positive selection, and ancestral reconstruction to investigate the Tre1 family. We found that Tre1 exists in Mollusca, Arthropoda, Ambulacraria, and *Scalidophora*. *moody* is shown to be a more ancient protein and it has existed since Cnidaria emergence and has a homolog (e.g., GPCR84) in mammals. The third family member (Dmel_CG4313) seems to only exist in insects. The origin of the family seems to be related to the rhodopsin-like family and in particular family α. We found that opsin is the nearest receptor to have a common ancestor with the Tre1 family that has diverged in sponges. We investigated the positive selection of the Tre1 family using PAML. Tre1 seems to have evolved under negative selection, whereas *moody* has evolved during positive selection. The sites that we found under positive selection are likely to play a role in the speciation of function in the case of *moody*. We have identified an SH3 motif, in Tre1 and, *moody* and Dmel_CG4313. SH3 is known to play a fundamental role in regulating actin movement in a Rho-dependent manner in PECAM1. Our results suggest that the Tre1 family could be playing an important role in paracellular diapedesis in *Drosophila*.

## 1. Trans-Epithelial Migration 

The blood–brain barrier (BBB) of the *Drosophila* is homologous to that of the vertebrates. In vertebrates, the main building block of the BBB is known as the nervous vascular unit, and it consists of endothelial cells, basal cells, astrocytes, and pericytes. Immune cells in vertebrates are diverse as they include innate and adaptive immune cells [1]. Immune cells can infiltrate the BBB through either a paracellular route or through a transcellular route [2]. The vertebrates BBB possess various main types of adhesion proteins such as tight junction proteins, junctional adhesion molecules, and cadherins [2,3]. In *Drosophila melanogaster*, the BBB mainly consists of a layer of large glia cells called subperineurial glia (SPG); these cells tightly adhere to each other utilizing septate junctions between the lateral borders of these cells. Regulating access of substances to the *Drosophila* nervous system is essential for its protection from toxic substances and high potassium ion concentrations existing in the surrounding hemolymph. In addition to the membrane proteins neurexinIV, neuroglian, and contactin, *Drosophila* septate junctions contain components of vertebrate tight junctions such as claudin-like proteins [4,5]. 

Both Tre1 and *moody* subfamilies are expressed in the *Drosophila* blood-brain barrier, however, their exact role regarding immune cell migration is not yet clear. Tre1 family includes three homologs; *moody*, Tre1, and CG4313 [6]. It has been shown that in *moody* mutant flies, the BBB was compromised and *Drosophila* manifested behavior abnormalities [4]. Interestingly, in these mutants, large spaces between the SPG cell junctions were reported [4]. Furthermore, *moody* mutant SPG cells were shown to exhibit abnormalities in the actin cytoskeleton [7]. Thus, *moody* was suggested to regulate the actin-rich structure that existed on the borders of the SPG cells [4]. However, its rule in regulating hemocytes migration to the brain has not been yet investigated. Tre1 has been shown to play an important role in transepithelial migration. Tre1 was proposed to be a trehalose receptor [8,9,10]. However, it was demonstrated that the proposed function of Tre1 is done by a neighboring gene (e.g., Gr5a) [9]. It was displayed that Tre1 is essential for *Drosophila* germ transepithelial migration [11]. Interestingly inhibiting small Rho GTPases in germ cells affected transepithelial migration, suggesting that Tre1 signals through Rho1. It was suggested that Tre1 acts in a manner similar to chemokine receptors required during transepithelial migration of leukocytes, implying an evolutionarily conserved mechanism of transepithelial migration. Recently, Tre1 has been identified in the *Drosophila* BBB However, its role is still unidentified [12]. 

The evolutionary history and origin of the Tre1 family are not yet understood. Tre1 has been identified in *Drosophila* [11]. However, if it exists outside the Arthropoda family is not yet known. Tre1 structure is closely related to 7 transmembrane GPCRs [11]. It was reported that there is a high similarity between Tre1 and melatonin as well as histamine, and serotonin receptors among other GPCRs [8,11]. However, the exact GPCR family, where Tre1 belongs is not yet defined. *Moody* expression has been studied extensively in *Drosophila*. Its structure has been identified to follow GPCRs structure [6]. A high similarity has been proposed between GPCR84 and *moody.* However, if they belong to the same family is still not clear. Furthermore, DMEL_GC4313 evolutionary history is also not well recognized. Moreover, the exact role of the Tre1 family in the transmigration of hematocytes into the *Drosophila* brain has still not been defined [12]. Taken together, these observations highlight the need for studying the history of these three family members, to consider their origin, selective pressure, and structure. 

In this research, we employed phylogenetic analysis to assess the origin and the evolutionary history of the Tre1 family. In particular, we performed multiple sequence alignment, tree phylogenetic building, ancestral sequence construction. We then used Blastp and HHSearch to infer the nearest protein that could be related to the Tre1 family (Tre1, *moody*, Dmel_CG4313). After that, we investigated the positive selection, structural similarity, and motif identification in the three subfamilies as well as in the reconstructed originals sequence. We found that *moody* is the most ancient and most continuously expressed gene of the family, being expressed from cnidarian to humans and being subjected to global positive selection. Tre1 was only expressed and identified in invertebrates. Dmel_CG4313 seems to be only in insects. The origin of the family seems to have diverged from a common ancestor of rhodopsin. These results indicate that the Tre1 family belongs to the rhodopsin-like (α family), as they express the NPXXY motif at H7. We identified an SH3 motif that is expressed in Tre1 and *moody*. SH3 is known to interact with Rho in the paracellular diapedesis route in vertebrates. We postulate that Tre1 and *moody* could be performing a regulatory role analogous to PECAM1 in vertebrates by adjusting cell adhesion in the glial layer of the *Drosophila melanogaster*, to regulate the infiltration of hepatocytes. 

## 2. Methods

### 2.1. Database Search

The focus of this research was investigating the relationship between Tre1s’ molecular evolution and their functions. Due to the diverse nature and long evolutionary history, we studied the protein sequences rather than the DNA sequences as they could be more informative. Moreover, to make sure that our analysis is a reasonable representation of Tre1s’ evolutionary history, we chose 14 phyla that span more than 500 million years. *Drosophila* Tre1 protein family was used for BLASTP searches against proteomes of Choanoflagellata, Porifera, Ctenophora, Placozoa, Cnidaria, Chordata, Ambulacraria (Hemi and Echioderma), Scalidophora, Panarthropoda, Nematoda, Rotifera, Chaetognatha, Mollusca, and Annelida. Sequences were designated as candidate proteins if their E values were ≤1 × 10^−10^. Sequences were additionally filtered for having seven transmembrane domains [13,14].

### 2.2. Alignment and Phylogenetic Analysis

The phylogenetic investigation was as follows. First, Tre1 family amino acid sequences were aligned using MAFFT via the iterative refinement method (FFT-NS-i) [2,13]. After that, we built the phylogenetic trees using the maximum likelihood analysis utilizing PHYML implemented in Seaview with five random starting trees [2,15]. 

### 2.3. Ancestral Sequence Reconstruction (ASR) 

To build the ancestral sequence for the Tre1 family and its main families (Tre1, *moody,* and CG4313), we employed the MEGA-X program implantation of the maximum likelihood method. The maximum likelihood method aims at calculating the sites with the highest probability based on the assumption that certain events are more likely to occur (e.g., transition has a higher probability to occur than transversion) [16]. To confirm our results, we used two other methods (i) we employed Blastp BlastP against the nearest earlier diverging organism. BlastP outcome was only accepted if the E-value threshold was less than 10^−10^. (ii) we used the HHsearch method to inspect the evolutionary history of Tre1. Only proteins that have already diverged before Tre1 were considered as candidate parents [17]. The Ancestral sequences evolutionary network was built utilizing SplitsTree with the default setting and bootstrap value of 100 [14,18].

### 2.4. Positive Selection Analysis

To investigate if members of the Tre1 family experienced positive selection during evolution, a maximum likelihood method was utilized [19]. We used the backtranslation function on the EMBOSS server (https://www.ebi.ac.uk/Tools/st/emboss_backtranseq/ (accessed on 5 May 2021) to estimate the cDNA of the investigated sequences [20]. After that, we used CODEML in PAML v4.4 to investigate the positive selection [19,21]. We used three different models namely (basic, branch, and branch-site). (i) in basic mode substitution rate ratio (ω) = nonsynonymous (dN)/synonymous (dS) mutations. In the basic model, ω is calculated globally for all the trees. (ii) In the branch model, two values for ω are calculated, the first one is for the branch being investigated and the other one is global except for the investigate branch. In the third investigation, we explored sites that evolved under positive selection on specific branches, using the M1A vs. M2A and the M7 vs. M8 models. To calculate the statistical significance of these models we used, the likelihood ratio test. 

### 2.5. Linear Motifs Prediction

To study the relationship between molecular evolution and the function of Tre1, we employed the ELM approach. We searched Tre1 family protein sequences for linear motifs one three levels (i) Tre1 (ii) moody (iii) Ancestral sequence of Tre family. Linear motifs are composed of short stretches of neighboring amino acids that could indicate functional sites. We performed the ELM server http://elm.eu.org/ search with a cut-off of 100 [22,23].

### 2.6. Functional Divergence 

Type I functional divergences between gene clusters of the Tre1 family were estimated through posterior analysis using the DIVERGE v2.0 program [24]. Functional type I divergence determines amino acids that are highly different in their conservation between two groups, indicating that these residues have undergone altered functional constraints. The clusters were pairwise compared as described before [21]. 

## 3. Results

### 3.1. Alignment, Phylogenetic and Origin of Tre1 Family

**Figure 1:**
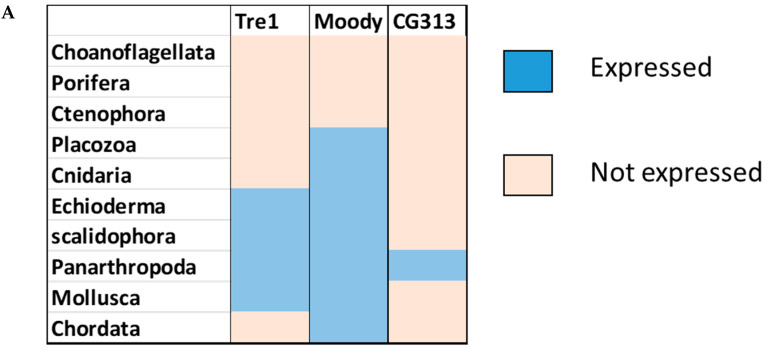

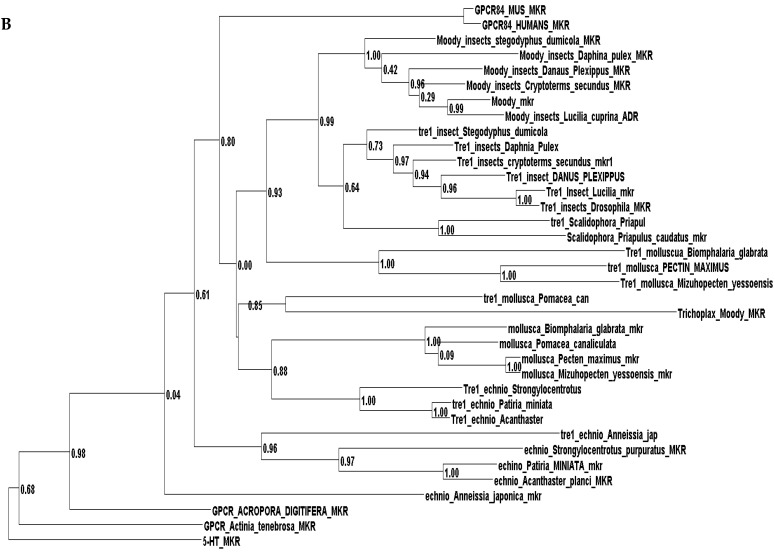
Evolutionary history of the Tre1 family. (**A**) *moody* seems to be the oldest family in the Tre family tree, whereas CG4313 seems to be expressed only in insects. (**B**) The phylogenetic tree shows that *moody* and Tre1 were orthologs that started to diverge in Cnidaria/Trichoplax period emergence.

**Figure 2:**
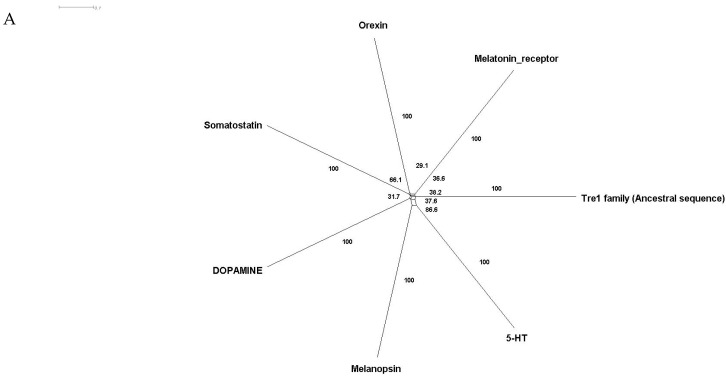

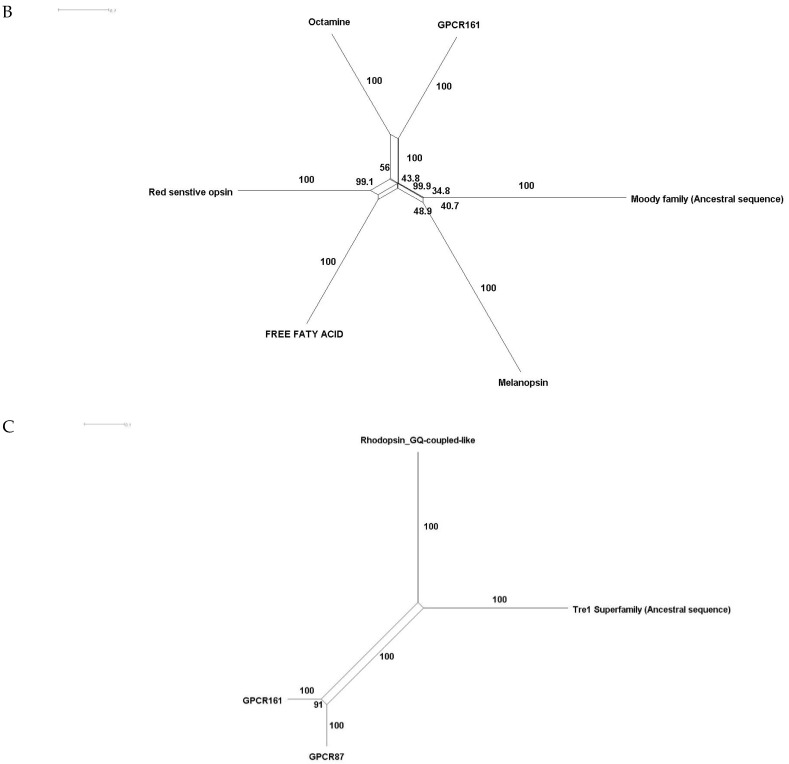
Ancestral sequence construction and network tree for family Tre1. We used the *Amphimedon queenslandica* (Sponge) genome to locate putative common ancestors for the family Tre1 family. (**A**) The nearest common ancestor for Tre1 melatonin, somatostatin, dopamine receptors, and melanoopsin. (**B**) The nearest common ancestors to *moody* divergence are melanopsin and GPCR161. (**C**) Tre1 family nearest common ancestors compromise rhodopsin GQ-coupled, GPCR161, and GPCR87.

Phylogenetic investigations identified Tre1 family members only in invertebrates. We also found various homologs for Tre1 in Ambulacraria specifically in Echinodermata such as *Patiria miniata* (Bat star), *Anneissia japonica* (Sea lilies), *Strongylocentrotus purpuratus* (Pacific purple sea urchin), *Asterias rubens* (common sea star), and *Acanthaster planci* (crown-of-thorns starfish) that was separated from Chordata around 600 MYA as well as various Protostomia that emerged around 610 MYA including (i) Scalidophora such as (*Priapulus caudatus*). (ii) Panarthropoda including *Stegodyphus dumicola*, (African social spider), *Daphnia pulex* (water flea), *Lucilia cuprina* (Australian sheep blowfly), *Danaus plexippus* (Monarch butterfly), and *Cryptotermes secundus* (Termite). (iii) In Mollusca, we identified Tre1 homolog in Bivalves *Mizuhopecten yessoensis* (Japanese scallop), *Pecten maximus* (great scallop), *Pomacea canaliculata,* (golden apple snail), and *Biomphalaria glabrata* (Bloodfluke planorb). Tre1 also does not seem to have any homologs in Placozoa or Cnidaria, Ctenophora, Porifera, or choanoflagellates (Figure 1). For CG4322 (*moody*), we were able to find it in Placozoa, Cnidaria, Ambulacraria, Scalidophora, Panathropdoa, Mollusca, and Chordata. We were able to locate *moody Drosophila* homolog in Cnidaria and specifically in *Acropora Digitifera* (Staghorn coral) and *Actinia tenebrosa,* commonly (Waratah anemone). Similar to Tre1, we were able to locate *moody* homologs in Ambulacraria including Echinodermata such as *Patiria miniata* (Bat star), *Anneissia japonica* (Sea lilies), *Strongylocentrotus purpuratus* (Pacific purple sea urchin), *Asterias rubens* (common sea star), and *Acanthaster planci* (crown-of-thorns starfish) and Protostomia that emerged around 610 MYA including (i) Scalidophora such as (*Priapulus caudatus*). (ii) Panarthropoda including *Stegodyphus dumicola*, (African social spider), *Daphnia pulex* (water flea), *Lucilia cuprina* (Australian sheep blowfly), *Danaus plexippus* (Monarch butterfly), and *Cryptotermes secundus* (Termite). (iii) In Mollusca, we identified Tre1 homolog in Bivalves (*Mizuhopecten yessoensis* (Japanese scallop, *Pecten maximus* (great scallop), *Pomacea canaliculata*, (golden apple snail), and *Biomphalaria glabrata* (Bloodfluke planorb). We were only able to locate CG4313 in insects. It does not seem to have homologs in Placozoa, Cnidaria, Ambulacraria, Scalidophora, Mollusca or Chordata. Notably, our results indicate that Tre1 nearest common ancestors in Sponge are melatonin, dopamine, and melanopsin. In the case of moody, the nearest common ancestors are melanopisn, GPCR161, and Octamine. Interestingly, in the case of the Tre1 family ancestral sequence, the nearest common ancestors are opsin-GQ coupled and GPCR161 (Figure 2) (Supplementary File S1).

### 3.2. Positive Selection Investigation

Our results suggested that the Tre1 family has a low variation in relation to its evolutionary selection. We employed global, branch, and branch-site models in the CODEML program of PAML v4.4 to examine whether members of the Tre1 family underwent positive selection. Our results show that *moody* was under positive selection with a ω value of 1.18 and *p* < 0.001. The Tre1 family was not subjected to positive selection, indicating that Tre1 could be highly conserved in invertebrates. Similarly, there was little detected variation among branches of Arthropoda among *moody* members (Table 1).

### 3.3. Structural Analysis

**Figure 3:**
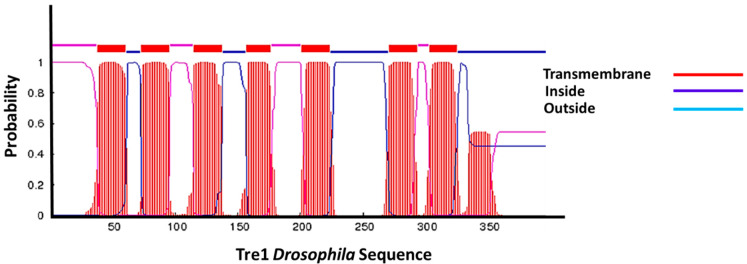
Tre1 in transmembrane structure. Tre1 in *Drosophila* possesses seven transmembrane regions common in GPCRs.

The Tre1 sequences had a high degree of similarity on different levels, including structural, motif, GPI function, as well as specificity of their residues. On the structural level, all Tre1 family members shared seven transmembrane domains (Figure 3). We also identified several motifs that were shared by more than one family such as VNXSXG, which is a Generic motif for N-glycosylation. It was shown that Trp, Asp, and Glu are uncommon before the Ser/Thr position. Efficient glycosylation usually occurs when ~60 residues or more separate the glycosylation acceptor site from the C-terminus. Additionally, we identified the RLTXMMLX, which is an A-kinase docking motif that mediates interaction towards the ERK1/2 and p38 subfamilies of MAP kinases. Moreover, we identified the RXHATTAFV motif which is canonical Arg-containing phospho-motif mediating a strong interaction with 14-3-3 proteins. Importantly we identified ASSVINP which is a motif recognized by SH3 domains with a non-canonical class I recognition specificity. We were also able to locate several variants of YGNV which is a tyrosine-based sorting signal responsible for the interaction with the mu subunit of the AP (adaptor protein) complex (Table 2). In *moody,* we found YTTIG which is an NCK Src Homology 2 (SH2) domain binding motif, YTKKF which represents a STAP1 Src Homology 2 (SH2) domain Class 2 binding motif. We also located class I SH3 domains (e.g., KSHPTLP and RYSPPSP). We were also able to identify PTLPTR and PPSPIR which are recognized by class II SH3 domains. Additionally, we identified, NTSVVWP which is a motif recognized by those SH3 domains with a non-canonical class I recognition specificity. Moreover, we located two motifs recognized by WW domains of Group I (e.g., PPQY and PPLY). 

### 3.4. Functional Specificity

**Figure 4:**
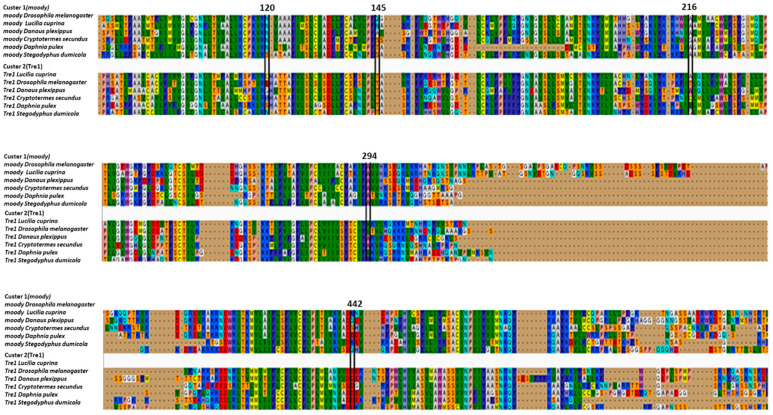
Functional divergence of the Tre1 family. Putative sites that caused a change of function between Tre 1 family and Moody family.

We employed Diverge to determine positions that were well conserved within the Tre1 family in insects but differed between its main members Tre1 and *moody*. Notably, we were able to identify only five locations where putative functional specificity has taken place. Namely at position 120, moody has N, while Tre1 has C/E/G/H/S/N. At position 145, Tre1 has T, while moody has Q/N/A/S/T/S. AT position 216 Moody has A/G, while Tre1 gas A/T/W/C/M/G. At position 294, moody has W, while Tre1 has V/I/W/Y. Also at the position, 442, Tre1 has D, while moody has K/Q/S/E (Figure 4).

## 4. Discussion

Analysis of the Tre1 family from a phylogenetic perspective provided the basis for understanding its functional diversity. Phylogenetic analysis was conducted to trace the evolutionary history of the Tre1 family in 14 phyla. We also conducted a positive selection, functional specificity, and functional divergence analysis to investigate the evolutionary history of the Tre1 family function. Furthermore, we investigated structural motifs among Tre1 family members. The Tre1 family includes three main subfamilies, namely Tre1, *moody*, and CG4313. *moody* first diverged during Cnidaria emergence and it is expressed in mammals. Tre1 has a shorter evolution history as it first appeared in Nephrozoa in both Ambulacraria and Protostomia (Ecdysozoa and Spiralia) but not in Chordata. CG4313 is only found in insects. We found that the nearest genes that could constitute a putative origin for the Tre1 family are opsin, olfactory receptors, and GPCR161. However, Blastp and structural analysis show that the nearest candidate to have a common ancestor with the Tre1 family is opsin. On an individual family level, we found Tre1 melatonin and *moody* are nearest to melanopsin. Positive selection analysis showed that only the *moody* family was under positive selection. The rest of the Tre1 was shown not to be under positive selection. This suggests that the function of Tre1 could be tightly conserved among the species that express it. On a structural level, the Tre1 family consists of seven transmembrane domains with the main motif of the rhodopsin-like family (NPY). The Tre1 family shares a trypsin-sorting signal protein that interacts with the mu subunit of the adaptor signal. They also express the SH3 that was shown to play an important role in BBB integrity. The Tre1 family is expressed in the BBB of insects and is likely to represent a step toward complexity in controlling access of nutrients and cells to the brain.

### 4.1. Evolution History of the Tre1 Family 

The Tre1 family has a diverse evolutionary history in invertebrates as well vertebrates. Our phylogenetic analysis suggests that the Tre1 family (Tre1, *moody,* and CG4313) has first appeared in Cnidaria. Investigating the ancestral sequence of Tre1 showed that the nearest sequence for it to be melatonin, somatostatin, and 5-HT receptor. The ancestral sequence of *moody* includes melanopsin, free fatty acid, and red-sensitive opsin (Figure 2). Tre1 family does not have any similarity with previously suggested families such as trehalose [9]. Tre1 family seems to have diverged from a common ancestor of a rhodopsin-like transmembrane protein. The ancestral sequence of the Tre1 family is similar to opsin, olfactory receptor, and GPCR161 (Figure 2). Investigating the common motif and similarity (Figure 3), suggest that opsin is a higher probable candidate to be the nearest protein diverging within the ancestral sequence. This is also supported by Blastp (<10^−10^). These findings are in agreement with a previous report that linked the rhodopsin-like family to opsin [14]. It has been suggested that in metazoan, the first opsin originated from the duplication of the common ancestor of the melatonin and opsin genes in a metazoan (Placozoa plus Neuralia) ancestor [25]. Other reports suggested a link between fungi and metazoan opsin that first appeared 1300 Mya. Whether this ancestral protein was capable of processing light is still not clear. Interestingly, it has been suggested that dipterans possess an ancestral set of five core opsins which have undergone several lineage-specific events including an independent expansion of low wavelength opsins. If any of the Tre1 family members could be implicated in light sensitivity is still unknown. Interestingly histamine was reported to activate the Tre1 receptor [26]. Whether it is the only GPCR ligand to be able to bind to the Tre1 receptor is unknown. We can postulate that various ligands could be able to activate Tre1 due to the promiscuous nature of the receptor in lower invertebrates [14]. We could not locate Tre1 in any vertebrates, and CG4313 was only confined to insects (Figure 1). Interestingly, *moody* has been found in mice and humans (Figure 1) along with Cnidaria and Echinodermata. Thus, *moody* seems to have a continuous existence between invertebrates and vertebrates. Taken together, our results indicate that the Tre1 family diverged from an opsin-like protein into *moody*, later Tre1 appeared in Nephrozoa, while CG4313 is only expressed in insects. 

### 4.2. Structural Evolution and Functional Divergence

The variation in the physiological functions of different groups of the Tre1 family could be influenced by possessing functional specific residues, functional divergent residues, being subjected to positive selection, or a combination of these factors. Our positive selection analysis showed that *moody* evolved under positive selection. This indicates that it could possess various and additional functions in higher vertebrates. However, positive selection per branch was not significant (Table 1). Type I functional divergence is the result of the change in evolutionary rate where a site is conserved for one group and is variable for another. Type 1 analysis showed five positions that could be critical in functional speciation (Figure 4). Interestingly, one site is entirely different between Tre1 and moody. This site is at position, 442, where Tre1 has D, while moody has K/Q/S/E. This site is located in the last segment of the transmembrane inside the cell. These observations indicate that there is variation among Arthropoda species in which moody is expressed. On the other hand, conservation of the aspartic acid amino acid in Tre1 expressed among various species of insects, indicates that it could be performing an important role in the downstream pathway of the transmembrane. The low number of functional divergence sites indicate that the Tre1 and *moody* could be sharing conserved functions in insects. 

### 4.3. Role of Tre1 and Moody in BBB 

High similarity in evolutionary patterns, expression profiles, and structure between Tre1 groups hints at a possible role in Arthropoda brain evolution in controlling BBB diapedesis. *Drosophila* has a brain-like structure as well as immune cells-like known as hemocytes and BBB barrier-like structure known as subperineurial glial cells (SPG) [27,28]. The hemolymph–brain barrier of *Drosophila* is established by surface glia, which insulates the nerve cord against the potassium-rich hemolymph by forming intercellular septate junctions. However, the diapedesis mechanism in *Drosophila* is still unknown. In lower vertebrates such as Trichoplax, no immune cells were found, and one rudimentary brain or neural cell was detected. Interestingly, CAV1 was found to be expressed in Trichoplax. CAV1 is likely to be used to construct caveolae for food transfer and its function has evolved to include cells later after the Cambrian explosion. This is supported by the observation that a rudimentary brain-like structure has been reported in nematostella, with two brain pathways observed and immune-like cells known as amoebocytes. Interestingly, in Cnidarian, only CAV1 has been detected and not PECAM1. In *C. elegans*, no immune cells were detected. However, a glial BBB-like structure was observed, together with CAV1. We as well as others have shown that CAV1 and PECAM1 play an indispensable role in BBB diapedesis [2]. Interestingly, *Drosophila melanogaster* does not express either CAV1 or PECAM1 which are vital for transcellular and paracellular diapedesis. Tre1 was reported to direct transepithelial migration of *Drosophila* germ cells by regulating E-cadherin [11,29]. It has also been shown to orient stem cell divisions in the Drosophila central nervous system [30], control of germ cell polarity probably through controlling actin dynamics [31,32]. Interestingly, Tre1 was reported to play an important part in regulating the extravasation of hemocytes in *Drosophila* [33]. Tre1 was also shown to be expressed in the *Drosophila* hemolymph–brain barrier [6,12]. However, its function is not yet known. Additionally, moody controls blood–brain barrier permeability in *Drosophila* [6]. It has been shown that moody, the G protein subunits Gαi and Gαo, and the regulator of G protein signaling Loco are required in the surface glia to achieve effective insulation by regulating the cortical actin and thereby stabilizing the extended morphology of the surface glia. SH3 domains are small protein modules of about 50 to 60 residues that seem to play a part in the BBB integrity [34]. It is well known that SH3 through the Rho pathway control actin movement in paracellular through PECAM1 mediated fashion [35,36]. Thus, we could postulate that Tre1 and moody could be playing a similar role in *Drosophila.* Interestingly, Tre1 and moody are both expressed in PM1, PM2, and PM7 of *Drosophila* blood cell types (Supplementary File S2). Based on the observations that Tre1 and moody have limited sites of functional divergence and are co-expression in blood and the BBB. Gene evolutionary redundancy is not uncommon [37]. It could be related to Tre1 and moody having several common functions and a limited number of different functions [37]. It has been postulated that the reason for that could be that random mutations have a higher probability to terminate all functions of a gene, rather than destroy a single redundant function while not affecting other functions [37].

### 4.4. Limitations of Our Investigation 

Currently, experimental validation is still needed to support our in silico-based results. Our investigation hints that the Tre1 family could be playing a role in directing hemocytes infiltration of the BBB, based on several pieces of evidence. Tre1 was shown to play a part in the extravasation of hemocytes in *Drosophila* [33]. Moreover, Tre1 has been shown to regulate the migration of cells in the germ cells and to be expressed in the BBB in *Drosophila* [29] Furthermore, PECAM1 and CAV1 are not expressed in *Drosophila*. However, the PECAM1 function is mediated by the existence of SH3 that controls the actin-Rho pathway [34,35,36]. Interestingly, our analysis shows that the Tre1 family (Tre1 and *moody*) both possess an SH3 motif. Thus, there is a possibility that it could be playing a role in regulating BBB infiltration. However, experimental confirmation is still necessary to build on our hypothesis. 

## 5. Conclusions 

The Tre1 family is a heterogonous family that seems to have diverged from opsins during Cnidarian emergence. The main members of the family (Tre1 and *moody*) seem to have undergone different evolutionary patterns, whereas Tre1 was conserved, moody was subjected to positive selection and through its homolog has covered a period from Cnidarian to Humans. Tre1, on the other hand, is only expressed in a limited number of invertebrates. Nevertheless, both genes possess SH3 associated domains as well as glycosylation sites and seem to be to interact with the Rho-actin pathway in a form similar to PECAM1. Both genes are expressed in the *Drosophila* brain–blood barrier. Thus, we could postulate that the Tre1 family could be performing a role in regulating hemocytes access to *Drosophila* brain-like structure. 

## Figures and Tables

**Table 1:** Likelihood values and parameter estimates for Tre1 genes under positive selection.

Test Type	Target	ω	*p*-Value
M0-global	Tre1	1	Not significant
M0-global	moody	1.189	<0.01
Branch	Arthropoda (Moody)	0.97	Not significant
	Humans (GPCR84)	1.18	Not significant
Branch	Arthropoda (Tre1)	1.19	Not significant
Branch-site	Arthropoda (Moody)	>1	Not significant
	Tre1	>1	Not significant

**Table 2:** Motif shared by Tre1 family.

Gene	Motif	*p*-Value
Tre1	VNXSXG	5.02 × 10^−3^
	RLTXMMLXI	2.58 × 10^−3^
	RXHATTAFV RLTXMMLXI RQYSESIFYF	4.48 × 10^−3^
	ASSVINP	1.32 × 10^−2^
	YGNV YILI YXXI YXCI YXXV YXXL	2.59 × 10^−3^
*moody*	YTTIG	8.2 × 10^−4^
	YSPPS	
	YTKKF	1.0 × 10^−3^
	KSHPTLP	1.2 × 10^−3^
	RYSPPSP	
	PTLPTR	1.1 × 10^−3^
	PPSPIR	
	NTSVVWP	1.3 × 10^−2^
	PPQY	1.2 × 10^−4^
	PPLY	

## Data Availability

Data used is available as Supplementary Files.

## Supplementary Materials

The following are available online at https://www.mdpi.com/article/10.3390/insects12100882/s1, supp_file1_adera and it contains all the FASTA files for all proteins investigated in this investigation. supp_file2_adera contains the expression of Tre1 and moody in *Drosophila* single cell data clusters.
