# Supplementary material for: Investigation of Evolutionary History and Origin of the Tre1 Family Suggests a Role in Regulating Hemocytes Cells Infiltration of the Blood–Brain Barrier
Source: Insects. 2021 Sep 29;12(10):882. doi: 10.3390/insects12100882 (PMC8540695; doi:10.3390/insects12100882)
Supplement: Supplementary file 1 [file insects-12-00882-s001.zip › suppl_files_2_adera.zip!/DRSC RNA Seq Browser_cg4313_adera.pdf]

Integration of Unwounded, Wounded, and Wasp infested 24 hr

Project ID: 26 *Sudhir Tattikota*

Coordinate Ids

tSNE

Submit

Select Condition / Sample

- (•) Wounded
- ( ) Unwounded
- ( ) Wasp infested 24 hr
- ( ) all

Submit

---

tSNE

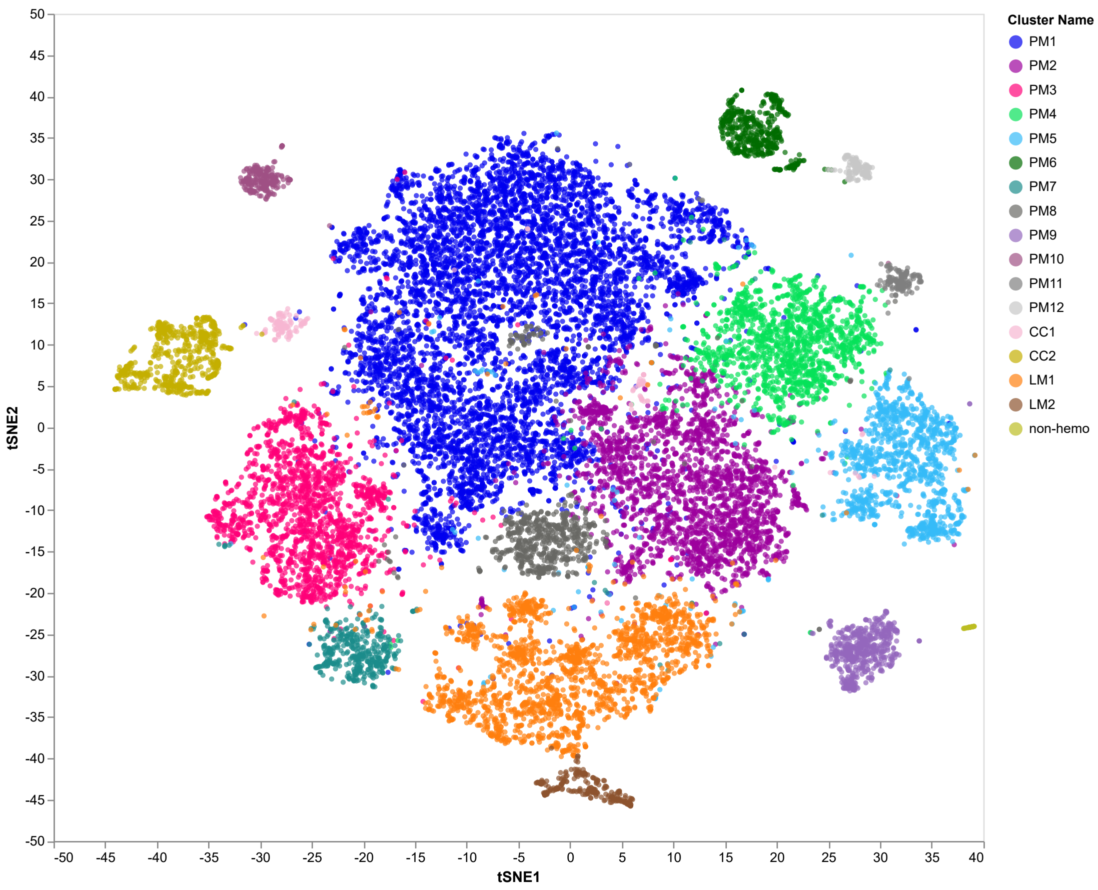

stack / unstack graphs

- Gene Lookup cg4313 =>FBgn0025632 (https://www.flybase.org/reports/FBgn0025632)
- Genes In Results: FBgn0025632 (https://www.flybase.org/reports/FBgn0025632)

Bee Chart (/tools/rna_seq_sgt2/web/violin_chart/26/plot_coord=0/sample_id=all/cg4313)

Gene Names (up to 5 comma separated)

cg4313

Search

tSNE

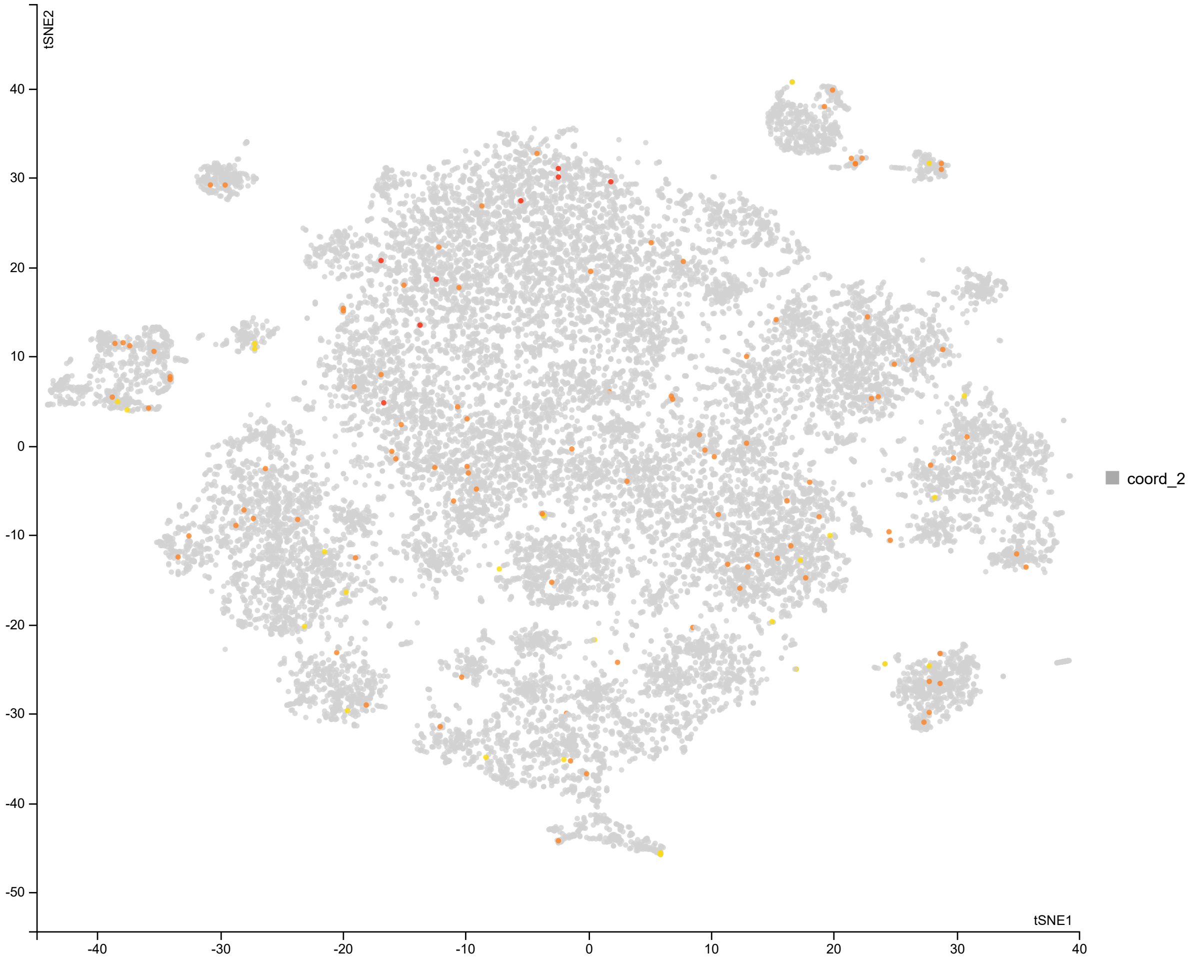

export graph (png) 2500 export width px

export graph (svg)

log normalized UMI:

<=0 0 1 2 4

Adjust:

0 -> 0

0 -> 1

1 -> 2

2 -> 4

4 -> 8

Reset Values

Summary of Cells Expressing Selected Genes

Gene	PM1	PM2	PM3	PM4	PM5	PM6	PM7	PM8	PM9	PM10	PM11	PM12	CC1	CC2	LM1	LM2	non-hemo
FBgn0025632	36 (0.5%)	19 (0.8%)	11 (0.6%)	8 (0.5%)	9 (0.7%)	7 (1.4%)	4 (1%)	3 (0.5%)	6 (1.2%)	2 (0.8%)	0	3 (2.3%)	4 (2.6%)	10 (1.6%)	8 (0.4%)	3 (1.1%)	0
Total Cluster Count	6796	2500	1810	1461	1204	500	421	630	512	243	148	131	154	643	1993	272	40

All Cell Count Summary

Sample Name	PM1	PM2	PM3	PM4	PM5	PM6	PM7	PM8	PM9	PM10	PM11	PM12	CC1	CC2	LM1	LM2	non-hemo
all	6796	2500	1810	1461	1204	500	421	630	512	243	148	131	154	643	1993	272	40
Unwounded	1546	604	315	377	288	3	44	141	171	86	47	0.	26	125	311	26	0.
Wasp infested 24 hr	1987	631	731	534	438	342	183	144	19	21	42	0.	36	162	735	125	40
Wounded	3263	1265	764	550	478	155	194	345	322	136	59	131	92	356	947	121	0.

Data Table

Cell Barcode	Cluster Name	FBgn0025632
WASP96hr_dropseq_PI24CIRC2_GGATATTGAATA.2	PM1	0.743839025497437
WASP96hr_dropseq_PI24CIRC2_CTAGGTATGGTA.2	PM1	0.8958780169487
injured_indrop_PC2_blood3_TCGCATAA.CTTAGTGT.CATCGCAG	PM1	1.13323998451233
WASP96hr_dropseq_PI24CIRC3_TCAGATGCGCGG.3	PM1	1.1588499546051
injured_tenx_B4_CACAAACGTCGCATAT	PM1	1.29542005062103
UNinjured_indrop_UC1_blood2_TAAGGCTC_CCAGCGAA_AGGATTCA	PM1	1.46403002738953
UNinjured_indrop_UC1_blood2_TAAGGCTC_ATATGGGT_GGGAGGTA	PM1	1.49331998825073
injured_tenx_B4_GCTGCAGGTACTTAGC	PM1	1.50276005268097
UNinjured_indrop_UC1_blood2_TAAGGCTC_TCATAGCT_AAACTCGA	PM1	1.53794002532959
injured_tenx_B4_CAGCAGCGTGGGTATG	PM1	1.55953001976013
UNinjured_indrop_UC1_blood2_TAAGGCTC_AGGCAATA_TTAGTCCG	PM1	1.56340003013611
UNinjured_indrop_UC1_blood2_TAAGGCTC_CTGGAACA_GTGTAACC	PM1	1.58493995666504
injured_indrop_PC2_blood3_TCGCATAA.CGATGGCA.CACAGTTT	PM1	1.59300005435944
injured_tenx_B2_TGCCAAAGTAAGTGGC	PM1	1.63821005821228
injured_indrop_PC2_blood3_TCGCATAA.GGAGGTTT.AAGTAATC	PM1	1.64425003528595
injured_indrop_PC1_blood2_CTTAATAG.GGGCATCA.GTTGTCAT	PM1	1.64898002147675
injured_indrop_PC1_blood2_CTTAATAG.GGGAGATG.GGTCACAG	PM1	1.65170001983643
injured_indrop_PC2_blood3_TCGCATAA.AGGCGTTT.ACATCTAT	PM1	1.66930997371674
injured_indrop_PC2_blood3_TCGCATAA.CTTTCACT.CTTCTTCG	PM1	1.76317000389099
injured_indrop_PC2_blood3_TCGCATAA.GTATTGAC.TCGGTTTA	PM1	1.76718997955322
UNinjured_indrop_UC1_blood2_TAAGGCTC_CACTTGAT_ATGCGGAT	PM1	1.78718996047974
UNinjured_indrop_UC1_blood2_TAAGGCTC_CTGAAGGG_CTAGATTG	PM1	1.82196998596191
injured_indrop_PC2_blood3_TCGCATAA.AGCTTCGA.TTAGGACC	PM1	1.85531997680664
injured_tenx_B4_CTGCCTAAGCGTGTCC	PM1	1.86033999919891
injured_indrop_PC1_blood2_CTTAATAG.ATTATTCG.ATATCTTC	PM1	1.90764999389648
injured_indrop_PC2_blood3_TCGCATAA.GCATCACT.AGAGACTA	PM1	1.91402995586395
injured_indrop_PC2_blood3_TCGCATAA.CTTCTACG.TCCTTATT	PM1	1.92744994163513
injured_indrop_PC2_blood3_TCGCATAA.TCCAGAAG.TAGAAATG	PM1	1.98587000370026
WASP96hr_dropseq_PI24CIRC3_ATACCGGGTAAC.3	PM1	2.0368800163269
injured_tenx_B2_ACCAGTAGTTATCGGT	PM1	2.03920006752014
UNinjured_indrop_UC2_blood3_TAAGGCTC_CGTGTACA_ATCCGCTA	PM1	2.12456011772156
WASP96hr_dropseq_PI24CIRC3_GTTTACCCGGCN.3	PM1	2.12911009788513
injured_indrop_PC1_blood2_CTTAATAG.GGAGTAAG.TACAGCCG	PM1	2.15903997421265
UNinjured_indrop_UC2_blood3_TAAGGCTC_GGTGTCGA_AAGCTTCT	PM1	2.21884989738464
UNinjured_indrop_UC2_blood3_TAAGGCTC_CCGACTTT_GCACCTCT	PM1	2.41256999969482
UNinjured_tenx_B1_GTAGTCACACACTGCG	PM1	2.70061993598938
UNinjured_tenx_B3_AACCATGCAAGTCATC	PM6	0.0479921996593475
injured_indrop_PC2_blood3_TCGCATAA.GTTCTGCT.CCCGACTT	PM6	1.08481001853943

Cell Barcode	Cluster Name	FBgn0025632
injured_indrop_PC1_blood2_CTTAATAG.ACATGGAC.TGTGGCTA	PM6	1.14669001102448
injured_indrop_PC1_blood2_CTTAATAG.TCTAAACT.TTCGCTGG	PM6	1.31960999965668
injured_indrop_PC1_blood2_CTTAATAG.ATTGAAGT.ACGGTAGC	PM6	1.33056998252869
injured_indrop_PC1_blood2_CTTAATAG.CGTGGGAT.TTCGTTCC	PM6	1.33653998374939
injured_indrop_PC2_blood3_TCGCATAA.CGTGTACA.GGGTTGGT	PM6	1.39657998085022
WASP96hr_dropseq_PI24CIRC2_GAACTCGTTGGC.2	PM7	0.488701999187469
injured_indrop_PC2_blood3_TCGCATAA.ACATCTCG.TGCTCCGT	PM7	0.795082986354828
WASP96hr_dropseq_PI24CIRC2_GCTCATCGGTGN.2	PM7	1.2073700428009
injured_indrop_PC2_blood3_TCGCATAA.GCTACCGT.GTAATCCC	PM7	1.47045004367828
injured_tenx_B4_CTCCTAGGTAAATGAC	LM2	0.453152000904083
injured_tenx_B2_GGCGTGTAGTTAACGA	LM2	0.986805975437164
injured_indrop_PC1_blood2_CTTAATAG.AGGTAAGC.TCCGTCCA	LM2	1.324049949646
injured_tenx_B4_AGGGAGTAGTGGGTTG	PM10	1.50411999225616
injured_indrop_PC1_blood2_CTTAATAG.GGCAAGGT.CCACATTA	PM10	1.55303001403809
UNinjured_tenx_B1_TATGCCCTCCTGCAGG	CC1	0.870747983455658
injured_tenx_B2_CTCATTACAGGGTTAG	CC1	0.922241985797882
injured_tenx_B2_ACGATACTCTGCAAGT	CC1	1.12558996677399
injured_tenx_B2_AACGTTGCATGTTCCC	CC1	1.6855800151825
injured_tenx_B4_CGCTATCCAGTCCTTC	PM12	0.726764023303986
injured_tenx_B4_ATCATCTTCGTAGATC	PM12	1.03808999061584
injured_tenx_B4_ATGCGATGTTTCCACC	PM12	1.30984997749329
WASP96hr_dropseq_PI24CIRC2_CAGTGCGATACA.2	PM2	0.48471599817276
WASP96hr_dropseq_PI24CIRC3_AACTCCCCATTA.3	PM2	0.57151597738266
UNinjured_tenx_B3_TCGTACCGTCAAAGCG	PM2	0.985225021839142
injured_tenx_B4_AAGGAGCAGGGTATCG	PM2	1.0650999546051
UNinjured_indrop_UC1_blood2_TAAGGCTC_CGGCTGTA_CAGTCCCT	PM2	1.11117005348206
UNinjured_indrop_UC1_blood2_TAAGGCTC_ATCAATCG_TCTCAACC	PM2	1.13095998764038
UNinjured_indrop_UC1_blood2_TAAGGCTC_TGGGAATT_GTATGAGT	PM2	1.18657004833221
injured_indrop_PC2_blood3_TCGCATAA.ACTAGAGC.ACGGTAGC	PM2	1.19117999076843
injured_tenx_B2_AGATCTGAGGACTGGT	PM2	1.19324994087219
UNinjured_indrop_UC1_blood2_TAAGGCTC_GATACGCA_GACAGATA	PM2	1.32546997070312
injured_indrop_PC2_blood3_TCGCATAA.AGACCTCA.TGGTATGA	PM2	1.37059998512268
UNinjured_indrop_UC1_blood2_TAAGGCTC_AGGGACTG_AACCTGAC	PM2	1.41400003433228
injured_tenx_B2_AAGACCTAGATGTTAG	PM2	1.46480000019073
injured_tenx_B4_TAAGAGATCGGAAATA	PM2	1.48113000392914
injured_indrop_PC2_blood3_TCGCATAA.TGAATAGG.TTAAACTG	PM2	1.48958003520966
injured_indrop_PC2_blood3_TCGCATAA.CCATGCAT.GGTTTCTC	PM2	1.58648002147675
UNinjured_indrop_UC1_blood2_TAAGGCTC_CTGCGATG_GTGTAACC	PM2	1.69386994838715
injured_indrop_PC1_blood2_CTTAATAG.GTCTAATC.TCGAAGCT	PM2	1.77732002735138
injured_indrop_PC2_blood3_TCGCATAA.CTGCTAGA.TCACCGAG	PM2	1.93704998493195
injured_tenx_B4_GATCGATAGAAACGAG	LM1	0.671895980834961
injured_indrop_PC1_blood2_CTTAATAG.CTGAGCGT.AACCCTTG	LM1	0.902099013328552
WASP96hr_dropseq_PI24CIRC3_CTTCGCGTAAGA.3	LM1	1.23917996883392
injured_indrop_PC1_blood2_CTTAATAG.TGCACCAG.GCCGGATT	LM1	1.27073001861572
injured_tenx_B4_CAACCTCTCCACGACG	LM1	1.35695004463196
injured_tenx_B2_AACTCCCGTACCTACA	LM1	1.3625500202179

Cell Barcode	Cluster Name	FBgn0025632
injured_indrop_PC1_blood2_CTTAATAG.AGTTGAAC.CGTAGAAG	LM1	1.42660999298096
injured_indrop_PC2_blood3_TCGCATAA.CCATGCAT.CCAACCGT	LM1	1.54282999038696
WASP96hr_dropseq_PI24CIRC2_TGCCTCATCTCG.2	PM3	0.584113001823425
WASP96hr_dropseq_PI24CIRC2_ACCGGCCGTGTC.2	PM3	0.662064015865326
injured_tenx_B4_AGATCTGTCAAGAAGT	PM3	0.962557971477509
WASP96hr_dropseq_PI24CIRC2_TGCACCCCTTCG.2	PM3	1.06187999248505
injured_tenx_B2_ACATACGTCATTATCC	PM3	1.11486995220184
injured_indrop_PC1_blood2_CTTAATAG.CTTTGGAC.CGAATAAT	PM3	1.45615994930267
injured_tenx_B4_GGCGACTAGGCTCAGA	PM3	1.54543995857239
injured_indrop_PC1_blood2_CTTAATAG.GGAGTAAG.GGTCACAG	PM3	1.57181000709534
injured_indrop_PC1_blood2_CTTAATAG.CGTTGCCT.GAAGCACT	PM3	1.58247005939484
injured_tenx_B4_TGCGGGTGTTTGTTGG	PM3	1.76236999034882
WASP96hr_dropseq_PI24CIRC2_CGAACTGAGGCC.2	PM3	1.85032999515533
injured_tenx_B4_CTGATAGGTTTGTGTG	PM4	1.23345994949341
injured_tenx_B4_GAAGCAGAGTGTGAAT	PM4	1.33364999294281
UNinjured_indrop_UC1_blood2_TAAGGCTC_AGCTCCAC_CCTCGTCT	PM4	1.39134001731873
injured_tenx_B4_TGGGAAGCAGTCCTTC	PM4	1.52178001403809
UNinjured_indrop_UC1_blood2_TAAGGCTC_AAGAGTAT_AACAATCC	PM4	1.52937996387482
UNinjured_indrop_UC1_blood2_TAAGGCTC_AAAGAAAG_AAGTAATC	PM4	1.57818996906281
injured_indrop_PC2_blood3_TCGCATAA.CCGAGATC.CGCTAGTC	PM4	1.60179996490479
UNinjured_indrop_UC1_blood2_TAAGGCTC_CACAAGTA_CTCAGAAT	PM4	1.8215399980545
injured_tenx_B2_ATTACTCAGTGGTAGC	PM5	0.616113007068634
WASP96hr_dropseq_PI24CIRC3_GACCGAGCCTAA.3	PM5	0.808368980884552
injured_tenx_B4_TCTATTGTCCAAACAC	PM5	1.00720000267029
injured_indrop_PC2_blood3_TCGCATAA.CTGAAGGG.CAACGTGG	PM5	1.0783599615097
injured_indrop_PC2_blood3_TCGCATAA.CACTAACC.ACGCTCAG	PM5	1.13480997085571
UNinjured_indrop_UC1_blood2_TAAGGCTC_AGAAACCA_TGCTCCGT	PM5	1.28510999679565
UNinjured_indrop_UC1_blood2_TAAGGCTC_CTAACGCC_AAATCGTT	PM5	1.31800997257233
UNinjured_indrop_UC1_blood2_TAAGGCTC_CTCACATC_AGAACGGG	PM5	1.53965997695923
injured_indrop_PC2_blood3_TCGCATAA.TCAAGGCG.CCGCTGTT	PM5	1.85576999187469
injured_tenx_B2_GATGAAAGTGCGCTTG	CC2	0.589569985866547
injured_tenx_B4_CGATGGCCACACAGAG	CC2	0.602934002876282
UNinjured_tenx_B1_CATCAAGGTCGTCTTC	CC2	1.04186999797821
injured_indrop_PC2_blood3_TCGCATAA.TCCAGAAG.TGTCTTTC	CC2	1.16624999046326
injured_indrop_PC1_blood2_CTTAATAG.CTCCTCCA.AAACCTCC	CC2	1.35652005672455
injured_indrop_PC2_blood3_TCGCATAA.CAAGGTAC.TGGCCTGT	CC2	1.39110994338989
WASP96hr_dropseq_PI24CIRC3_AGGTCTAGAATG.3	CC2	1.42926001548767
injured_indrop_PC2_blood3_TCGCATAA.TCAGCCTC.GGCGTTAG	CC2	1.46556997299194
UNinjured_indrop_UC1_blood2_TAAGGCTC_TCAGCCTC_CTCAGAAT	CC2	1.59019994735718
injured_indrop_PC1_blood2_CTTAATAG.ATTTCCAT.GTTGTCAT	CC2	1.73109996318817
WASP96hr_dropseq_PI24CIRC3_GTGCCGAAGACG.3	PM8	0.617842018604279
WASP96hr_dropseq_PI24CIRC3_TTGCTGGTTCCT.3	PM8	0.845929026603699
injured_tenx_B2_TGCTACCCACTTAACG	PM8	1.72462999820709
UNinjured_indrop_UC1_blood2_TAAGGCTC_CTGAAGGG_GAACGATT	PM9	0.962657988071442
UNinjured_indrop_UC1_blood2_TAAGGCTC_ATCCTAGT_CAGATTAC	PM9	1.06985998153687
UNinjured_indrop_UC1_blood2_TAAGGCTC_AAACTGTG_TTCCGCTC	PM9	1.33056998252869

Cell Barcode	Cluster Name	FBgn0025632
injured_tenx_B2_ATCATGGGTAAATGTG	PM9	1.52995002269745
injured_indrop_PC2_blood3_TCGCATAA.CCACCTCT.TGAAATGA	PM9	1.74072003364563
injured_tenx_B2_ACTGAACTCCACTCCA	PM9	1.82414996623993
