# Supplementary material for: Investigation of Evolutionary History and Origin of the Tre1 Family Suggests a Role in Regulating Hemocytes Cells Infiltration of the Blood–Brain Barrier
Source: Insects. 2021 Sep 29;12(10):882. doi: 10.3390/insects12100882 (PMC8540695; doi:10.3390/insects12100882)
Supplement: Supplementary file 1 [file insects-12-00882-s001.zip › suppl_files_2_adera.zip!/DRSC RNA Seq Browser_moody_adera.pdf]

Integration of Unwounded, Wounded, and Wasp infested 24 hr

Project ID: 26 *Sudhir Tattikota*

Coordinate Ids

tSNE

Submit

Select Condition / Sample

- (selected) Wounded
- Unwounded
- Wasp infested 24 hr
- all

Submit

tSNE

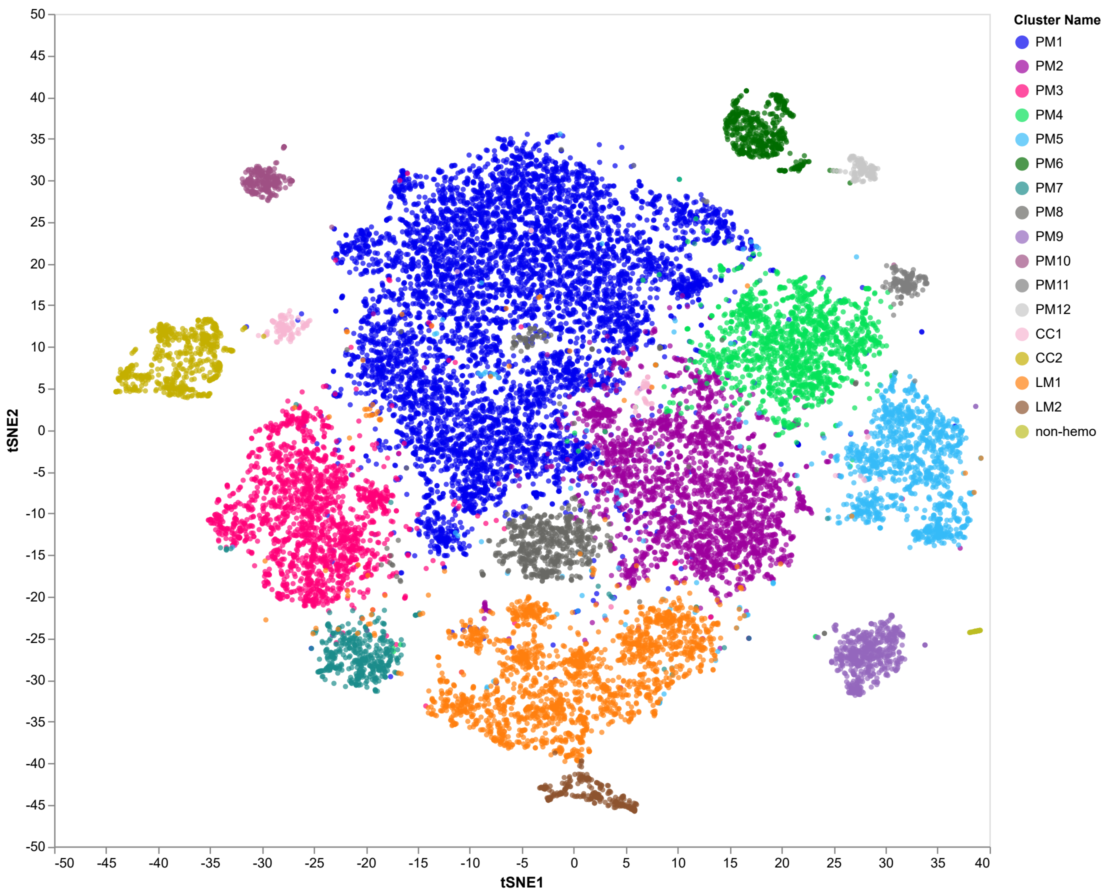

stack / unstack graphs

- Gene Lookup moody =>FBgn0025631 (https://www.flybase.org/reports/FBgn0025631)
- Genes In Results: FBgn0025631 (https://www.flybase.org/reports/FBgn0025631)

Bee Chart (/tools/rna_seq_sgt2/web/violin_chart/26/plot_coord=0/sample_id=all/moody)

Gene Names (up to 5 comma separated)

moody

Search

tSNE

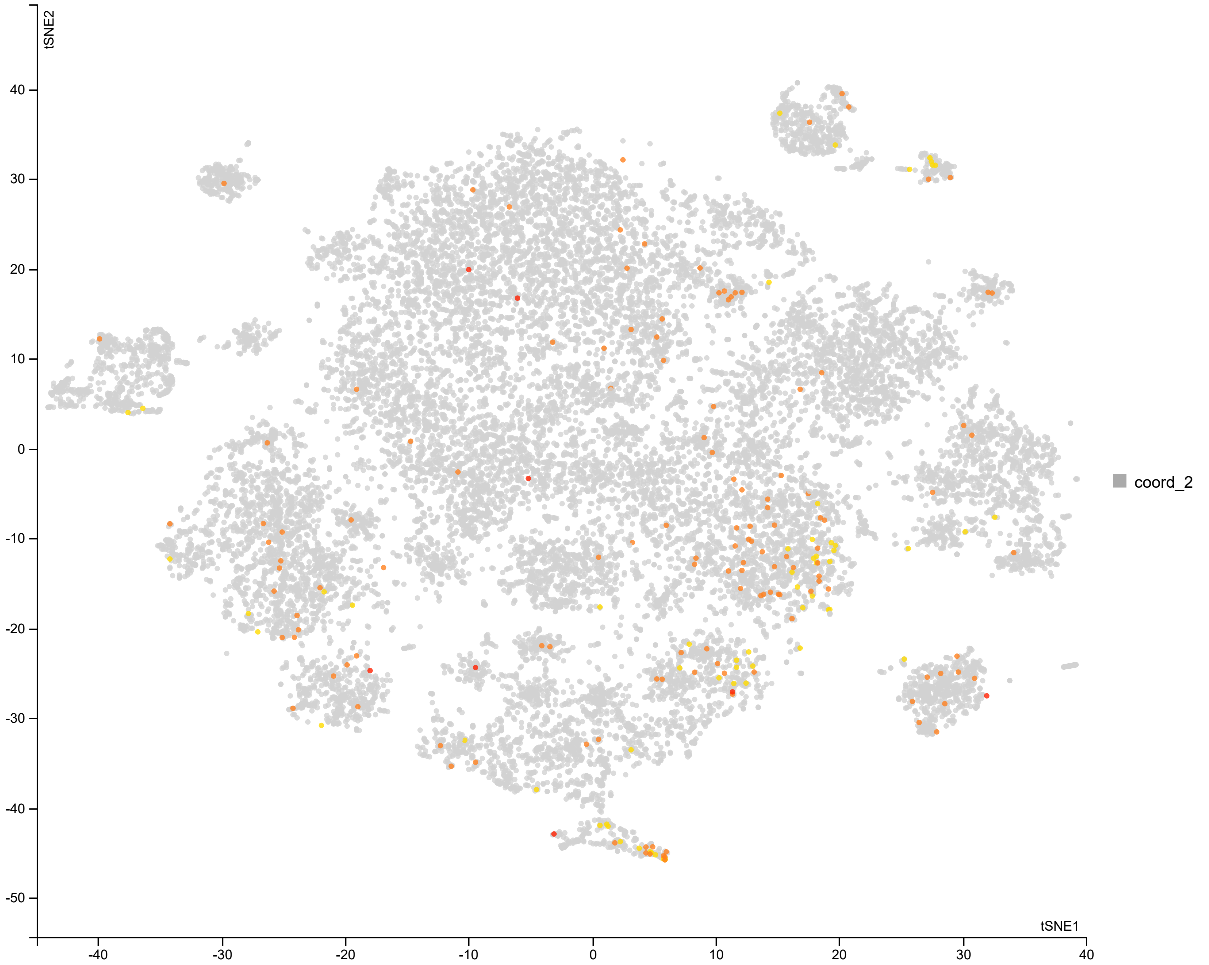

export graph (png)

2500 export width px

export graph (svg)

log normalized UMI:

<=0 0 1 2 4

Adjust:

0 -> 0

0 -> 1

1 -> 2

2 -> 4

4 -> 8

Reset Values

Summary of Cells Expressing Selected Genes

Gene	PM1	PM2	PM3	PM4	PM5	PM6	PM7	PM8	PM9	PM10	PM11	PM12	CC1	CC2	LM1	LM2	non-hemo
FBgn0025631	24 (0.4%)	58 (2.3%)	20 (1.1%)	3 (0.2%)	8 (0.7%)	5 (1%)	5 (1.2%)	4 (0.6%)	11 (2.1%)	1 (0.4%)	2 (1.4%)	7 (5.3%)	0	3 (0.5%)	31 (1.6%)	24 (8.8%)	0
Total Cluster Count	6796	2500	1810	1461	1204	500	421	630	512	243	148	131	154	643	1993	272	40

All Cell Count Summary

Sample Name	PM1	PM2	PM3	PM4	PM5	PM6	PM7	PM8	PM9	PM10	PM11	PM12	CC1	CC2	LM1	LM2	non-hemo
all	6796	2500	1810	1461	1204	500	421	630	512	243	148	131	154	643	1993	272	40
Unwounded	1546	604	315	377	288	3	44	141	171	86	47	0.	26	125	311	26	0.
Wasp infested 24 hr	1987	631	731	534	438	342	183	144	19	21	42	0.	36	162	735	125	40
Wounded	3263	1265	764	550	478	155	194	345	322	136	59	131	92	356	947	121	0.

Data Table

Cell Barcode	Cluster Name	FBgn0025631
injured_indrop_PC2_blood3_TCGCATAA.GTGTCAGG.CTACGAGC	PM1	1.12055003643036
injured_tenx_B2_GAGTCCGTCCGCGGTA	PM1	1.12097001075745
injured_indrop_PC2_blood3_TCGCATAA.GTCCTTGC.GTGTAACC	PM1	1.17515003681183
injured_indrop_PC2_blood3_TCGCATAA.GTAATCTG.GAGTTTCG	PM1	1.24955999851227
injured_indrop_PC2_blood3_TCGCATAA.TCGCAATC.TTCCGAGT	PM1	1.30219995975494
injured_tenx_B4_TATGCCCCAGGTCGTC	PM1	1.30611002445221
injured_indrop_PC2_blood3_TCGCATAA.ACCTTCCC.GCTCTAGT	PM1	1.35119998455048
injured_indrop_PC1_blood2_CTTAATAG.TAAGGGCC.CCCGACTT	PM1	1.38952994346619
injured_tenx_B2_AGACGTTGTAGCACGA	PM1	1.3938399553299
UNinjured_tenx_B1_GATGAGGAGAGGTAGA	PM1	1.49896001815796
injured_indrop_PC2_blood3_TCGCATAA.AAAGCCCG.CAATACTT	PM1	1.52937996387482
injured_indrop_PC1_blood2_CTTAATAG.CCACCTCT.GGTTTCTC	PM1	1.55009996891022
injured_tenx_B2_TACTTGTCACGAAACG	PM1	1.63953995704651
injured_indrop_PC1_blood2_CTTAATAG.GGGCATCA.GTTGTCAT	PM1	1.64898002147675
injured_indrop_PC2_blood3_TCGCATAA.CAACGCAG.GACGATGG	PM1	1.70703995227814
injured_tenx_B4_CCTAAAGCAGATGGGT	PM1	1.77121996879578
injured_indrop_PC2_blood3_TCGCATAA.CGATCCAA.GGGAACCT	PM1	1.85032999515533
injured_tenx_B2_CGCTGGAAGTACGACG	PM1	1.93298995494843
injured_tenx_B2_AGCTCTCCACAGATTC	PM1	1.97029995918274
injured_tenx_B4_ACAGCTAAGTCATCCA	PM1	1.97616994380951
injured_tenx_B2_ATGAGGGTCTTACCGC	PM1	1.99075996875763
injured_tenx_B2_ACAGCCGTCAGCACAT	PM1	2.04855990409851
injured_tenx_B2_CTCGTACGTATCACCA	PM1	2.18383002281189
UNinjured_indrop_UC2_blood3_TAAGGCTC_ACTCATAC_TCCCGTAG	PM1	2.430340051651
WASP96hr_dropseq_PI24CIRC2_TACTTGTATCCA.2	PM6	0.566197991371155
WASP96hr_dropseq_PI24CIRC2_TGATTATTTACG.2	PM6	0.726598024368286
injured_indrop_PC2_blood3_TCGCATAA.ACCACAGA.GCCGGATT	PM6	1.1835800409317
injured_indrop_PC2_blood3_TCGCATAA.TCAGCCTC.CCAACCGT	PM6	1.23675000667572
injured_indrop_PC2_blood3_TCGCATAA.CCGATACG.TACTTGTG	PM6	1.87282001972198
WASP96hr_dropseq_PI24CIRC2_ACATAGAACATG.2	PM7	0.383938997983932
injured_tenx_B4_ACATGGTTCAACCATG	PM7	1.04544997215271
injured_tenx_B2_CTAGAGTAGCACGCCT	PM7	1.05352997779846
injured_indrop_PC2_blood3_TCGCATAA.CCGATACG.TTATAGCC	PM7	1.45945000648499
injured_indrop_PC2_blood3_TCGCATAA.AAATTCCG.GTCAAAGA	PM7	1.81289994716644
WASP96hr_dropseq_PI24CIRC3_TGTTTGACGTCC.3	LM2	0.360619008541107
WASP96hr_dropseq_PI24CIRC3_ACCCTTTTTGAT.3	LM2	0.387340992689133
WASP96hr_dropseq_PI24CIRC3_GTCAGTAGAACT.3	LM2	0.445333003997803

Cell Barcode	Cluster Name	FBgn0025631
WASP96hr_dropseq_PI24CIRC2_ATGCAAAAGGTT.2	LM2	0.4774030148983
WASP96hr_dropseq_PI24CIRC3_ACCTGGGCAAAA.3	LM2	0.523177027702332
WASP96hr_dropseq_PI24CIRC1_GAGGATGTATGG.1	LM2	0.54238098859787
WASP96hr_dropseq_PI24CIRC3_TCTATTCCGTTC.3	LM2	0.614666998386383
WASP96hr_dropseq_PI24CIRC3_GGTCCTTGGTGT.3	LM2	0.655584990978241
WASP96hr_dropseq_PI24CIRC2_CGCGGTGTGTAT.2	LM2	0.736841022968292
injured_tenx_B2_CGCGTTTGTCGCTTCT	LM2	0.740092992782593
injured_tenx_B4_CTCCTAGGTAAATGAC	LM2	0.76385098695755
WASP96hr_dropseq_PI24CIRC3_GCCCCAAAGGAT.3	LM2	0.77980899810791
WASP96hr_dropseq_PI24CIRC3_GTAGGACGCGCT.3	LM2	0.880303978919983
WASP96hr_dropseq_PI24CIRC3_GGCGTGAATGGG.3	LM2	0.88696700334549
injured_tenx_B2_ATCGAGTTCATTTGGG	LM2	1.13295996189117
WASP96hr_dropseq_PI24CIRC1_GAGGATTATGGG.1	LM2	1.17717003822327
injured_tenx_B4_CAACCTCAGACACTAA	LM2	1.37766003608704
injured_tenx_B2_ATCATGGCATGACATC	LM2	1.44219994544983
injured_tenx_B2_ACTGCTCCATCACGTA	LM2	1.5436999797821
injured_indrop_PC1_blood2_CTTAATAG.GTGCCCAT.GGCCTAAG	LM2	1.56818997859955
WASP96hr_dropseq_PI24CIRC3_TTACATTTACCA.3	LM2	1.58463001251221
injured_tenx_B2_TTGGCAAGTTACCAGT	LM2	1.75978004932404
injured_indrop_PC1_blood2_CTTAATAG.AAACACTA.GCTCTAGT	LM2	1.92295002937317
WASP96hr_dropseq_PI24CIRC3_CGAAGCATTTCT.3	LM2	2.51372003555298
injured_indrop_PC1_blood2_CTTAATAG.AGTCACAA.GACAACTT	PM10	1.14216995239258
UNinjured_indrop_UC1_blood2_TAAGGCTC_ATCCCACC_CTTCGATT	PM11	1.32710003852844
injured_indrop_PC1_blood2_CTTAATAG.CGGCTGTA.CTCTTGAC	PM11	1.49680995941162
injured_tenx_B4_TAGTGGTTCATCGATG	PM12	0.562178015708923
injured_tenx_B4_TGTGGTACACAGGTTT	PM12	0.583692014217377
injured_tenx_B4_TTAGGCATCGCTAGCG	PM12	0.611603021621704
injured_tenx_B4_CATCGAAAGCCGGTAA	PM12	0.681070029735565
injured_tenx_B4_CTCGAGGAGAGCTGGT	PM12	0.687053024768829
injured_tenx_B4_AAAGTAGGTCTTCTCG	PM12	1.17887997627258
injured_tenx_B4_TCTATTGAGGAGTCTG	PM12	1.55508995056152
UNinjured_tenx_B3_TCTTCGGGTGTGAATA	PM2	0.564981997013092
injured_tenx_B4_TCTATTGCAGACGCTC	PM2	0.725441992282867
UNinjured_tenx_B3_GAGCAGATCTCTTGAT	PM2	0.726266980171204
injured_tenx_B2_ACGTCAACAAGACGTG	PM2	0.727205991744995
injured_tenx_B4_AGAATAGTCTGCCCTA	PM2	0.745930016040802
injured_tenx_B4_TGGCGCATCGCCCTTA	PM2	0.834921002388
injured_tenx_B4_ACTGATGAGGTGACCA	PM2	0.854834973812103
UNinjured_tenx_B3_CTCTACGCAACACGCC	PM2	0.870585978031158
injured_tenx_B4_CAGCATATCAGTGCAT	PM2	0.87212198972702
UNinjured_tenx_B3_GTTCATTTCAGAGACG	PM2	0.90218597650528
injured_tenx_B4_CAGCATACAGGAACGT	PM2	0.93530398607254
UNinjured_tenx_B3_CGTCTACCAAAGGTGC	PM2	0.944907009601593
injured_tenx_B4_CACATTTTCAACACGT	PM2	0.947027027606964
injured_tenx_B4_TGAGCATGTCTAAACC	PM2	0.949739992618561

Cell Barcode	Cluster Name	FBgn0025631
injured_tenx_B4_CATCAGAGTCCGAGTC	PM2	0.992541015148163
injured_tenx_B4_CGGACTGCAATCGGTT	PM2	0.999107003211975
UNinjured_tenx_B3_TGCCCATAGCTGATAA	PM2	1.00411999225616
UNinjured_tenx_B3_AAAGATGCAGATCGGA	PM2	1.00775003433228
UNinjured_tenx_B3_CCGTGGACATCTCCCA	PM2	1.02611005306244
injured_tenx_B4_TTAGGACGTAGCCTAT	PM2	1.03504002094269
UNinjured_indrop_UC1_blood2_TAAGGCTC_GGCTTTGC_GCCACATC	PM2	1.03737998008728
injured_tenx_B4_CGTTAGATCCTATTCA	PM2	1.04294002056122
UNinjured_indrop_UC1_blood2_TAAGGCTC_CCGGAAAT_AACAATCC	PM2	1.04544997215271
injured_tenx_B4_GTAGGCCTCATATCGG	PM2	1.06974005699158
injured_tenx_B2_CGGTTAAAGTTGAGTA	PM2	1.06985998153687
injured_tenx_B2_CCACGGAGTTCACCTC	PM2	1.07568001747131
UNinjured_tenx_B3_CATGCCTCAACAACCT	PM2	1.0986100435257
injured_indrop_PC2_blood3_TCGCATAA.TACCTCCC.GGCCTAAG	PM2	1.10531997680664
injured_tenx_B4_CGAACATAGCGAGAAA	PM2	1.115149974823
injured_tenx_B4_CAGCGACCACCGCTAG	PM2	1.12656998634338
injured_tenx_B4_CTTACCGGTGCGAAAC	PM2	1.12799000740051
UNinjured_indrop_UC1_blood2_TAAGGCTC_GGTAGCCA_GTAACGTT	PM2	1.15974998474121
injured_tenx_B2_AACTCTTCAGCTCGAC	PM2	1.16823995113373
injured_tenx_B4_GCTCTGTAGTGTCTCA	PM2	1.17253005504608
injured_tenx_B4_TTGGCAAGTCGGCATC	PM2	1.17997002601624
injured_tenx_B4_TAAACCGGTCTGCCAG	PM2	1.18436002731323
injured_indrop_PC2_blood3_TCGCATAA.CGACATTT.CGCTAGTC	PM2	1.18973994255066
UNinjured_indrop_UC1_blood2_TAAGGCTC_CATTCATT_GTTGCACG	PM2	1.19037997722626
injured_tenx_B4_CATCAAGTCAGAGACG	PM2	1.19102001190186
injured_indrop_PC2_blood3_TCGCATAA.ACTAGAGC.ACGGTAGC	PM2	1.19117999076843
injured_tenx_B2_CCGTTCATCCTGCCAT	PM2	1.19245004653931
injured_tenx_B4_ATAGACCAGCGTCAAG	PM2	1.20115995407104
injured_tenx_B4_ACAGCTAGTAGCGTCC	PM2	1.20246005058289
injured_tenx_B4_GATTCAGGTACTCGCG	PM2	1.21967005729675
injured_indrop_PC1_blood2_CTTAATAG.GTCAGACC.ATCCCACG	PM2	1.24742996692657
injured_tenx_B4_AACTTTCGTAGCGATG	PM2	1.26375997066498
injured_indrop_PC1_blood2_CTTAATAG.ACTACCCG.CTCAGAAT	PM2	1.27684998512268
injured_tenx_B4_CCTCTGACAGACTCGC	PM2	1.29560995101929
injured_indrop_PC1_blood2_CTTAATAG.AGAAAGTG.TGTGCTCA	PM2	1.32627999782562
injured_tenx_B4_GATCGCGAGTAGGTGC	PM2	1.33509004116058
injured_indrop_PC2_blood3_TCGCATAA.AAGAAGGT.CGGACAAC	PM2	1.35396003723145
injured_indrop_PC1_blood2_CTTAATAG.TAACTTCT.TATTGCCT	PM2	1.36928999423981
UNinjured_tenx_B3_GGCGTGTCAAGGCTCC	PM2	1.37191998958588
UNinjured_indrop_UC1_blood2_TAAGGCTC_AGAGGTGC_AGCGAAGT	PM2	1.40954005718231
UNinjured_indrop_UC1_blood2_TAAGGCTC_ACCAGTTT_ATCAGCGC	PM2	1.44046998023987
UNinjured_indrop_UC1_blood2_TAAGGCTC_AAAGCCCG_TTCACATA	PM2	1.47355997562408
injured_indrop_PC2_blood3_TCGCATAA.TGAATAGG.TTAAACTG	PM2	1.48958003520966
injured_indrop_PC2_blood3_TCGCATAA.GATGTGGC.ACGCAGAG	PM2	1.70151996612549
WASP96hr_dropseq_PI24CIRC2_AGGAATTTACTC.2	LM1	0.351547986268997

Cell Barcode	Cluster Name	FBgn0025631
UNinjured_tenx_B3_AAATGCCTCGTTGCCT	LM1	0.37473601102829
WASP96hr_dropseq_PI24CIRC2_CGGTCCCCCAAT.2	LM1	0.536125004291534
WASP96hr_dropseq_PI24CIRC1_GGTTGAACAAGT.1	LM1	0.540085017681122
WASP96hr_dropseq_PI24CIRC2_ATACTCTCCGGC.2	LM1	0.607502996921539
injured_tenx_B2_CATCGAAAGGACGAAA	LM1	0.614784002304077
injured_tenx_B2_CGTAGCGTCAGAGGTG	LM1	0.660025000572205
WASP96hr_dropseq_PI24CIRC3_TGAGCTAGGTCT.3	LM1	0.791172981262207
injured_tenx_B4_CAGGTGCTCTTTAGTC	LM1	0.82956600189209
injured_tenx_B4_GGCTGGTAGTCTCCTC	LM1	0.934364020824432
injured_tenx_B2_CTCAGAAGTATAAACG	LM1	0.946062028408051
injured_indrop_PC1_blood2_CTTAATAG.GAAGATAT.CTAGATTG	LM1	0.973801016807556
WASP96hr_dropseq_PI24CIRC2_CGTCATCGTCGG.2	LM1	1.02175998687744
injured_indrop_PC2_blood3_TCGCATAA.CTGTCGCA.CCTCGTCT	LM1	1.06262004375458
injured_tenx_B2_TTTGTCAAGGTGCTAG	LM1	1.09475994110107
injured_tenx_B4_ACTATCTCATTAACCG	LM1	1.10898995399475
UNinjured_tenx_B3_AGCTCTCTCAGGCGAA	LM1	1.11281001567841
injured_indrop_PC1_blood2_CTTAATAG.GGCGACAA.GACAACTT	LM1	1.15661001205444
injured_indrop_PC1_blood2_CTTAATAG.ACTCGGAA.GAAGTGCC	LM1	1.16200995445251
injured_indrop_PC1_blood2_CTTAATAG.AGAGAGAC.CATCGCAG	LM1	1.17949998378754
injured_tenx_B2_GTGTTAGCACCTGGTG	LM1	1.21530997753143
WASP96hr_dropseq_PI24CIRC2_GATCTTACCACC.2	LM1	1.22457003593445
UNinjured_tenx_B1_CTAATGGTCGCCCTTA	LM1	1.24636995792389
injured_indrop_PC1_blood2_CTTAATAG.AAACTACA.GCCGGATT	LM1	1.25868999958038
injured_tenx_B4_GAGTCCGCATTCCTGC	LM1	1.33364999294281
injured_tenx_B4_TGGTTCCCACCGCTAG	LM1	1.48705995082855
injured_indrop_PC2_blood3_TCGCATAA.ATTGAAGT.AAAGTCGG	LM1	1.63654005527496
injured_indrop_PC2_blood3_TCGCATAA.GCTAAGTT.ACGTATAC	LM1	1.65204000473022
injured_tenx_B2_GTGGGTCGTGCCTGTG	LM1	1.89552998542786
injured_indrop_PC1_blood2_CTTAATAG.TACCTAAG.GTTTACGT	LM1	2.0644199848175
UNinjured_tenx_B1_TAAACCGAGCCTCGTG	LM1	2.23135995864868
WASP96hr_dropseq_PI24CIRC2_CGATGCGACATT.2	PM3	0.489948004484177
WASP96hr_dropseq_PI24CIRC2_ATTCATATCAGC.2	PM3	0.648711979389191
WASP96hr_dropseq_PI24CIRC2_GTATATGTCGTG.2	PM3	0.695002019405365
WASP96hr_dropseq_PI24CIRC2_GTTCTTGTCTGA.2	PM3	0.862990975379944
UNinjured_tenx_B3_TGCGGGTAGGGTTTCT	PM3	0.890169978141785
injured_tenx_B2_GATCGTAGTAAGGGAA	PM3	1.02830004692078
injured_tenx_B4_TACCTTAGTCTCCACT	PM3	1.18154001235962
injured_tenx_B2_TGACAACAGTCACGCC	PM3	1.19357001781464
injured_indrop_PC1_blood2_CTTAATAG.TAGCCACA.CTCGCGTA	PM3	1.21865999698639
injured_indrop_PC1_blood2_CTTAATAG.ATGGGCTC.AGTGAAAG	PM3	1.30748999118805
injured_indrop_PC1_blood2_CTTAATAG.TAGCGGAT.CGTGCTAG	PM3	1.32202994823456
injured_tenx_B2_GTCTTCGAGAGTCGGT	PM3	1.4586900472641
injured_tenx_B2_GACTAACAGAGTCGGT	PM3	1.54051995277405
injured_tenx_B2_TTGTAGGTCAATCACG	PM3	1.58431994915009
injured_tenx_B2_GGGACCTAGGTGATAT	PM3	1.62858998775482

Cell Barcode	Cluster Name	FBgn0025631
injured_indrop_PC2_blood3_TCGCATAA.TCGTTCCC.AAGATTGT	PM3	1.65102005004883
injured_tenx_B4_CAACCTCAGGCGCTCT	PM3	1.67000997066498
injured_indrop_PC2_blood3_TCGCATAA.AGCGTGGT.GGTGACAC	PM3	1.68487000465393
injured_indrop_PC2_blood3_TCGCATAA.GCCAACAT.GGGAAGGT	PM3	1.78677999973297
injured_indrop_PC2_blood3_TCGCATAA.GTGAACTC.TAGTCGCA	PM3	2.02995991706848
WASP96hr_dropseq_PI24CIRC3_TCTGTCACTATC.3	PM4	0.986277997493744
injured_tenx_B4_CATCAAGTCAAACCAC	PM4	1.46915996074677
injured_tenx_B4_GACTAACCATACGCCG	PM4	1.62170994281769
injured_tenx_B4_CCTTCGAAGTTAGGTA	PM5	0.632950007915497
WASP96hr_dropseq_PI24CIRC2_CACGTTACATCT.2	PM5	0.787238001823425
injured_tenx_B4_GCATACAGTAACGACG	PM5	0.85097199678421
UNinjured_indrop_UC1_blood2_TAAGGCTC_TCGGTCAT_CTACGAGC	PM5	0.964260995388031
WASP96hr_dropseq_PI24CIRC3_GAGTTCGAGGAC.3	PM5	1.24566996097565
UNinjured_tenx_B3_GGACAGACATGGTAGG	PM5	1.28303003311157
UNinjured_tenx_B3_AACGTTGCACAGCCCA	PM5	1.38568997383118
injured_indrop_PC1_blood2_CTTAATAG.TGACGTCG.GTCAAAGA	PM5	1.40049004554749
injured_tenx_B4_CCGTACTAGAGTACAT	CC2	0.490635007619858
injured_tenx_B2_GATGAAAGTGCGCTTG	CC2	0.589569985866547
injured_indrop_PC1_blood2_CTTAATAG.GTAAGCCG.CACAACAG	CC2	1.43948996067047
UNinjured_tenx_B3_CCGGTAGCATGCCTAA	PM8	0.88187700510025
WASP96hr_dropseq_PI24CIRC2_TAACTATGGGTG.2	PM8	1.01296997070312
UNinjured_tenx_B3_CGGGTCAAGTTAGGTA	PM8	1.16351997852325
injured_tenx_B4_GTAACTGAGCTAGTCT	PM8	1.65033996105194
WASP96hr_dropseq_PI24CIRC1_GATGCCGGGATG.1	PM9	0.561739981174469
injured_indrop_PC1_blood2_CTTAATAG.ACACCAAG.ATGCATGG	PM9	1.29272997379303
UNinjured_indrop_UC1_blood2_TAAGGCTC_CATTCATT_CCCATCTG	PM9	1.3027800321579
injured_tenx_B2_TCGGTAATCCTACAGA	PM9	1.35781002044678
injured_indrop_PC2_blood3_TCGCATAA.CTGTTAAA.AGTGAAAG	PM9	1.3952100276947
injured_indrop_PC2_blood3_TCGCATAA.ACAAGTAG.TTGTCGCC	PM9	1.41729998588562
injured_tenx_B2_GGGATGAGTCACCCAG	PM9	1.4805999994278
injured_tenx_B4_GTGCATACAAGCCATT	PM9	1.50683999061584
injured_tenx_B2_GGCCGATGTCCGCTGA	PM9	1.6243200302124
UNinjured_indrop_UC1_blood2_TAAGGCTC_GTCTGGAA_AAAGCCTA	PM9	1.71559000015259
WASP96hr_dropseq_PI24CIRC3_ATGGGGGGTTAG.3	PM9	2.04798007011414
