# Supplementary material for: Investigation of Evolutionary History and Origin of the Tre1 Family Suggests a Role in Regulating Hemocytes Cells Infiltration of the Blood–Brain Barrier
Source: Insects. 2021 Sep 29;12(10):882. doi: 10.3390/insects12100882 (PMC8540695; doi:10.3390/insects12100882)
Supplement: Supplementary file 1 [file insects-12-00882-s001.zip › suppl_files_2_adera.zip!/DRSC RNA Seq Browser_Tre1_adera.pdf]

Integration of Unwounded, Wounded, and Wasp infested 24 hr

Project ID: 26 *Sudhir Tattikota*

Coordinate Ids

tSNE

Submit

Select Condition / Sample

- (selected) Wounded
- Unwounded
- Wasp infested 24 hr
- all

Submit

tSNE

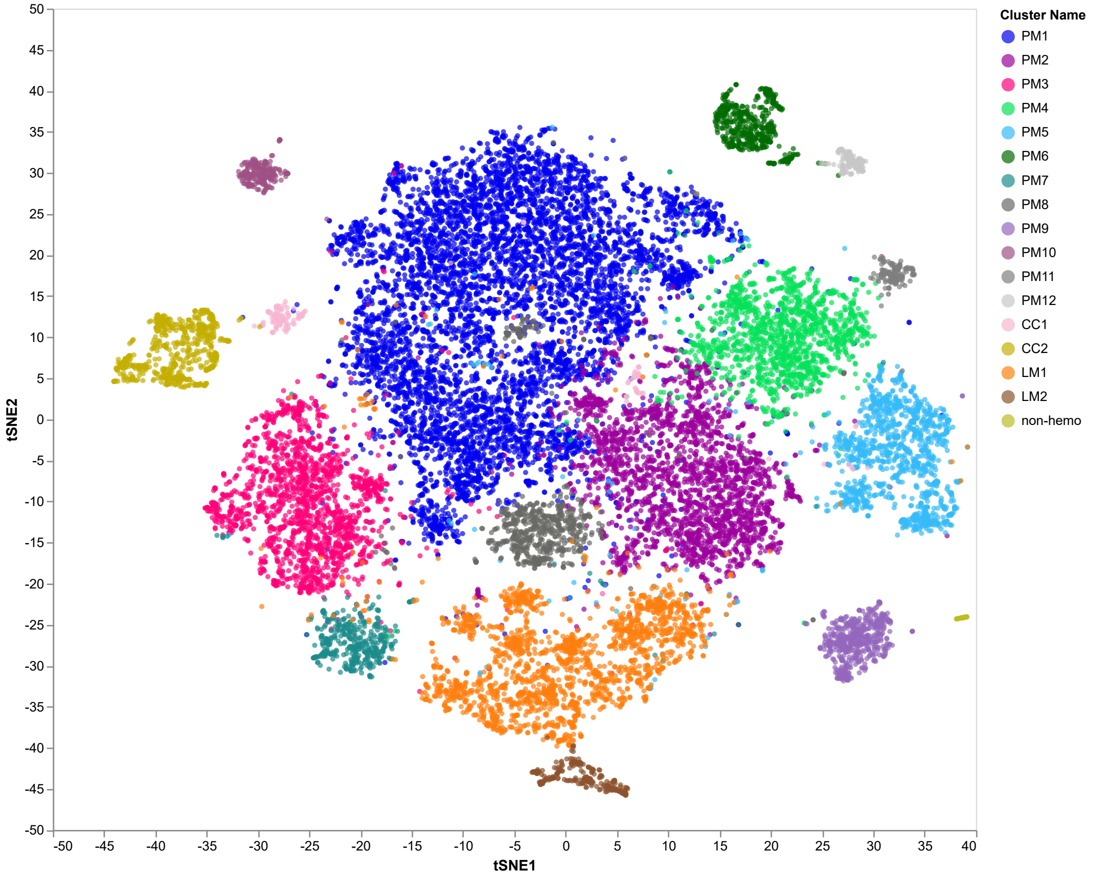

stack / unstack graphs

- Gene Lookup tre1 =>FBgn0046687 (https://www.flybase.org/reports/FBgn0046687)
- Genes In Results: FBgn0046687 (https://www.flybase.org/reports/FBgn0046687)

Bee Chart (/tools/rna_seq_sgt2/web/violin_chart/26/plot_coord=0/sample_id=all/tre1)

Gene Names (up to 5 comma separated)

tre1

Search

tSNE

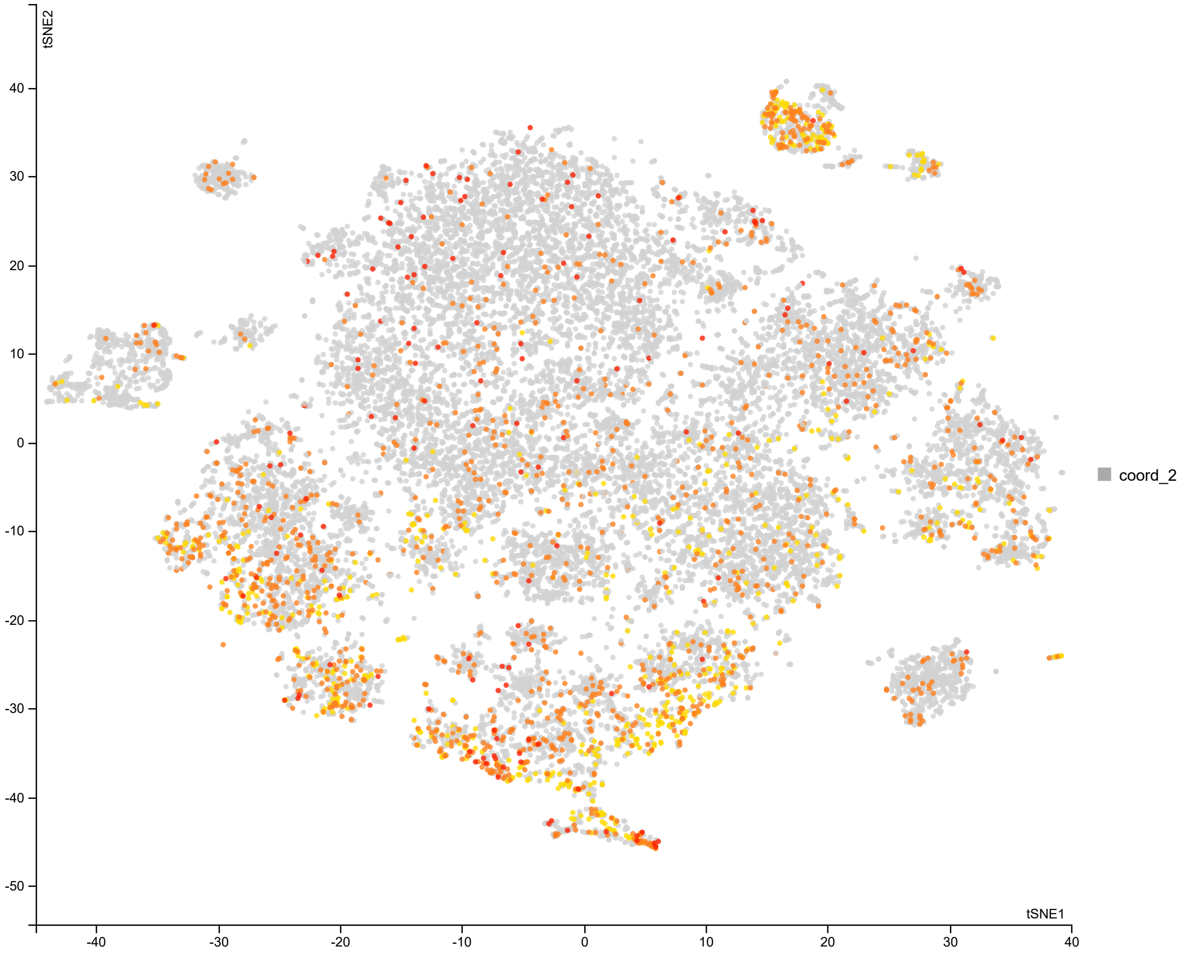

export graph (png) | 2500 | export width px

export graph (svg)

log normalized UMI:

<=0 0 1 2 4

Adjust:

0 -> 0

0 -> 1

1 -> 2

2 -> 4

4 -> 8

Reset Values

Summary of Cells Expressing Selected Genes

Gene	PM1	PM2	PM3	PM4	PM5	PM6	PM7	PM8	PM9	PM10	PM11	PM12	CC1	CC2	LM1	LM2	non- hem
FBgn0046687	328 (4.8%)	197 (7.9%)	325 (18%)	103 (7%)	123 (10.2%)	170 (34%)	130 (30.9%)	44 (7%)	35 (6.8%)	13 (5.3%)	13 (8.8%)	18 (13.7%)	5 (3.2%)	31 (4.8%)	506 (25.4%)	94 (34.6%)	8 (20%
Total Cluster Count	6796	2500	1810	1461	1204	500	421	630	512	243	148	131	154	643	1993	272	40

All Cell Count Summary

Sample Name	PM1	PM2	PM3	PM4	PM5	PM6	PM7	PM8	PM9	PM10	PM11	PM12	CC1	CC2	LM1	LM2	non-hemo
all	6796	2500	1810	1461	1204	500	421	630	512	243	148	131	154	643	1993	272	40
Unwounded	1546	604	315	377	288	3	44	141	171	86	47	0.	26	125	311	26	0.
Wasp infested 24 hr	1987	631	731	534	438	342	183	144	19	21	42	0.	36	162	735	125	40
Wounded	3263	1265	764	550	478	155	194	345	322	136	59	131	92	356	947	121	0.

Data Table

Cell Barcode	Cluster Name	FBgn0046687
WASP96hr_dropseq_PI24CIRC1_TGTCCGGGACTG.1	PM1	0.309073001146317
WASP96hr_dropseq_PI24CIRC1_AGACTAGAAAGA.1	PM1	0.32422998547554
WASP96hr_dropseq_PI24CIRC1_CATACAGCAAAA.1	PM1	0.380948007106781
WASP96hr_dropseq_PI24CIRC2_GATCATTCAGAG.2	PM1	0.523905992507935
WASP96hr_dropseq_PI24CIRC2_CCCCGGCAGTAT.2	PM1	0.555859982967377
WASP96hr_dropseq_PI24CIRC3_TGCCGAATTCTG.3	PM1	0.591940999031067
WASP96hr_dropseq_PI24CIRC2_GCTAGTACTGCC.2	PM1	0.602410018444061
WASP96hr_dropseq_PI24CIRC2_GACGATGTGGAT.2	PM1	0.604474008083344
WASP96hr_dropseq_PI24CIRC1_GCAATCCGGTGA.1	PM1	0.611833989620209
WASP96hr_dropseq_PI24CIRC1_GCAATCCGGTGG.1	PM1	0.676903009414673
injured_tenx_B4_AGGTCATGTTGAACTC	PM1	0.696614980697632
WASP96hr_dropseq_PI24CIRC1_CGACATAATGTT.1	PM1	0.698629021644592
WASP96hr_dropseq_PI24CIRC3_AACTGCTACAGT.3	PM1	0.708963990211487
WASP96hr_dropseq_PI24CIRC3_GGCCCCATGCCA.3	PM1	0.716005980968475
WASP96hr_dropseq_PI24CIRC3_GCGCAGCATTTC.3	PM1	0.7223219871521
WASP96hr_dropseq_PI24CIRC2_CGGCCACACACT.2	PM1	0.733281016349792
WASP96hr_dropseq_PI24CIRC3_CCGGCGGCGTTG.3	PM1	0.745755016803741
WASP96hr_dropseq_PI24CIRC1_GACTGGAAAACA.1	PM1	0.754978001117706
WASP96hr_dropseq_PI24CIRC2_AGAGATAGACTG.2	PM1	0.7571160197258
WASP96hr_dropseq_PI24CIRC3_GACCCAGAACCG.3	PM1	0.78730297088623
WASP96hr_dropseq_PI24CIRC2_GACCGCGAGCCT.2	PM1	0.802618026733398
WASP96hr_dropseq_PI24CIRC3_CTAGATCCCTTT.3	PM1	0.803638994693756
WASP96hr_dropseq_PI24CIRC2_AGCTATCGTACA.2	PM1	0.820065021514893
WASP96hr_dropseq_PI24CIRC3_GACTAGACGAGG.3	PM1	0.820563018321991
WASP96hr_dropseq_PI24CIRC3_AATACTCCACTC.3	PM1	0.825940012931824
WASP96hr_dropseq_PI24CIRC2_AGCTGAGTCTAT.2	PM1	0.82949298620224
injured_tenx_B4_CTCGTCAGTCACTTCC	PM1	0.843204975128174
WASP96hr_dropseq_PI24CIRC3_AACTGTTTCTAT.3	PM1	0.848748028278351
WASP96hr_dropseq_PI24CIRC3_CGCTTGTATTTC.3	PM1	0.859755992889404
WASP96hr_dropseq_PI24CIRC3_ACAACGGGTTAA.3	PM1	0.870989978313446
WASP96hr_dropseq_PI24CIRC2_TACCCCTCAGCT.2	PM1	0.893185019493103
WASP96hr_dropseq_PI24CIRC3_ACGGTCGCGCTC.3	PM1	0.896995007991791
WASP96hr_dropseq_PI24CIRC2_CTGGCGACACAG.2	PM1	0.898717999458313
WASP96hr_dropseq_PI24CIRC3_GCGTTCCTGCCA.3	PM1	0.901925027370453
injured_tenx_B4_TTTCCTCTCGCAAGCC	PM1	0.904018998146057
WASP96hr_dropseq_PI24CIRC3_CAGTTTAGTTAC.3	PM1	0.913748025894165
WASP96hr_dropseq_PI24CIRC2_CCACTCTTTGTT.2	PM1	0.933098971843719
WASP96hr_dropseq_PI24CIRC3_GCGACTTCCCAT.3	PM1	0.952078998088837

Cell Barcode	Cluster Name	FBgn0046687
WASP96hr_dropseq_PI24CIRC2_ATATATAACATG.2	PM1	0.967688024044037
WASP96hr_dropseq_PI24CIRC3_ATTACTGCCCCT.3	PM1	0.972163021564484
WASP96hr_dropseq_PI24CIRC3_ATCGACTCATTC.3	PM1	0.977508008480072
WASP96hr_dropseq_PI24CIRC3_CCGAAGAGCTTG.3	PM1	0.985119998455048
WASP96hr_dropseq_PI24CIRC2_TTATTTAACGTT.2	PM1	0.990195989608765
WASP96hr_dropseq_PI24CIRC3_GGGGAGTGTAGG.3	PM1	0.997591018676758
WASP96hr_dropseq_PI24CIRC3_TTGCACGAAGGT.3	PM1	1.00908005237579
WASP96hr_dropseq_PI24CIRC3_TTGCCTGCGGTG.3	PM1	1.00918996334076
WASP96hr_dropseq_PI24CIRC2_AGGTGAGGAGAG.2	PM1	1.00973999500275
WASP96hr_dropseq_PI24CIRC3_ACTTACATGCGT.3	PM1	1.01353001594543
injured_tenx_B4_AAGGCAGGTTATCACG	PM1	1.01374995708466
injured_tenx_B4_ACGATACTCTGCTTGC	PM1	1.01961004734039
WASP96hr_dropseq_PI24CIRC3_ACCTCTATATCG.3	PM1	1.03154003620148
WASP96hr_dropseq_PI24CIRC3_AGCGTGAATATA.3	PM1	1.05463004112244
WASP96hr_dropseq_PI24CIRC3_GCAATATTGGGG.3	PM1	1.05524003505707
WASP96hr_dropseq_PI24CIRC3_TCCAAATATGGC.3	PM1	1.06052005290985
WASP96hr_dropseq_PI24CIRC3_GAACCCCACGTA.3	PM1	1.06262004375458
WASP96hr_dropseq_PI24CIRC3_TGGCTGTTTTAC.3	PM1	1.07440996170044
WASP96hr_dropseq_PI24CIRC3_ACGCCAGGGCAC.3	PM1	1.0760600566864
WASP96hr_dropseq_PI24CIRC3_TAGCAACTAAAT.3	PM1	1.0791300535202
injured_tenx_B4_GGATGTTCATGCCTAA	PM1	1.08247995376587
WASP96hr_dropseq_PI24CIRC3_CGCGTTGGAATC.3	PM1	1.08261001110077
WASP96hr_dropseq_PI24CIRC3_AACCTTAAACGC.3	PM1	1.083899974823
WASP96hr_dropseq_PI24CIRC3_TCCCCCTTCCAG.3	PM1	1.08949995040894
injured_tenx_B4_AGGTCCGTCCCTCAGT	PM1	1.09569001197815
WASP96hr_dropseq_PI24CIRC2_TGTATGTTCGGG.2	PM1	1.10952997207642
WASP96hr_dropseq_PI24CIRC3_AATTGGACCCTA.3	PM1	1.12040996551514
WASP96hr_dropseq_PI24CIRC2_CTACCGAAGAGC.2	PM1	1.13060998916626
WASP96hr_dropseq_PI24CIRC3_ACCGCTCGCTCC.3	PM1	1.13082003593445
WASP96hr_dropseq_PI24CIRC2_GAGTTCTGTGGA.2	PM1	1.13138997554779
WASP96hr_dropseq_PI24CIRC2_TGTTGGCAGTAG.2	PM1	1.13926994800568
WASP96hr_dropseq_PI24CIRC3_CGAAGACCTGCC.3	PM1	1.15407001972198
WASP96hr_dropseq_PI24CIRC3_GTGCTGCAATGG.3	PM1	1.16383004188538
UNinjured_indrop_UC1_blood2_TAAGGCTC_GACTAGCG_CATCGCAG	PM1	1.17499995231628
UNinjured_tenx_B3_TCACGAATCCAGATCA	PM1	1.17841005325317
WASP96hr_dropseq_PI24CIRC3_GGGTCGCCGCCT.3	PM1	1.18546998500824
WASP96hr_dropseq_PI24CIRC3_TAACCTAAACAG.3	PM1	1.18578004837036
WASP96hr_dropseq_PI24CIRC3_CCGGTGGCAGCG.3	PM1	1.18625998497009
WASP96hr_dropseq_PI24CIRC2_CGCACGCGGGGG.2	PM1	1.19855999946594
WASP96hr_dropseq_PI24CIRC3_GAACTCGCCCTA.3	PM1	1.19937002658844
injured_tenx_B2_GGCAATTCATTGCGGC	PM1	1.20686995983124
WASP96hr_dropseq_PI24CIRC3_GACATGACGATG.3	PM1	1.21555995941162
WASP96hr_dropseq_PI24CIRC3_CACGCGCGAAGC.3	PM1	1.21682000160217
WASP96hr_dropseq_PI24CIRC3_ATAGGCGTACTA.3	PM1	1.22669005393982
injured_tenx_B4_ACCGTAATCAACCATG	PM1	1.22830998897552
WASP96hr_dropseq_PI24CIRC3_GTGCCGCCGAGT.3	PM1	1.23536002635956

Cell Barcode	Cluster Name	FBgn0046687
injured_tenx_B4_AGGTCATTCTCTGCTG	PM1	1.24848997592926
UNinjured_indrop_UC1_blood2_TAAGGCTC_CCTTTACA_CCCGACTT	PM1	1.26175999641418
WASP96hr_dropseq_PI24CIRC3_ATGTACTTCGAT.3	PM1	1.26213002204895
WASP96hr_dropseq_PI24CIRC3_AAGGGTTTTCCA.3	PM1	1.26285004615784
WASP96hr_dropseq_PI24CIRC3_GCCATTACCAGG.3	PM1	1.26558995246887
WASP96hr_dropseq_PI24CIRC3_GGGCCCGGCATT.3	PM1	1.26925003528595
WASP96hr_dropseq_PI24CIRC3_CAAAGTAACCCG.3	PM1	1.2733199596405
WASP96hr_dropseq_PI24CIRC3_CGTTACTGTACG.3	PM1	1.27553999423981
WASP96hr_dropseq_PI24CIRC3_GCGGCTCTAGAT.3	PM1	1.28910005092621
WASP96hr_dropseq_PI24CIRC3_ATCCTGAAAGCG.3	PM1	1.29812002182007
WASP96hr_dropseq_PI24CIRC3_ATGAACCTCTCG.3	PM1	1.2989000082016
injured_indrop_PC2_blood3_TCGCATAA.ATAGATGT.GATCTCGG	PM1	1.30025005340576
WASP96hr_dropseq_PI24CIRC3_AGTAGTTTGAGA.3	PM1	1.30181002616882
WASP96hr_dropseq_PI24CIRC3_ATATCACTACTG.3	PM1	1.31421995162964
injured_indrop_PC2_blood3_TCGCATAA.ACAGGCCA.TGGTATGA	PM1	1.32790994644165
UNinjured_tenx_B3_ACCCACTTCTCGATGA	PM1	1.33179998397827
WASP96hr_dropseq_PI24CIRC3_ATGGGCGCAGGG.3	PM1	1.33447003364563
WASP96hr_dropseq_PI24CIRC3_GGCTCTAATTTG.3	PM1	1.33591997623444
injured_tenx_B4_CACCTTGAGGCCATAG	PM1	1.33674001693726
UNinjured_tenx_B1_GGGCACTTCTGGGCCA	PM1	1.3377799987793
UNinjured_tenx_B3_TTCTTAGCAATTGCTG	PM1	1.34151995182037
injured_tenx_B4_TCTGAGATCGGACAAG	PM1	1.34381997585297
WASP96hr_dropseq_PI24CIRC3_ATGGGCAGCCTA.3	PM1	1.35652005672455
WASP96hr_dropseq_PI24CIRC3_TCCGCTTCCCGG.3	PM1	1.36266005039215
injured_tenx_B2_ACTGCTCAGAGTGAGA	PM1	1.36406004428864
WASP96hr_dropseq_PI24CIRC3_GGGGAGCCGGTC.3	PM1	1.365149974823
WASP96hr_dropseq_PI24CIRC3_GCTAACTTTAAC.3	PM1	1.36580002307892
injured_indrop_PC2_blood3_TCGCATAA.ATTTCCAT.TTGGCGTT	PM1	1.38659000396729
WASP96hr_dropseq_PI24CIRC3_CATCGCTTAGTG.3	PM1	1.38884997367859
WASP96hr_dropseq_PI24CIRC3_ATGCTGACAGAC.3	PM1	1.39293003082275
injured_tenx_B4_CCCAATCAGGCTAGGT	PM1	1.39293003082275
UNinjured_indrop_UC1_blood2_TAAGGCTC_AAAGAGGC_CGACGTCA	PM1	1.40325999259949
UNinjured_indrop_UC1_blood2_TAAGGCTC_TGCTTGGG_AGTGAAAG	PM1	1.40813994407654
WASP96hr_dropseq_PI24CIRC3_TTTGATTCTGGC.3	PM1	1.42110002040863
injured_indrop_PC1_blood2_CTTAATAG.CTCAAACA.CTACTTGT	PM1	1.42157995700836
injured_tenx_B4_TGCGTGGAGAGCCCAA	PM1	1.42264997959137
WASP96hr_dropseq_PI24CIRC3_CTCCTTATGCGA.3	PM1	1.4297399520874
WASP96hr_dropseq_PI24CIRC3_CCTACACTTGGG.3	PM1	1.43753004074097
injured_indrop_PC1_blood2_CTTAATAG.ATTACCTT.TGTGGCTA	PM1	1.43948996067047
WASP96hr_dropseq_PI24CIRC3_GCGAAGTTGGAT.3	PM1	1.44690001010895
WASP96hr_dropseq_PI24CIRC3_GTGCGCCTCAGT.3	PM1	1.45238995552063
injured_indrop_PC1_blood2_CTTAATAG.CGGACTAA.AATGTTTG	PM1	1.45895004272461
WASP96hr_dropseq_PI24CIRC2_CCGATTTAAGCA.2	PM1	1.45945000648499
WASP96hr_dropseq_PI24CIRC2_CGCACTACTTGG.2	PM1	1.46607995033264
WASP96hr_dropseq_PI24CIRC3_CTGTAAGTATTC.3	PM1	1.46852004528046
injured_indrop_PC1_blood2_CTTAATAG.AGTAGTTA.CAATACTT	PM1	1.47616004943848

Cell Barcode	Cluster Name	FBgn0046687
injured_tenx_B2_CACATTTCAGCCTATA	PM1	1.47851002216339
WASP96hr_dropseq_PI24CIRC3_GTATTGGCTGGG.3	PM1	1.48269999027252
injured_indrop_PC2_blood3_TCGCATAA.TACGTTCG.ATGGGCAC	PM1	1.48322999477386
WASP96hr_dropseq_PI24CIRC3_GTGGATTTGTGC.3	PM1	1.49626994132996
WASP96hr_dropseq_PI24CIRC3_CTGGTCCGAACT.3	PM1	1.50411999225616
WASP96hr_dropseq_PI24CIRC3_GTCGTCTAGCGC.3	PM1	1.50521004199982
injured_indrop_PC2_blood3_TCGCATAA.CTCCCAAA.AGTGATGC	PM1	1.51040995121002
injured_tenx_B2_CCGGGATAGGCAAAGA	PM1	1.51095998287201
injured_indrop_PC2_blood3_TCGCATAA.GTACGCTT.CGAGTCTG	PM1	1.51344001293182
WASP96hr_dropseq_PI24CIRC3_GCAATATTGGGC.3	PM1	1.51538002490997
WASP96hr_dropseq_PI24CIRC3_TTCTAGCGCGCC.3	PM1	1.51689994335175
WASP96hr_dropseq_PI24CIRC2_TTGGCAACGACG.2	PM1	1.52112996578217
WASP96hr_dropseq_PI24CIRC3_TCGAGAGAAGTG.3	PM1	1.52206003665924
injured_tenx_B4_GCGCAGTTCGAATGGG	PM1	1.52769005298615
WASP96hr_dropseq_PI24CIRC3_GCTAGGATGCCA.3	PM1	1.52824997901917
WASP96hr_dropseq_PI24CIRC3_ATGAACCTCTCC.3	PM1	1.53336000442505
injured_indrop_PC2_blood3_TCGCATAA.GTACGCTT.CAATTAGT	PM1	1.54340994358063
UNinjured_indrop_UC1_blood2_TAAGGCTC_GGGCATCA_TAACCCGT	PM1	1.55127000808716
injured_indrop_PC1_blood2_CTTAATAG.AGTTTGTA.ATCAAGTG	PM1	1.56999003887177
injured_indrop_PC1_blood2_CTTAATAG.CTGTTTCC.TTGATTCT	PM1	1.57089996337891
WASP96hr_dropseq_PI24CIRC3_TGCTCTTCCGGC.3	PM1	1.57757997512817
UNinjured_indrop_UC1_blood2_TAAGGCTC_TGGTTAAC_TCGACACC	PM1	1.57757997512817
WASP96hr_dropseq_PI24CIRC3_AACCGCTATGGA.3	PM1	1.5800199508667
injured_indrop_PC1_blood2_CTTAATAG.AGAGGTGC.ACGCAGAG	PM1	1.58926999568939
injured_tenx_B2_CTCGTACCACAGTCGC	PM1	1.59113001823425
UNinjured_tenx_B1_CGTGAGCAGGAGTTGC	PM1	1.60242998600006
injured_tenx_B2_GCAAACTAGAGCCCAA	PM1	1.60466003417969
WASP96hr_dropseq_PI24CIRC2_ACCGGGACGTGG.2	PM1	1.61296999454498
UNinjured_indrop_UC1_blood2_TAAGGCTC_ATAAGCTA_AGCGTAGG	PM1	1.61684000492096
WASP96hr_dropseq_PI24CIRC3_ATCAGTGCACAC.3	PM1	1.62235999107361
injured_tenx_B2_TACCTATAGTACACCT	PM1	1.62334001064301
UNinjured_indrop_UC1_blood2_TAAGGCTC_GACACCTG_ACCCACGA	PM1	1.62563002109528
injured_indrop_PC1_blood2_CTTAATAG.CGATCCAA.CTTTAATC	PM1	1.6275999546051
WASP96hr_dropseq_PI24CIRC3_CGGACTAACATN.3	PM1	1.62826001644135
WASP96hr_dropseq_PI24CIRC2_AGCGGGTATCAA.2	PM1	1.63453996181488
WASP96hr_dropseq_PI24CIRC3_GAGAACAAACTT.3	PM1	1.64627003669739
WASP96hr_dropseq_PI24CIRC3_GGAAGATCAGCG.3	PM1	1.64864003658295
injured_indrop_PC1_blood2_CTTAATAG.TCATACCA.TTAGTCCG	PM1	1.65340995788574
injured_tenx_B2_ATCATGGCATCCCACT	PM1	1.65786004066467
WASP96hr_dropseq_PI24CIRC3_GTCGTCAGAAAA.3	PM1	1.66478002071381
injured_tenx_B4_CTCGGAGTCCAGTATG	PM1	1.66965997219086
WASP96hr_dropseq_PI24CIRC3_GCCTAACCGGAC.3	PM1	1.67070996761322
injured_tenx_B2_GGGCATCGTAGCCTAT	PM1	1.67175996303558
WASP96hr_dropseq_PI24CIRC3_AGCCCCAATGTG.3	PM1	1.674919962883
injured_indrop_PC1_blood2_CTTAATAG.GTCAGACC.CACTCCTC	PM1	1.67527997493744
WASP96hr_dropseq_PI24CIRC3_CTGCCAGATGCA.3	PM1	1.67703998088837

Cell Barcode	Cluster Name	FBgn0046687
WASP96hr_dropseq_PI24CIRC2_CACACTGTGACC.2	PM1	1.6784600019455
WASP96hr_dropseq_PI24CIRC2_CCAGCATTAATT.2	PM1	1.67917001247406
injured_tenx_B2_CTGCCTAAGTGAAGTT	PM1	1.68523001670837
injured_indrop_PC1_blood2_CTTAATAG.ACCTGAAG.CGGGCTTT	PM1	1.68809998035431
injured_indrop_PC1_blood2_CTTAATAG.GGAGTAAG.TCCCAATC	PM1	1.68917000293732
injured_indrop_PC2_blood3_TCGCATAA.CCATCGTC.CTTGGTGT	PM1	1.68990004062653
UNinjured_indrop_UC1_blood2_TAAGGCTC_ATCAGGGA_GGGCCAAT	PM1	1.68990004062653
injured_indrop_PC2_blood3_TCGCATAA.GCCTTGTG.TAGTGGAC	PM1	1.69061994552612
injured_indrop_PC1_blood2_CTTAATAG.TCTAAACT.TTTAACAG	PM1	1.6920599937439
WASP96hr_dropseq_PI24CIRC3_TTGGTGACTGGG.3	PM1	1.7033599615097
injured_indrop_PC2_blood3_TCGCATAA.CCATCCAC.CACTCCTC	PM1	1.7033599615097
injured_tenx_B2_CCTACCAAGACTACAA	PM1	1.70594000816345
injured_tenx_B2_GAATGAAAGCAGCGTA	PM1	1.71037995815277
WASP96hr_dropseq_PI24CIRC2_ATAGCGGATGTC.2	PM1	1.71185994148254
WASP96hr_dropseq_PI24CIRC3_ACTCAAGGACAG.3	PM1	1.71521997451782
injured_tenx_B4_CACATAGAGGAGCGAG	PM1	1.71559000015259
injured_indrop_PC2_blood3_TCGCATAA.GGGATTAC.AGAAGTCC	PM1	1.71933996677399
injured_tenx_B4_GCGCGATCACCAGTTA	PM1	1.72046995162964
injured_indrop_PC2_blood3_TCGCATAA.AATCCGGC.ATTGGGCC	PM1	1.72236001491547
injured_indrop_PC2_blood3_TCGCATAA.AGAAAGTG.CTACGAGC	PM1	1.724249958992
injured_indrop_PC1_blood2_CTTAATAG.TGGAAAGC.GGTCCCTT	PM1	1.72651994228363
WASP96hr_dropseq_PI24CIRC3_AGTCTCGCAATA.3	PM1	1.72918999195099
injured_tenx_B4_ACACCGGCATGTCGAT	PM1	1.72918999195099
WASP96hr_dropseq_PI24CIRC2_GTGTGTTAGCCA.2	PM1	1.73071002960205
WASP96hr_dropseq_PI24CIRC2_AGCAGCATTACG.2	PM1	1.73763000965118
injured_indrop_PC2_blood3_TCGCATAA.GGTCTTTC.GTAATCCC	PM1	1.74032998085022
injured_tenx_B2_CCATTCGAGAAAGTGG	PM1	1.74812996387482
injured_tenx_B2_TGACAACAGAGATGAG	PM1	1.76118004322052
injured_indrop_PC2_blood3_TCGCATAA.TGAATCCT.TGTACACG	PM1	1.76558005809784
injured_tenx_B4_AAGTCTGCATACTCTT	PM1	1.76679003238678
injured_tenx_B2_ACGTCAAAGTGTGGCA	PM1	1.76961004734039
WASP96hr_dropseq_PI24CIRC2_GACCCAAACCGG.2	PM1	1.77082002162933
WASP96hr_dropseq_PI24CIRC2_CTGCACCCCCGC.2	PM1	1.7752799987793
WASP96hr_dropseq_PI24CIRC3_ACTCAACGCTGG.3	PM1	1.77752995491028
UNinjured_indrop_UC1_blood2_TAAGGCTC_AGGCAATA_AAATCGTT	PM1	1.7814199924469
WASP96hr_dropseq_PI24CIRC3_CGATTCACCCAA.3	PM1	1.78182995319366
WASP96hr_dropseq_PI24CIRC3_GTCAAATTTGAA.3	PM1	1.78429996967316
WASP96hr_dropseq_PI24CIRC3_CACGGACCATGG.3	PM1	1.78760004043579
WASP96hr_dropseq_PI24CIRC3_CTCGCAAGACCN.3	PM1	1.78760004043579
WASP96hr_dropseq_PI24CIRC3_GAAATTTCGTAA.3	PM1	1.79051005840302
WASP96hr_dropseq_PI24CIRC3_CTTCCCAGCTCT.3	PM1	1.79509997367859
injured_tenx_B4_GGGCATCTCCTCTAGC	PM1	1.80904996395111
WASP96hr_dropseq_PI24CIRC2_AACCGACCGCAA.2	PM1	1.81203997135162
injured_indrop_PC2_blood3_TCGCATAA.TCGTTCCC.CTTGGTGT	PM1	1.8189400434494
WASP96hr_dropseq_PI24CIRC2_TTGGAGGGAGCG.2	PM1	1.82327997684479
injured_tenx_B2_TCTTCGGAGATGCCAG	PM1	1.83071005344391

Cell Barcode	Cluster Name	FBgn0046687
injured_indrop_PC1_blood2_CTTAATAG.TACCCTGC.ACACTAAG	PM1	1.83779001235962
injured_indrop_PC1_blood2_CTTAATAG.ACCAGTTT.TGTGTCCT	PM1	1.83779001235962
injured_tenx_B2_GCGCAACGTATAAACG	PM1	1.84268999099731
WASP96hr_dropseq_PI24CIRC3_CGTGTCCTTTTA.3	PM1	1.84268999099731
WASP96hr_dropseq_PI24CIRC2_TTCACGGGGCTT.2	PM1	1.84403002262115
WASP96hr_dropseq_PI24CIRC3_CGGACTAACAAN.3	PM1	1.84853005409241
injured_indrop_PC1_blood2_CTTAATAG.AACTCAGT.AAATCAGA	PM1	1.84987998008728
WASP96hr_dropseq_PI24CIRC3_CCCATTCATATA.3	PM1	1.85258996486664
injured_indrop_PC2_blood3_TCGCATAA.ATACACCC.ATTGGGCC	PM1	1.85350000858307
WASP96hr_dropseq_PI24CIRC2_CCAGCATTAATA.2	PM1	1.85396003723145
injured_indrop_PC2_blood3_TCGCATAA.ATCAATCG.GACAACTT	PM1	1.85942995548248
UNinjured_indrop_UC2_blood3_TAAGGCTC_GAGGAGTG_TCTCAACC	PM1	1.86033999919891
UNinjured_indrop_UC1_blood2_TAAGGCTC_ACATGGAC_AGCGTAGG	PM1	1.86126005649567
injured_indrop_PC2_blood3_TCGCATAA.GGAGGTTT.AGCGAAGT	PM1	1.86217999458313
injured_tenx_B2_ACACCCTTCCCAGGTG	PM1	1.86355996131897
UNinjured_indrop_UC1_blood2_TAAGGCTC_ACCTTCCC_ATGGGCAC	PM1	1.86448001861572
injured_tenx_B4_CGCCAAGTCCGCTGTT	PM1	1.86724996566772
WASP96hr_dropseq_PI24CIRC3_TCGCGTACTGAT.3	PM1	1.87003004550934
UNinjured_tenx_B1_CTGCTGTTCGGTGTCG	PM1	1.87282001972198
WASP96hr_dropseq_PI24CIRC3_TATTTCTGCTAG.3	PM1	1.88172996044159
injured_indrop_PC2_blood3_TCGCATAA.CTGTTAAA.CAACAAAT	PM1	1.88196003437042
injured_indrop_PC2_blood3_TCGCATAA.AAGGGACC.AGCGAAGT	PM1	1.88220000267029
injured_indrop_PC2_blood3_TCGCATAA.TATCTGTC.TTCCGAGT	PM1	1.89266002178192
injured_indrop_PC2_blood3_TCGCATAA.CCGTCTTA.AGTGATGC	PM1	1.89362001419067
WASP96hr_dropseq_PI24CIRC3_CTCGCAAGACAN.3	PM1	1.89552998542786
injured_tenx_B4_GCTCTGTAGAACAACT	PM1	1.90228998661041
WASP96hr_dropseq_PI24CIRC3_ACGTCTTCCTTG.3	PM1	1.90374994277954
injured_indrop_PC2_blood3_TCGCATAA.CCTATTTA.CGGAATTT	PM1	1.90569996833801
UNinjured_indrop_UC1_blood2_TAAGGCTC_CGTCGAAT_GATCGTTT	PM1	1.90813994407654
WASP96hr_dropseq_PI24CIRC2_GTTCCGAATGGT.2	PM1	1.91059005260468
injured_indrop_PC2_blood3_TCGCATAA.AAGAACAG.CATTTGTT	PM1	1.91059005260468
injured_indrop_PC2_blood3_TCGCATAA.AAGAACAG.GATCGTTT	PM1	1.91108000278473
WASP96hr_dropseq_PI24CIRC3_GGTGCGGCGGAT.3	PM1	1.91304004192352
injured_indrop_PC2_blood3_TCGCATAA.GATTAAAG.GTTTACGT	PM1	1.92744994163513
injured_tenx_B4_GACGTGCCAGCTATTG	PM1	1.92946004867554
WASP96hr_dropseq_PI24CIRC2_TTCAATTATGAG.2	PM1	1.92946004867554
injured_indrop_PC1_blood2_CTTAATAG.ATGACTTG.GGACTTCC	PM1	1.9395899772644
WASP96hr_dropseq_PI24CIRC3_ACGTAGTGGCTG.3	PM1	1.95141994953156
WASP96hr_dropseq_PI24CIRC2_CAGCTCTTTAGG.2	PM1	1.96395003795624
injured_indrop_PC2_blood3_TCGCATAA.CTTTATCC.GAACGATT	PM1	1.98587000370026
WASP96hr_dropseq_PI24CIRC3_AACATCGCTCGC.3	PM1	1.9891300201416
injured_indrop_PC2_blood3_TCGCATAA.TGAGAGCG.ACGGTAGC	PM1	2.00730991363525
WASP96hr_dropseq_PI24CIRC3_TGTATAGCGAGC.3	PM1	2.02252006530762
injured_tenx_B2_ATTGGACCAAACGCGA	PM1	2.03052997589111
injured_tenx_B2_TGCGGGTCAAGTCTGT	PM1	2.03398990631104
injured_tenx_B2_GGGTTGCTCGTCTGAA	PM1	2.03629994392395

Cell Barcode	Cluster Name	FBgn0046687
WASP96hr_dropseq_PI24CIRC3_AGTTGAAGGTGG.3	PM1	2.0421199798584
WASP96hr_dropseq_PI24CIRC3_CACACCTCAGGT.3	PM1	2.04621005058289
injured_tenx_B2_ACAGCCGTCAGCACAT	PM1	2.04855990409851
WASP96hr_dropseq_PI24CIRC2_CGTCTCTAGGAA.2	PM1	2.05151009559631
injured_tenx_B2_AGATTGCGTTACGGAG	PM1	2.06222009658813
UNinjured_tenx_B1_TGTATTCGTGACTCAT	PM1	2.06522011756897
UNinjured_indrop_UC1_blood2_TAAGGCTC_GCCTGGTA_CGTACCTA	PM1	2.08965992927551
WASP96hr_dropseq_PI24CIRC3_CGGACGAAGATN.3	PM1	2.09589004516602
injured_indrop_PC2_blood3_TCGCATAA.AAACCGCC.CTTCGCAC	PM1	2.1018500328064
injured_tenx_B2_CCTTCCCGTAAGTGTA	PM1	2.10405993461609
injured_tenx_B2_CCAGCGATCAGCGATT	PM1	2.12002992630005
WASP96hr_dropseq_PI24CIRC3_GGTGCCATACTA.3	PM1	2.1219699382782
UNinjured_indrop_UC2_blood3_TAAGGCTC_TAACTTCT_GGTCTGAC	PM1	2.12390995025635
WASP96hr_dropseq_PI24CIRC3_ATAGGAAGCGGA.3	PM1	2.13173007965088
injured_tenx_B2_TCGTACCGTATAGTAG	PM1	2.1522901058197
WASP96hr_dropseq_PI24CIRC3_TGAGGACCTCGA.3	PM1	2.15430998802185
WASP96hr_dropseq_PI24CIRC3_ACGGAAAGATGC.3	PM1	2.16928005218506
injured_tenx_B2_CTTACCGAGAAGGTTT	PM1	2.20364999771118
UNinjured_indrop_UC1_blood2_TAAGGCTC_CCTTTGTC_CGAATAAT	PM1	2.20724010467529
injured_tenx_B2_CCTTCGACACGTCAGC	PM1	2.2079598903656
WASP96hr_dropseq_PI24CIRC3_CACATTCAAGAC.3	PM1	2.21812009811401
WASP96hr_dropseq_PI24CIRC3_CGCGTGGGGTCG.3	PM1	2.22323989868164
UNinjured_tenx_B1_AGCTCTCCAGGTCCAC	PM1	2.2276599407196
injured_tenx_B2_GCGCAGTAGGGTGTTG	PM1	2.22839999198914
WASP96hr_dropseq_PI24CIRC3_GCGCTTTCAGCC.3	PM1	2.22839999198914
WASP96hr_dropseq_PI24CIRC3_ACGAGAGTCGAC.3	PM1	2.23956990242004
WASP96hr_dropseq_PI24CIRC3_ACAATCTATTGT.3	PM1	2.24482989311218
WASP96hr_dropseq_PI24CIRC3_GCAATTCCAGAA.3	PM1	2.24634003639221
WASP96hr_dropseq_PI24CIRC3_ACACCCCTTAGC.3	PM1	2.27168011665344
injured_tenx_B4_GAGTCCGTCTACTTAC	PM1	2.29061007499695
injured_tenx_B2_AAATGCCCACTATCTT	PM1	2.29701995849609
injured_tenx_B2_CCAGCGACAAGCGTAG	PM1	2.30187010765076
UNinjured_indrop_UC2_blood3_TAAGGCTC_TCGACTGC_AGAACGGG	PM1	2.31491994857788
injured_tenx_B2_GGACGTCTCCGTACAA	PM1	2.31738996505737
WASP96hr_dropseq_PI24CIRC3_TAGGATAAGTCG.3	PM1	2.32650995254517
WASP96hr_dropseq_PI24CIRC3_TGCCCTGCGACG.3	PM1	2.32650995254517
WASP96hr_dropseq_PI24CIRC3_GATTCCGACTTG.3	PM1	2.34250998497009
injured_tenx_B2_TCGCGTTTCCCGGATG	PM1	2.35969996452332
injured_tenx_B2_CCGTTCACAAAGGCGT	PM1	2.36841988563538
injured_indrop_PC1_blood2_CTTAATAG.AATGTATG.GGACTTCC	PM1	2.37312006950378
injured_tenx_B2_GCAGTTAGTGTGGTTT	PM1	2.38348007202148
WASP96hr_dropseq_PI24CIRC3_GTCGGCGTAAAG.3	PM1	2.38348007202148
WASP96hr_dropseq_PI24CIRC3_GCAGTGGATTTG.3	PM1	2.39065003395081
injured_tenx_B2_ATCATCTCACACCGAC	PM1	2.39246010780334
WASP96hr_dropseq_PI24CIRC3_TTTTATACTTGT.3	PM1	2.39879989624023
injured_tenx_B2_GTGGGTCCATCACGTA	PM1	2.40154004096985

Cell Barcode	Cluster Name	FBgn0046687
UNinjured_tenx_B1_AAACGGGAGAAGGCCT	PM1	2.47996997833252
UNinjured_indrop_UC2_blood3_TAAGGCTC_ATTTGTTG_AAGCTTCT	PM1	2.52632999420166
WASP96hr_dropseq_PI24CIRC3_TTAGCTTCTACN.3	PM1	2.52845001220703
injured_indrop_PC2_blood3_TCGCATAA.CCCTCTTG.AGGAGCTT	PM1	2.55321002006531
UNinjured_tenx_B1_TCACGAAAGCGATAGC	PM1	2.5576000213623
WASP96hr_dropseq_PI24CIRC1_ATCCGTTTGAGA.1	PM1	2.60577988624573
UNinjured_tenx_B1_GATGCTATCGCCTGAG	PM1	2.61552000045776
injured_tenx_B2_GTCAAGTTCCCAAGAT	PM1	2.61610007286072
UNinjured_tenx_B1_GTGCATAGTCTGATCA	PM1	2.61787009239197
UNinjured_indrop_UC2_blood3_TAAGGCTC_GCGCTGAT_CTTCTTCG	PM1	2.64843988418579
UNinjured_tenx_B1_AAGGTTCAGCGCTCCA	PM1	2.704509973526
UNinjured_tenx_B1_CAAGATCGTTAAGACA	PM1	2.71104001998901
UNinjured_indrop_UC2_blood3_TAAGGCTC_CTAACGCC_GACAGATA	PM1	2.91847991943359
injured_tenx_B2_TGCCAAAAGTGAAGAG	PM1	2.9360499382019
WASP96hr_dropseq_PI24CIRC1_GCAAAGTACCAC.1	PM6	0.348576992750168
WASP96hr_dropseq_PI24CIRC2_ATATGCCATGTC.2	PM6	0.374864012002945
WASP96hr_dropseq_PI24CIRC2_GCGCCATATTCT.2	PM6	0.404832988977432
WASP96hr_dropseq_PI24CIRC1_GACAAGCGGAGT.1	PM6	0.407171994447708
WASP96hr_dropseq_PI24CIRC1_AAAATGGTACGA.1	PM6	0.414288997650146
WASP96hr_dropseq_PI24CIRC2_TTTCACGTAACT.2	PM6	0.449649006128311
WASP96hr_dropseq_PI24CIRC2_ACGTCACAGGCG.2	PM6	0.493128001689911
WASP96hr_dropseq_PI24CIRC2_ACGCTCTACCCG.2	PM6	0.503987014293671
WASP96hr_dropseq_PI24CIRC2_GACTTCAAGCAC.2	PM6	0.541868984699249
WASP96hr_dropseq_PI24CIRC2_GTGCATAAAAGC.2	PM6	0.55077201128006
WASP96hr_dropseq_PI24CIRC2_GCCAAGACAATG.2	PM6	0.562924981117249
WASP96hr_dropseq_PI24CIRC2_CTTCTCGAGTGC.2	PM6	0.58051997423172
WASP96hr_dropseq_PI24CIRC2_GTCATCCGCATA.2	PM6	0.587643980979919
WASP96hr_dropseq_PI24CIRC2_GAAGATTGTACT.2	PM6	0.592700004577637
WASP96hr_dropseq_PI24CIRC2_CGTGCTGTGAAG.2	PM6	0.600174009799957
WASP96hr_dropseq_PI24CIRC2_AGGTATATGGTG.2	PM6	0.616622984409332
WASP96hr_dropseq_PI24CIRC2_GGTAATTACTCG.2	PM6	0.616739988327026
WASP96hr_dropseq_PI24CIRC2_GGCCGCCCTAGA.2	PM6	0.61890697479248
WASP96hr_dropseq_PI24CIRC2_CGGTTATAACTC.2	PM6	0.641901016235352
WASP96hr_dropseq_PI24CIRC2_TCGACGGAATGG.2	PM6	0.651162028312683
WASP96hr_dropseq_PI24CIRC2_CTGGGATCCGGA.2	PM6	0.6596639752388
WASP96hr_dropseq_PI24CIRC2_CGCCCCTTTTCG.2	PM6	0.659844994544983
WASP96hr_dropseq_PI24CIRC2_TTTGCGGCATGG.2	PM6	0.662656009197235
WASP96hr_dropseq_PI24CIRC2_CCGACTGGGTTC.2	PM6	0.664025008678436
WASP96hr_dropseq_PI24CIRC2_TTGTTCCAGAGA.2	PM6	0.670072972774506
WASP96hr_dropseq_PI24CIRC2_ACTTCTTGACTC.2	PM6	0.680563986301422
WASP96hr_dropseq_PI24CIRC2_AGGAACTATGAG.2	PM6	0.683197975158691
WASP96hr_dropseq_PI24CIRC2_ATGTATACGTCC.2	PM6	0.684413015842438
WASP96hr_dropseq_PI24CIRC2_CCCGCGAGCAAC.2	PM6	0.696262001991272
WASP96hr_dropseq_PI24CIRC2_CTCTTTCCCAAT.2	PM6	0.725057005882263
WASP96hr_dropseq_PI24CIRC2_GCTGGCAACTGT.2	PM6	0.727814018726349
WASP96hr_dropseq_PI24CIRC2_TCCCAGGTCTTG.2	PM6	0.729589998722076

Cell Barcode	Cluster Name	FBgn0046687
WASP96hr_dropseq_PI24CIRC2_CATCTTAGATTG.2	PM6	0.732046008110046
WASP96hr_dropseq_PI24CIRC2_CTCGGCATGATG.2	PM6	0.733224987983704
WASP96hr_dropseq_PI24CIRC2_CGGCAAGGTCCT.2	PM6	0.735198020935059
WASP96hr_dropseq_PI24CIRC2_GTTGAACGGCGT.2	PM6	0.736415028572083
WASP96hr_dropseq_PI24CIRC2_TGACCTTTTAGA.2	PM6	0.737182021141052
WASP96hr_dropseq_PI24CIRC2_GGGCATGGCGGG.2	PM6	0.74633800983429
WASP96hr_dropseq_PI24CIRC2_CGAGGCCGAATG.2	PM6	0.747039020061493
WASP96hr_dropseq_PI24CIRC2_ATCGCCGGCCCT.2	PM6	0.753587007522583
WASP96hr_dropseq_PI24CIRC2_TAAGGATGTAAG.2	PM6	0.759286999702454
WASP96hr_dropseq_PI24CIRC2_GAATCATTCGTT.2	PM6	0.766864001750946
WASP96hr_dropseq_PI24CIRC2_TAGTCATCCTGG.2	PM6	0.768038988113403
WASP96hr_dropseq_PI24CIRC1_ACGTTTAGTGTT.1	PM6	0.770399987697601
WASP96hr_dropseq_PI24CIRC2_ACTAAGCAATAA.2	PM6	0.776942014694214
WASP96hr_dropseq_PI24CIRC2_GGCACTGTCCAC.2	PM6	0.782247006893158
WASP96hr_dropseq_PI24CIRC2_CCGCATTGACAT.2	PM6	0.79448401927948
WASP96hr_dropseq_PI24CIRC3_CGACCTCAATCA.3	PM6	0.797286987304688
WASP96hr_dropseq_PI24CIRC2_GTACTTACTACC.2	PM6	0.79849499464035
WASP96hr_dropseq_PI24CIRC2_TGCCCCATCGTC.2	PM6	0.808645009994507
WASP96hr_dropseq_PI24CIRC2_TCCAGACCTACG.2	PM6	0.810791015625
WASP96hr_dropseq_PI24CIRC2_CAAGTCATGTTA.2	PM6	0.810999989509583
WASP96hr_dropseq_PI24CIRC2_ATAATCCGTCCG.2	PM6	0.828984022140503
WASP96hr_dropseq_PI24CIRC2_ATGGTCGACAAC.2	PM6	0.829784989356995
WASP96hr_dropseq_PI24CIRC2_TTGTTCGCGTCT.2	PM6	0.833617985248566
WASP96hr_dropseq_PI24CIRC2_TCGGGCATTCAA.2	PM6	0.837105989456177
WASP96hr_dropseq_PI24CIRC2_TAGAGTCGGTGT.2	PM6	0.858814001083374
WASP96hr_dropseq_PI24CIRC2_GTTAAGGTGGAC.2	PM6	0.865293979644775
WASP96hr_dropseq_PI24CIRC2_ACAATTAATCGT.2	PM6	0.870021998882294
WASP96hr_dropseq_PI24CIRC2_CGACACATGCAA.2	PM6	0.871393978595734
WASP96hr_dropseq_PI24CIRC2_AGAGGTTCATCC.2	PM6	0.87220299243927
WASP96hr_dropseq_PI24CIRC2_CGTTGGAAGTCT.2	PM6	0.882332980632782
WASP96hr_dropseq_PI24CIRC2_ATTCGATGGTTC.2	PM6	0.887555003166199
WASP96hr_dropseq_PI24CIRC2_CCGCACCGTCAC.2	PM6	0.88945198059082
WASP96hr_dropseq_PI24CIRC2_GGTATAAACTTT.2	PM6	0.898976981639862
WASP96hr_dropseq_PI24CIRC2_TGACTACTTCTG.2	PM6	0.905332982540131
WASP96hr_dropseq_PI24CIRC2_GGCCACGCAGTG.2	PM6	0.909784972667694
WASP96hr_dropseq_PI24CIRC2_CAATGTCCGTCT.2	PM6	0.9132120013237
WASP96hr_dropseq_PI24CIRC2_ACGGTCTACCCA.2	PM6	0.913658976554871
WASP96hr_dropseq_PI24CIRC2_GTTGCTTCGTGT.2	PM6	0.914285004138947
WASP96hr_dropseq_PI24CIRC1_GATGCAGAACAG.1	PM6	0.925768971443176
WASP96hr_dropseq_PI24CIRC2_CTGCCGCCGCCC.2	PM6	0.940744996070862
WASP96hr_dropseq_PI24CIRC2_GGTAATGCTGCC.2	PM6	0.943180024623871
WASP96hr_dropseq_PI24CIRC2_AACAATTGTCTT.2	PM6	0.95183402299881
WASP96hr_dropseq_PI24CIRC2_CTTCCCTAGCTA.2	PM6	0.959070026874542
WASP96hr_dropseq_PI24CIRC2_CGGTTTCTTGCA.2	PM6	0.966476023197174
WASP96hr_dropseq_PI24CIRC2_CCCGTCCGCCGG.2	PM6	0.973801016807556
WASP96hr_dropseq_PI24CIRC2_AACGGCTAATTA.2	PM6	0.980933010578156

Cell Barcode	Cluster Name	FBgn0046687
WASP96hr_dropseq_PI24CIRC2_AGGACGCCCGCA.2	PM6	0.982813000679016
WASP96hr_dropseq_PI24CIRC2_GGCGACAATGAC.2	PM6	0.988604009151459
WASP96hr_dropseq_PI24CIRC2_AGACAATATCCG.2	PM6	0.99024897813797
injured_indrop_PC2_blood3_TCGCATAA.ATTTGTTG.TGTAGTTT	PM6	0.99115401506424
WASP96hr_dropseq_PI24CIRC2_GAGAACTTGCCG.2	PM6	1.00074005126953
WASP96hr_dropseq_PI24CIRC2_CGTTGTGACCAT.2	PM6	1.00281000137329
WASP96hr_dropseq_PI24CIRC3_GTACGGAGGTTT.3	PM6	1.00897002220154
WASP96hr_dropseq_PI24CIRC1_GGACAAAAACGT.1	PM6	1.01319003105164
WASP96hr_dropseq_PI24CIRC2_ACCATCGCTTCG.2	PM6	1.03773999214172
WASP96hr_dropseq_PI24CIRC2_CCGGCTCCACTC.2	PM6	1.0448100566864
WASP96hr_dropseq_PI24CIRC2_CGGATTTTCGAA.2	PM6	1.04797005653381
WASP96hr_dropseq_PI24CIRC2_GTCCCACAAGAT.2	PM6	1.05438005924225
WASP96hr_dropseq_PI24CIRC2_GTTCCAAGTCTG.2	PM6	1.0548700094223
WASP96hr_dropseq_PI24CIRC2_CGAATCGACTGA.2	PM6	1.05548000335693
WASP96hr_dropseq_PI24CIRC2_CCCAGACTAGCT.2	PM6	1.06076002120972
WASP96hr_dropseq_PI24CIRC2_TAACACAACACG.2	PM6	1.06634998321533
WASP96hr_dropseq_PI24CIRC2_GCGATCACGTGG.2	PM6	1.07580995559692
WASP96hr_dropseq_PI24CIRC1_ACACATCTCACG.1	PM6	1.07906997203827
injured_indrop_PC2_blood3_TCGCATAA.GGAGTAAG.ACTGCCGT	PM6	1.08963000774384
WASP96hr_dropseq_PI24CIRC2_CGTAGAAAGCTC.2	PM6	1.09595000743866
WASP96hr_dropseq_PI24CIRC2_CTATCTGATGTC.2	PM6	1.10898995399475
WASP96hr_dropseq_PI24CIRC2_CCAGCGTTTTAA.2	PM6	1.1131500005722
WASP96hr_dropseq_PI24CIRC2_GTGAACAGAGGA.2	PM6	1.11735999584198
WASP96hr_dropseq_PI24CIRC2_GTTCGAGTAACC.2	PM6	1.12382996082306
WASP96hr_dropseq_PI24CIRC2_GCGGAAGGTGCT.2	PM6	1.13496005535126
WASP96hr_dropseq_PI24CIRC2_CAAATACCTCGT.2	PM6	1.14014005661011
WASP96hr_dropseq_PI24CIRC2_GGAAGATCCCCG.2	PM6	1.15037000179291
WASP96hr_dropseq_PI24CIRC2_TTTAAACAGAAT.2	PM6	1.15541005134583
WASP96hr_dropseq_PI24CIRC2_GTACGGGGAGTT.2	PM6	1.16383004188538
WASP96hr_dropseq_PI24CIRC2_ACATTCAACCTT.2	PM6	1.16846001148224
WASP96hr_dropseq_PI24CIRC1_CACACCCAGCCA.1	PM6	1.16848003864288
WASP96hr_dropseq_PI24CIRC2_GAGGGTTACTGC.2	PM6	1.17569994926453
WASP96hr_dropseq_PI24CIRC2_CGGGTGATGTTA.2	PM6	1.18325996398926
WASP96hr_dropseq_PI24CIRC2_GAATGCCGGGGC.2	PM6	1.18467998504639
WASP96hr_dropseq_PI24CIRC2_TTCCCGCCACAT.2	PM6	1.18588995933533
WASP96hr_dropseq_PI24CIRC2_TAGCGGAATTGC.2	PM6	1.19413995742798
WASP96hr_dropseq_PI24CIRC2_ATTTTTGCAGAA.2	PM6	1.19937002658844
WASP96hr_dropseq_PI24CIRC2_GGCTTTGTTAGT.2	PM6	1.20360004901886
WASP96hr_dropseq_PI24CIRC2_GTCAATCCAAGA.2	PM6	1.20457994937897
WASP96hr_dropseq_PI24CIRC2_GTGGGGGGGCAA.2	PM6	1.20655000209808
WASP96hr_dropseq_PI24CIRC2_TCGCCTGCCTTA.2	PM6	1.20873999595642
WASP96hr_dropseq_PI24CIRC2_CGCGATCGTCTG.2	PM6	1.21581995487213
WASP96hr_dropseq_PI24CIRC2_TTAATCTTGCGC.2	PM6	1.22763001918793
WASP96hr_dropseq_PI24CIRC2_GCAACAGCATGC.2	PM6	1.23943996429443
WASP96hr_dropseq_PI24CIRC2_TAGTAGTCAACG.2	PM6	1.24778997898102
WASP96hr_dropseq_PI24CIRC2_TTATTCGGCACG.2	PM6	1.26243996620178

Cell Barcode	Cluster Name	FBgn0046687
WASP96hr_dropseq_PI24CIRC2_TTAGACTGGGGC.2	PM6	1.26531004905701
WASP96hr_dropseq_PI24CIRC2_CTTGTCCCGATA.2	PM6	1.26552999019623
WASP96hr_dropseq_PI24CIRC1_ACGTTGACTAGA.1	PM6	1.29120004177094
WASP96hr_dropseq_PI24CIRC2_TTGTGGACATCA.2	PM6	1.29966998100281
WASP96hr_dropseq_PI24CIRC2_CCGAGAGCTAGG.2	PM6	1.3105800151825
WASP96hr_dropseq_PI24CIRC1_GACCAGCGGAGT.1	PM6	1.3126300573349
WASP96hr_dropseq_PI24CIRC2_TGCTGGGACCTA.2	PM6	1.31500995159149
WASP96hr_dropseq_PI24CIRC2_GAGAATGTCTTT.2	PM6	1.31660997867584
WASP96hr_dropseq_PI24CIRC2_CGCCGAATTCCC.2	PM6	1.32507002353668
WASP96hr_dropseq_PI24CIRC2_GTACATTTTATA.2	PM6	1.32783997058868
WASP96hr_dropseq_PI24CIRC2_CCACACCACCTG.2	PM6	1.33153998851776
injured_indrop_PC1_blood2_CTTAATAG.CGACATTT.CGTACCTA	PM6	1.33302998542786
injured_indrop_PC1_blood2_CTTAATAG.CGTGGGAT.TTCGTTCC	PM6	1.33653998374939
WASP96hr_dropseq_PI24CIRC2_CGTCCATGATCA.2	PM6	1.34203994274139
WASP96hr_dropseq_PI24CIRC1_AAGATCCAGCTA.1	PM6	1.34538996219635
WASP96hr_dropseq_PI24CIRC2_GCTACCTCCCGG.2	PM6	1.35183000564575
injured_indrop_PC1_blood2_CTTAATAG.TAGTCTCT.GAAGCACT	PM6	1.37677001953125
WASP96hr_dropseq_PI24CIRC2_GGCTTATAGTAT.2	PM6	1.38400995731354
WASP96hr_dropseq_PI24CIRC2_ACCGCAAGCGCA.2	PM6	1.39151000976562
WASP96hr_dropseq_PI24CIRC2_CCGAAGAACACC.2	PM6	1.39187002182007
injured_indrop_PC1_blood2_CTTAATAG.TACCTCCC.AATGAATG	PM6	1.39361000061035
WASP96hr_dropseq_PI24CIRC2_GTACGAGCTTCG.2	PM6	1.4123500585556
WASP96hr_dropseq_PI24CIRC2_CTCAGGTTCGTG.2	PM6	1.41422998905182
WASP96hr_dropseq_PI24CIRC2_AGGATTAATCCA.2	PM6	1.4237300157547
WASP96hr_dropseq_PI24CIRC2_CCGATCTAGTAG.2	PM6	1.44491004943848
WASP96hr_dropseq_PI24CIRC2_GCCTTACTGAGC.2	PM6	1.44491004943848
WASP96hr_dropseq_PI24CIRC2_TTTGATTTTACT.2	PM6	1.4554899930954
WASP96hr_dropseq_PI24CIRC2_CCATACAAGTTG.2	PM6	1.46059000492096
WASP96hr_dropseq_PI24CIRC2_CATTATTGTTAA.2	PM6	1.47045004367828
WASP96hr_dropseq_PI24CIRC2_CCTTACGAGGAT.2	PM6	1.47680997848511
WASP96hr_dropseq_PI24CIRC2_GGGTCCAGGGGN.2	PM6	1.49224996566772
WASP96hr_dropseq_PI24CIRC2_GACAACAGCCCA.2	PM6	1.58177995681763
WASP96hr_dropseq_PI24CIRC2_CGCCGTATTGAT.2	PM6	1.59739005565643
WASP96hr_dropseq_PI24CIRC2_AGAATGGTGGGA.2	PM6	1.61168003082275
WASP96hr_dropseq_PI24CIRC2_GCCGATACCAAC.2	PM6	1.64038002490997
WASP96hr_dropseq_PI24CIRC2_ATCAGTAACCAC.2	PM6	1.68414998054504
WASP96hr_dropseq_PI24CIRC2_TATAAGGGTGAT.2	PM6	1.69593000411987
WASP96hr_dropseq_PI24CIRC2_GATAGAGCCAAA.2	PM6	1.76337003707886
WASP96hr_dropseq_PI24CIRC2_GCTACGCGGGGA.2	PM6	1.764240026474
WASP96hr_dropseq_PI24CIRC2_CAGCTCTCCATT.2	PM6	1.78286004066467
WASP96hr_dropseq_PI24CIRC2_TGCGCAGGGGAC.2	PM6	1.84328997135162
WASP96hr_dropseq_PI24CIRC2_GCGTTAAAATCG.2	PM6	1.88433003425598
WASP96hr_dropseq_PI24CIRC2_CATTATTGTTAG.2	PM6	1.90878999233246
WASP96hr_dropseq_PI24CIRC2_GATTTATGCAGA.2	PM6	1.93664002418518
WASP96hr_dropseq_PI24CIRC2_GCTTGCAGGAGT.2	PM6	1.95831000804901
WASP96hr_dropseq_PI24CIRC2_TAGCGTGGGTAA.2	PM6	2.18523001670837

Cell Barcode	Cluster Name	FBgn0046687
WASP96hr_dropseq_PI24CIRC1_GCGTCCTTATCT.1	PM7	0.304210990667343
WASP96hr_dropseq_PI24CIRC2_TTGTCTTCAGCC.2	PM7	0.353114992380142
WASP96hr_dropseq_PI24CIRC2_TTACGAATGACT.2	PM7	0.381300985813141
WASP96hr_dropseq_PI24CIRC2_AATCGTGATTAA.2	PM7	0.426010996103287
WASP96hr_dropseq_PI24CIRC2_ATGATGCGCAGG.2	PM7	0.437220007181168
WASP96hr_dropseq_PI24CIRC2_GAGTAAGCCAGG.2	PM7	0.43970000743866
WASP96hr_dropseq_PI24CIRC2_TTAACATCCTTC.2	PM7	0.448419004678726
WASP96hr_dropseq_PI24CIRC2_ACGGGAACCCCG.2	PM7	0.448603004217148
WASP96hr_dropseq_PI24CIRC1_ACCAGAGATATA.1	PM7	0.45202699303627
WASP96hr_dropseq_PI24CIRC2_ACTCAGACCACT.2	PM7	0.460628002882004
WASP96hr_dropseq_PI24CIRC2_CAATTGACCAAT.2	PM7	0.463037997484207
WASP96hr_dropseq_PI24CIRC2_AGACTTCCATTC.2	PM7	0.465759992599487
WASP96hr_dropseq_PI24CIRC2_GACCATTTTCAC.2	PM7	0.471870988607407
WASP96hr_dropseq_PI24CIRC2_TCCAATATGGTC.2	PM7	0.486012995243073
WASP96hr_dropseq_PI24CIRC2_GCTGCCCATGCA.2	PM7	0.49065899848938
WASP96hr_dropseq_PI24CIRC2_CAGAAGTATTCT.2	PM7	0.526105999946594
WASP96hr_dropseq_PI24CIRC2_GTATCAAGATCC.2	PM7	0.543528974056244
WASP96hr_dropseq_PI24CIRC2_GCTATTAAAATT.2	PM7	0.543528974056244
WASP96hr_dropseq_PI24CIRC2_AGCGGTGATAAT.2	PM7	0.54559600353241
WASP96hr_dropseq_PI24CIRC2_GGTCATAAATTG.2	PM7	0.565935015678406
WASP96hr_dropseq_PI24CIRC2_ATTGCGTTTCCG.2	PM7	0.576973021030426
WASP96hr_dropseq_PI24CIRC1_TCGAATGTTACA.1	PM7	0.594186007976532
WASP96hr_dropseq_PI24CIRC2_CTTATCGATTCT.2	PM7	0.599062979221344
WASP96hr_dropseq_PI24CIRC2_CCTGCCCGCCAT.2	PM7	0.608493983745575
WASP96hr_dropseq_PI24CIRC2_CCCTCATTTCTC.2	PM7	0.627246975898743
WASP96hr_dropseq_PI24CIRC2_TGACATGGTTAA.2	PM7	0.629203975200653
UNinjured_tenx_B3_AGATCTGGTTAGGGTG	PM7	0.657595992088318
WASP96hr_dropseq_PI24CIRC1_TGTAGCATAAAC.1	PM7	0.660071015357971
WASP96hr_dropseq_PI24CIRC2_CCGGTAAAGTTC.2	PM7	0.664254009723663
injured_tenx_B4_CTGGTCTGTTTAAGCC	PM7	0.668214023113251
WASP96hr_dropseq_PI24CIRC2_TCGGAGTTTGAC.2	PM7	0.69209897518158
WASP96hr_dropseq_PI24CIRC2_GCGAACGTTTGC.2	PM7	0.693346977233887
injured_tenx_B4_GTCTCGTCAGCGAACA	PM7	0.705107986927032
WASP96hr_dropseq_PI24CIRC2_ATCTGTGGGCTA.2	PM7	0.717882990837097
WASP96hr_dropseq_PI24CIRC2_AAGCCGTCCACG.2	PM7	0.723713994026184
WASP96hr_dropseq_PI24CIRC2_CCGTTGGAGGTA.2	PM7	0.723742008209229
WASP96hr_dropseq_PI24CIRC1_GGGGCGGTCATA.1	PM7	0.730145990848541
WASP96hr_dropseq_PI24CIRC2_TGTAGGCGCCAA.2	PM7	0.732662975788116
WASP96hr_dropseq_PI24CIRC2_TCCGAGTCTGAC.2	PM7	0.746630012989044
WASP96hr_dropseq_PI24CIRC2_GCCATCAGTTAG.2	PM7	0.779106020927429
WASP96hr_dropseq_PI24CIRC1_AGGGATGAAATG.1	PM7	0.785004019737244
WASP96hr_dropseq_PI24CIRC1_TTTCGATTCCTG.1	PM7	0.790085971355438
WASP96hr_dropseq_PI24CIRC2_GTTCTTGATTCG.2	PM7	0.80630499124527
UNinjured_tenx_B3_ACTGTCCAGATGTTAG	PM7	0.811416983604431
WASP96hr_dropseq_PI24CIRC3_ACTGAGCACGGG.3	PM7	0.824931025505066
WASP96hr_dropseq_PI24CIRC2_CGTGTAGACGCG.2	PM7	0.861724972724915

Cell Barcode	Cluster Name	FBgn0046687
WASP96hr_dropseq_PI24CIRC2_TCTTAACCTTTG.2	PM7	0.862634003162384
WASP96hr_dropseq_PI24CIRC2_ACATAGAACATG.2	PM7	0.877202987670898
WASP96hr_dropseq_PI24CIRC2_ACGATCGGCACT.2	PM7	0.897597014904022
WASP96hr_dropseq_PI24CIRC2_TATGAGAGGTTA.2	PM7	0.897984981536865
WASP96hr_dropseq_PI24CIRC2_TGATATCTCTCT.2	PM7	0.904543995857239
WASP96hr_dropseq_PI24CIRC2_GATAAACAATTA.2	PM7	0.915811002254486
WASP96hr_dropseq_PI24CIRC2_AGTGATAAAGAT.2	PM7	0.929975986480713
WASP96hr_dropseq_PI24CIRC2_CTAGGTTTATAC.2	PM7	0.946704983711243
WASP96hr_dropseq_PI24CIRC2_GAGCTGTTGATA.2	PM7	0.953742980957031
WASP96hr_dropseq_PI24CIRC2_ATAGAGTTAGTT.2	PM7	0.96001398563385
WASP96hr_dropseq_PI24CIRC3_CTTACGAATCCT.3	PM7	0.972060024738312
WASP96hr_dropseq_PI24CIRC2_CCTCCGGTGTCT.2	PM7	0.98286497592926
injured_tenx_B4_GCTGCAGTCGGCCGAT	PM7	0.996619999408722
WASP96hr_dropseq_PI24CIRC1_TTATTAGAGGCT.1	PM7	0.998239994049072
WASP96hr_dropseq_PI24CIRC2_GACCAAACATCT.2	PM7	1.01080000400543
WASP96hr_dropseq_PI24CIRC2_TTAAAGTGCAGG.2	PM7	1.01655995845795
WASP96hr_dropseq_PI24CIRC2_CTACGGCTGGCC.2	PM7	1.01944005489349
WASP96hr_dropseq_PI24CIRC2_GTATTACTGTTG.2	PM7	1.0262199640274
WASP96hr_dropseq_PI24CIRC3_GCAACACGCGGC.3	PM7	1.0448499917984
WASP96hr_dropseq_PI24CIRC2_CTAATGGATGCT.2	PM7	1.04978001117706
injured_tenx_B4_TGAGCATGTCGAATCT	PM7	1.0777200460434
WASP96hr_dropseq_PI24CIRC2_AACTACGACGAG.2	PM7	1.07824003696442
WASP96hr_dropseq_PI24CIRC2_TGCTGGCGCAAG.2	PM7	1.10565996170044
WASP96hr_dropseq_PI24CIRC2_TACCAGGGCAGT.2	PM7	1.10722005367279
WASP96hr_dropseq_PI24CIRC2_GGCTACGGGACA.2	PM7	1.13654005527496
WASP96hr_dropseq_PI24CIRC2_GGAAATGCGAGA.2	PM7	1.146399974823
WASP96hr_dropseq_PI24CIRC2_GATTCGTATCGC.2	PM7	1.14757001399994
WASP96hr_dropseq_PI24CIRC2_AAGCAAAGCTGT.2	PM7	1.15630996227264
WASP96hr_dropseq_PI24CIRC2_TACTTCCGGCAT.2	PM7	1.15845000743866
WASP96hr_dropseq_PI24CIRC2_TCTCAATTTGGA.2	PM7	1.19985997676849
WASP96hr_dropseq_PI24CIRC2_CCCGGGATAACG.2	PM7	1.20246005058289
WASP96hr_dropseq_PI24CIRC2_GGTGTCTGTCAG.2	PM7	1.2051500082016
WASP96hr_dropseq_PI24CIRC2_AGTCGGTAGAGT.2	PM7	1.20605003833771
WASP96hr_dropseq_PI24CIRC2_TTGGCTGTTAAG.2	PM7	1.21903002262115
WASP96hr_dropseq_PI24CIRC2_CCTCCGACTTTT.2	PM7	1.22017002105713
injured_tenx_B4_CTTACCGAGTCAAGGC	PM7	1.24197995662689
injured_indrop_PC1_blood2_CTTAATAG.GAGAAACC.AAACAGGG	PM7	1.24937999248505
WASP96hr_dropseq_PI24CIRC2_CCGCCTCGTGCG.2	PM7	1.2539199590683
injured_tenx_B4_AGCCTAATCTTCTGGC	PM7	1.25490999221802
WASP96hr_dropseq_PI24CIRC2_ACGACAGGTGTA.2	PM7	1.25626003742218
injured_indrop_PC2_blood3_TCGCATAA.AGACCTCA.ACTAGCCA	PM7	1.27833998203278
WASP96hr_dropseq_PI24CIRC2_CTCCCGGCGCAG.2	PM7	1.30631005764008
WASP96hr_dropseq_PI24CIRC2_GGTCGTATCATG.2	PM7	1.31123995780945
WASP96hr_dropseq_PI24CIRC2_GGTAGTGGTGGA.2	PM7	1.33749997615814
WASP96hr_dropseq_PI24CIRC2_CCTCCCAAGTAT.2	PM7	1.40012001991272
injured_indrop_PC2_blood3_TCGCATAA.CGTCGAAT.CACGGACT	PM7	1.40697002410889

Cell Barcode	Cluster Name	FBgn0046687
WASP96hr_dropseq_PI24CIRC2_GCCTACATGTCA.2	PM7	1.40929996967316
WASP96hr_dropseq_PI24CIRC2_ACATAGACGAAC.2	PM7	1.41054999828339
WASP96hr_dropseq_PI24CIRC2_GAACTCGTTGGC.2	PM7	1.42334997653961
injured_tenx_B2_TTCTCCTTCAGTCCCT	PM7	1.44342994689941
WASP96hr_dropseq_PI24CIRC2_AAAGGTCATCCC.2	PM7	1.45590996742249
WASP96hr_dropseq_PI24CIRC2_GTTCCGCGCCAA.2	PM7	1.47102999687195
WASP96hr_dropseq_PI24CIRC2_TAACAGGTAACC.2	PM7	1.47185003757477
UNinjured_tenx_B3_TCACAAGTCTGAAAGA	PM7	1.48269999027252
WASP96hr_dropseq_PI24CIRC2_AACTACGACGAC.2	PM7	1.49412000179291
injured_indrop_PC2_blood3_TCGCATAA.AGCTTCGA.GACAACTT	PM7	1.49546003341675
injured_tenx_B2_CTTCTCTCACTTAAGC	PM7	1.50683999061584
WASP96hr_dropseq_PI24CIRC2_GGTCGTATCATC.2	PM7	1.53965997695923
WASP96hr_dropseq_PI24CIRC2_GAGATCTCGTCG.2	PM7	1.54729998111725
WASP96hr_dropseq_PI24CIRC2_TTTCTCCCCACN.2	PM7	1.56639003753662
injured_tenx_B2_GACCTGGCACTTCTGC	PM7	1.5706000328064
WASP96hr_dropseq_PI24CIRC1_TCGCATGTTACA.1	PM7	1.57271003723145
injured_indrop_PC1_blood2_CTTAATAG.CTCGGTGA.GCGCATTC	PM7	1.59362995624542
injured_indrop_PC2_blood3_TCGCATAA.CTCTATAG.GAACGATT	PM7	1.62399995326996
WASP96hr_dropseq_PI24CIRC2_GACGATTAGCAG.2	PM7	1.62743997573853
injured_indrop_PC2_blood3_TCGCATAA.CGAAACTC.GCAGCATG	PM7	1.64593005180359
injured_indrop_PC2_blood3_TCGCATAA.CGGTGAGT.AAGTAATC	PM7	1.68379998207092
WASP96hr_dropseq_PI24CIRC2_GTGAGTTGGACC.2	PM7	1.7043399810791
WASP96hr_dropseq_PI24CIRC2_GCCCTATTGCAC.2	PM7	1.70957005023956
injured_tenx_B2_GCAGCCACACACATGT	PM7	1.73531997203827
WASP96hr_dropseq_PI24CIRC2_AGAAACGCAATT.2	PM7	1.78224003314972
injured_indrop_PC2_blood3_TCGCATAA.CTTAGGCC.AGCGTAGG	PM7	1.78802001476288
WASP96hr_dropseq_PI24CIRC2_GGTCGTATCATA.2	PM7	1.79342997074127
injured_indrop_PC2_blood3_TCGCATAA.CTCAAACA.GAGGCTGA	PM7	1.81161999702454
WASP96hr_dropseq_PI24CIRC2_CCTTTCAATTCA.2	PM7	1.88362002372742
injured_indrop_PC1_blood2_CTTAATAG.TCCGCTGT.GTCCAAAG	PM7	1.92021000385284
WASP96hr_dropseq_PI24CIRC2_CGCGTCCCTACA.2	PM7	1.92554998397827
injured_indrop_PC1_blood2_CTTAATAG.TGGTTAAC.CGGAATTT	PM7	1.95297002792358
injured_indrop_PC2_blood3_TCGCATAA.TAAACCGA.CAGACCAT	PM7	2.01911997795105
injured_indrop_PC2_blood3_TCGCATAA.ATCAGGGA.GTCCATGT	PM7	2.01968002319336
WASP96hr_dropseq_PI24CIRC2_CACATCCATGCG.2	PM7	2.09089994430542
WASP96hr_dropseq_PI24CIRC2_CTCATAAGCTCG.2	PM7	2.24542999267578
injured_tenx_B2_TGTCCCAGTGCAGACA	PM7	2.33658003807068
WASP96hr_dropseq_PI24CIRC2_GGGGCACTCTAG.2	PM7	2.63301992416382
WASP96hr_dropseq_PI24CIRC3_GTCTGTGGTTCG.3	LM2	0.263074994087219
WASP96hr_dropseq_PI24CIRC3_TGTTTGACGTCC.3	LM2	0.360619008541107
WASP96hr_dropseq_PI24CIRC3_ACCTCCTAAATC.3	LM2	0.375990986824036
WASP96hr_dropseq_PI24CIRC3_CGGCTCTCGTCT.3	LM2	0.446505010128021
WASP96hr_dropseq_PI24CIRC2_ATGCAAAAGGTT.2	LM2	0.4774030148983
WASP96hr_dropseq_PI24CIRC2_GCTCAGAGTCAG.2	LM2	0.524523973464966
WASP96hr_dropseq_PI24CIRC3_AGCCATGCGGAG.3	LM2	0.527667999267578
WASP96hr_dropseq_PI24CIRC3_TTTTGGACTTGA.3	LM2	0.564490973949432

Cell Barcode	Cluster Name	FBgn0046687
WASP96hr_dropseq_PI24CIRC3_CGTTTAACGACA.3	LM2	0.58194500207901
WASP96hr_dropseq_PI24CIRC3_GGGCCCCTTTCC.3	LM2	0.584042012691498
WASP96hr_dropseq_PI24CIRC3_GATGCTGTTTCG.3	LM2	0.588141977787018
WASP96hr_dropseq_PI24CIRC1_AACATCCTATTC.1	LM2	0.612529993057251
WASP96hr_dropseq_PI24CIRC2_TCTAGCATTATG.2	LM2	0.627063989639282
WASP96hr_dropseq_PI24CIRC2_TAGCACTTCACT.2	LM2	0.630208015441895
injured_tenx_B4_TCAGCTCCAGCCTATA	LM2	0.647929012775421
WASP96hr_dropseq_PI24CIRC3_GTATAATGGCGA.3	LM2	0.650547981262207
WASP96hr_dropseq_PI24CIRC3_GGTCCTTGGTGT.3	LM2	0.655584990978241
WASP96hr_dropseq_PI24CIRC3_ACAAATAGTTGC.3	LM2	0.692746996879578
WASP96hr_dropseq_PI24CIRC1_TACTCCTTAGCC.1	LM2	0.706284999847412
WASP96hr_dropseq_PI24CIRC3_CCTTTCCTGGAG.3	LM2	0.712229013442993
WASP96hr_dropseq_PI24CIRC3_GGCCCCGTCGCT.3	LM2	0.716916978359222
WASP96hr_dropseq_PI24CIRC3_CTCTAATCAAGT.3	LM2	0.731429994106293
WASP96hr_dropseq_PI24CIRC3_TAGTTCCAATCC.3	LM2	0.731765985488892
WASP96hr_dropseq_PI24CIRC3_AGCCGTCCAAGT.3	LM2	0.736159980297089
WASP96hr_dropseq_PI24CIRC3_GCTGAGTGCGGT.3	LM2	0.741240978240967
injured_tenx_B4_TCGTAGAGTCCGAACC	LM2	0.751094996929169
WASP96hr_dropseq_PI24CIRC3_GTTCTATAACCA.3	LM2	0.766184985637665
WASP96hr_dropseq_PI24CIRC3_GCCCCAAAGGAT.3	LM2	0.77980899810791
injured_tenx_B4_ATTACTCGTACTCGCG	LM2	0.78064101934433
WASP96hr_dropseq_PI24CIRC3_TGGTTAAGTTTT.3	LM2	0.802007019519806
WASP96hr_dropseq_PI24CIRC3_TCTATTCCGTTC.3	LM2	0.821346998214722
WASP96hr_dropseq_PI24CIRC3_CCGTCAGGGCTT.3	LM2	0.87034398317337
WASP96hr_dropseq_PI24CIRC3_GGCGTGAATGGG.3	LM2	0.88696700334549
WASP96hr_dropseq_PI24CIRC3_AGTAACTTCTAG.3	LM2	0.899236977100372
WASP96hr_dropseq_PI24CIRC3_TTTATTAGACTA.3	LM2	0.901750981807709
WASP96hr_dropseq_PI24CIRC3_TACCTACGCGCC.3	LM2	0.91473400592804
injured_tenx_B4_TTGGAACGTGCATCTA	LM2	0.918335974216461
injured_tenx_B2_GACCAATAGCCAGTAG	LM2	0.94481098651886
injured_tenx_B2_CGATGTATCAAACGGG	LM2	0.98617297410965
injured_tenx_B2_GGGCATCAGCAGCGTA	LM2	0.989983022212982
injured_tenx_B2_AACTCAGCAATCTGCA	LM2	1.00007998943329
injured_tenx_B4_CTCCTAGGTAAATGAC	LM2	1.00055003166199
WASP96hr_dropseq_PI24CIRC3_GGAGACTAACTT.3	LM2	1.03073000907898
WASP96hr_dropseq_PI24CIRC3_TACAAACCGCGC.3	LM2	1.03329002857208
WASP96hr_dropseq_PI24CIRC3_GACCGCTCCTAC.3	LM2	1.04707002639771
injured_tenx_B2_CGACTTCAGCGCCTTG	LM2	1.0587899684906
WASP96hr_dropseq_PI24CIRC3_GTGTCACAGGGG.3	LM2	1.10959994792938
WASP96hr_dropseq_PI24CIRC3_TCACAACTGTGG.3	LM2	1.12055003643036
WASP96hr_dropseq_PI24CIRC3_CGCTTGCATGCT.3	LM2	1.14029002189636
WASP96hr_dropseq_PI24CIRC1_GAGGATGTATGG.1	LM2	1.15067005157471
injured_indrop_PC2_blood3_TCGCATAA.GGTAAATC.AAACGCCT	LM2	1.19037997722626
WASP96hr_dropseq_PI24CIRC1_CGCCCCCAAATC.1	LM2	1.199049949646
WASP96hr_dropseq_PI24CIRC3_GCTACGGACTTA.3	LM2	1.19985997676849
WASP96hr_dropseq_PI24CIRC3_CACCCTTTTTAC.3	LM2	1.2014000415802

Cell Barcode	Cluster Name	FBgn0046687
WASP96hr_dropseq_PI24CIRC3_AGTGTACATTTG.3	LM2	1.21332001686096
injured_tenx_B2_ACTTGTTGTGTCTGAT	LM2	1.21984004974365
WASP96hr_dropseq_PI24CIRC3_GGCGTGAATGGA.3	LM2	1.22864997386932
injured_indrop_PC2_blood3_TCGCATAA.GTCAAGAG.ACACTAAG	LM2	1.29831004142761
WASP96hr_dropseq_PI24CIRC3_ACCTGGGCAAAA.3	LM2	1.32163000106812
injured_indrop_PC1_blood2_CTTAATAG.GCAAACTG.ATATACCT	LM2	1.34423995018005
WASP96hr_dropseq_PI24CIRC3_TATGACTGCACC.3	LM2	1.35502994060516
injured_tenx_B4_GTTAAGCGTTAAAGAC	LM2	1.37633001804352
injured_tenx_B4_CAACCTCAGACACTAA	LM2	1.37766003608704
injured_indrop_PC1_blood2_CTTAATAG.TAGCCACA.TGCGTATC	LM2	1.3938399553299
injured_tenx_B2_ATTGGTGTCGGATGGA	LM2	1.39543998241425
WASP96hr_dropseq_PI24CIRC3_ACCCTTTTTGAT.3	LM2	1.46126997470856
injured_indrop_PC1_blood2_CTTAATAG.AAGTCCAA.AGCGCCTT	LM2	1.46967995166779
injured_indrop_PC1_blood2_CTTAATAG.AGCTCCAC.AAAGCCTA	LM2	1.50058996677399
injured_indrop_PC1_blood2_CTTAATAG.AAACGTGA.ATGCATGG	LM2	1.51426994800568
injured_indrop_PC2_blood3_TCGCATAA.CCCGTAAG.CGAATAAT	LM2	1.54197001457214
injured_tenx_B2_ACTGCTCCATCACGTA	LM2	1.5436999797821
WASP96hr_dropseq_PI24CIRC3_GTTCGGCCCGTG.3	LM2	1.55597996711731
injured_indrop_PC1_blood2_CTTAATAG.ACAAGTAG.ACTAGCCA	LM2	1.5639899969101
WASP96hr_dropseq_PI24CIRC3_GTGCCCGTAGGA.3	LM2	1.590980052948
injured_tenx_B2_CGCGTTTGTCGCTTCT	LM2	1.68352997303009
WASP96hr_dropseq_PI24CIRC1_GAGGATTATGGG.1	LM2	1.70299005508423
injured_tenx_B2_ACGATGTGTGTGACCC	LM2	1.70428001880646
WASP96hr_dropseq_PI24CIRC3_GAGGGTACTCGT.3	LM2	1.75402998924255
injured_tenx_B2_GTGTGCGGTCGACTGC	LM2	1.82512998580933
injured_tenx_B2_TATCAGGGTACAGTTC	LM2	1.90327000617981
injured_indrop_PC1_blood2_CTTAATAG.ACCACAGA.GGTCACAG	LM2	1.93603003025055
injured_tenx_B2_GCACATATCACTATTC	LM2	1.93907999992371
injured_indrop_PC1_blood2_CTTAATAG.GCCTTGTG.ATGCATGG	LM2	1.98641002178192
injured_tenx_B2_CACACAACATTAGCCA	LM2	1.99751996994019
injured_indrop_PC2_blood3_TCGCATAA.GTGAACTC.TACAAACT	LM2	2.00342011451721
WASP96hr_dropseq_PI24CIRC3_TAAAATCCTATT.3	LM2	2.01122999191284
injured_tenx_B2_GGCGTGTAGTTAACGA	LM2	2.04519009590149
injured_indrop_PC1_blood2_CTTAATAG.CCGATACG.ATGTTGGC	LM2	2.09028005599976
injured_indrop_PC1_blood2_CTTAATAG.AGAAAGTG.GCCTCTTT	LM2	2.0940101146698
injured_tenx_B2_TTGGCAAGTTACCAGT	LM2	2.10616993904114
injured_tenx_B2_ATCGAGTTCATTTGGG	LM2	2.24275994300842
WASP96hr_dropseq_PI24CIRC3_CGCACCCATACC.3	LM2	2.29140996932983
injured_tenx_B2_GTTAAGCTCAACCATG	LM2	2.32052993774414
injured_tenx_B2_CTAACTTCAATCGGTT	LM2	2.54559993743896
injured_indrop_PC1_blood2_CTTAATAG.AAGAGTAT.ATACTCTT	PM10	1.19341003894806
UNinjured_tenx_B3_CTAGAGTCACAGCCCA	PM10	1.20950996875763
UNinjured_indrop_UC1_blood2_TAAGGCTC_ATTGAAGT_TCATATGG	PM10	1.31940996646881
UNinjured_indrop_UC1_blood2_TAAGGCTC_AGCGTGGT_ATCTTTGT	PM10	1.39772999286652
UNinjured_indrop_UC1_blood2_TAAGGCTC_ACCAACCC_GTACACTC	PM10	1.48746001720428
injured_tenx_B4_AGGGAGTAGTGGGTTG	PM10	1.50411999225616

Cell Barcode	Cluster Name	FBgn0046687
UNinjured_indrop_UC1_blood2_TAAGGCTC_CGAGGCTA_GGCGGTTT	PM10	1.5436999797821
UNinjured_tenx_B1_CATGGCGCACTGAAGG	PM10	1.55215001106262
injured_indrop_PC2_blood3_TCGCATAA.GCGGTAAC.CTTCTTCG	PM10	1.56579005718231
UNinjured_indrop_UC1_blood2_TAAGGCTC_AATACATC_GTTGCACG	PM10	1.57666003704071
injured_tenx_B2_AGCATACCAGATGGCA	PM10	1.69351005554199
UNinjured_tenx_B1_ATCGAGTCAAGCCATT	PM10	1.85124003887177
injured_tenx_B2_GACAGAGGTCAAGCGA	PM10	1.93096995353699
injured_tenx_B4_CGATCGGTCACGACTA	CC1	0.752161026000977
WASP96hr_dropseq_PI24CIRC3_CCATTTCTCGAG.3	CC1	1.08701002597809
WASP96hr_dropseq_PI24CIRC3_GGAGATTGCACC.3	CC1	1.24974000453949
injured_tenx_B2_GGATTACCATCACAAC	CC1	1.26249003410339
injured_tenx_B4_CGTCAGGTCATAAAGG	CC1	1.37434005737305
UNinjured_indrop_UC1_blood2_TAAGGCTC_ATCCCACC_CTTCGATT	PM11	1.32710003852844
UNinjured_indrop_UC1_blood2_TAAGGCTC_CAATTCTC_CTTTCTTT	PM11	1.42660999298096
UNinjured_indrop_UC1_blood2_TAAGGCTC_AGAGGTGC_GACAAAGG	PM11	1.44516003131866
UNinjured_indrop_UC1_blood2_TAAGGCTC_CCTTTACA_AACAATCC	PM11	1.4766800403595
UNinjured_indrop_UC1_blood2_TAAGGCTC_ACATGGAC_CAAGTCAT	PM11	1.53679001331329
UNinjured_tenx_B1_TGCTGCTGTACCCAAT	PM11	1.56608998775482
UNinjured_indrop_UC1_blood2_TAAGGCTC_CCCGTTCT_GGGAAGGT	PM11	1.6342099905014
injured_indrop_PC1_blood2_CTTAATAG.ATCCGCAT.ACTCACCG	PM11	1.67000997066498
UNinjured_indrop_UC1_blood2_TAAGGCTC_AGCGTGGT_TGGCCTGT	PM11	1.80351996421814
WASP96hr_dropseq_PI24CIRC3_TCACAATGTTTA.3	PM11	1.87188994884491
injured_tenx_B2_GCTTGAAGTAAGAGGA	PM11	1.90569996833801
WASP96hr_dropseq_PI24CIRC3_TATATCTGGAAT.3	PM11	2.21012997627258
injured_tenx_B2_GTGTTAGAGCTACCGC	PM11	3.01639008522034
injured_tenx_B4_TGCCCTACACCAGATT	PM12	0.4932020008564
injured_tenx_B2_CCTATTAAGGGAAACA	PM12	0.610179007053375
injured_tenx_B4_TGAGCATCAGACAAAT	PM12	0.640879988670349
injured_tenx_B4_ACATCAGGTGCCTGGT	PM12	0.641261994838715
injured_tenx_B4_TAGTGGTAGCGCTTAT	PM12	0.663659989833832
injured_tenx_B4_TGGGCGTAGAAGATTC	PM12	0.687004029750824
injured_tenx_B4_CTCGAGGCAAGTTCTG	PM12	0.705470979213715
injured_tenx_B4_CGCTATCCAGTCCTTC	PM12	0.726764023303986
injured_tenx_B4_TCAGCTCTCTGCCAGG	PM12	0.756274998188019
injured_tenx_B4_CGTGAGCAGCCGATTT	PM12	0.908855974674225
injured_tenx_B4_GCACATAGTACCGTAT	PM12	0.945291996002197
injured_tenx_B2_TACGGGCAGATATGGT	PM12	0.955806970596313
injured_tenx_B4_TCACAAGTCCTGTACC	PM12	0.975857019424438
injured_tenx_B4_CTCAGAAGTTAAGACA	PM12	1.09423005580902
injured_tenx_B4_CGGACACTCTCTAAGG	PM12	1.18610000610352
injured_tenx_B4_GATCAGTGTCTCGTTC	PM12	1.21949994564056
injured_tenx_B4_GGGCACTAGTCAAGGC	PM12	1.23588001728058
injured_tenx_B4_TCCCGATGTCTGATCA	PM12	1.48085999488831
WASP96hr_dropseq_PI24CIRC1_ACTACAAGCTCT.1	non-hemo	0.393249988555908
WASP96hr_dropseq_PI24CIRC1_TAAAATACGCTG.1	non-hemo	0.548417985439301
WASP96hr_dropseq_PI24CIRC1_CGGGAGTAGATT.1	non-hemo	0.555543005466461

Cell Barcode	Cluster Name	FBgn0046687
WASP96hr_dropseq_PI24CIRC1_GTACACCCCACT.1	non-hemo	0.692247986793518
WASP96hr_dropseq_PI24CIRC1_TTTTCATTTTTC.1	non-hemo	0.693947970867157
WASP96hr_dropseq_PI24CIRC2_TATACAATCGTC.2	non-hemo	0.980517029762268
WASP96hr_dropseq_PI24CIRC1_GCGTCGCCGTAG.1	non-hemo	1.22457003593445
WASP96hr_dropseq_PI24CIRC2_AAGCTCTGAATT.2	non-hemo	1.68141996860504
injured_tenx_B4_TGCCAAAGTTGTCTTT	PM2	0.33263099193573
WASP96hr_dropseq_PI24CIRC1_GGTATAATCAAA.1	PM2	0.493699014186859
WASP96hr_dropseq_PI24CIRC2_GATGTGAGTTCT.2	PM2	0.504818975925446
WASP96hr_dropseq_PI24CIRC3_CCGTCTGAAGTA.3	PM2	0.510851979255676
WASP96hr_dropseq_PI24CIRC3_GTTTATCTCTTC.3	PM2	0.5171719789505
UNinjured_tenx_B3_GGGCACTAGAAGGGTA	PM2	0.534277021884918
WASP96hr_dropseq_PI24CIRC3_GGGCCCATTCCC.3	PM2	0.562178015708923
WASP96hr_dropseq_PI24CIRC3_CAACGAGAACTA.3	PM2	0.569077014923096
WASP96hr_dropseq_PI24CIRC1_CGGCTTTGAGGC.1	PM2	0.579896986484528
WASP96hr_dropseq_PI24CIRC3_CAGCCCCATTCC.3	PM2	0.606438994407654
WASP96hr_dropseq_PI24CIRC3_AAACCACCCTAC.3	PM2	0.622168004512787
WASP96hr_dropseq_PI24CIRC3_GGTCTATGTAGT.3	PM2	0.627003014087677
WASP96hr_dropseq_PI24CIRC1_AGAAGCGCTGCT.1	PM2	0.631092011928558
UNinjured_tenx_B3_CGTTAGATCCGAACGC	PM2	0.631668984889984
injured_tenx_B4_TCTCATACAAGCCATT	PM2	0.633448004722595
WASP96hr_dropseq_PI24CIRC3_AATCTGACTCAC.3	PM2	0.635236978530884
WASP96hr_dropseq_PI24CIRC3_AAGCCCCTATCC.3	PM2	0.654340028762817
WASP96hr_dropseq_PI24CIRC3_GTATAGTTCGGC.3	PM2	0.677236020565033
UNinjured_tenx_B3_CTTAACTGTAAGTGTA	PM2	0.686170995235443
WASP96hr_dropseq_PI24CIRC3_GTAGCTAGCACC.3	PM2	0.68641597032547
injured_tenx_B2_GACCAATTCTTTCCTC	PM2	0.706146001815796
WASP96hr_dropseq_PI24CIRC3_TCTTTAGCACGG.3	PM2	0.739920973777771
WASP96hr_dropseq_PI24CIRC3_ACAGACCATCGG.3	PM2	0.741702020168304
WASP96hr_dropseq_PI24CIRC3_CCGATATACGCA.3	PM2	0.741989970207214
injured_tenx_B4_ATGGGAGTCCGTTGTC	PM2	0.743490993976593
injured_tenx_B4_CGCGTTTTCACTATTC	PM2	0.761653006076813
WASP96hr_dropseq_PI24CIRC2_ACGCCTATTTTA.2	PM2	0.7639120221138
WASP96hr_dropseq_PI24CIRC3_GTTTCACGAGGA.3	PM2	0.772526025772095
WASP96hr_dropseq_PI24CIRC3_CTACACTTGGCA.3	PM2	0.776308000087738
WASP96hr_dropseq_PI24CIRC2_ATTTTGAGTTCT.2	PM2	0.779233992099762
WASP96hr_dropseq_PI24CIRC3_TGGGATTCTTCG.3	PM2	0.803911983966827
WASP96hr_dropseq_PI24CIRC3_ATATCGTGAAAC.3	PM2	0.809266984462738
WASP96hr_dropseq_PI24CIRC2_CAGTGCGATACA.2	PM2	0.809786021709442
WASP96hr_dropseq_PI24CIRC3_TCCTATGTTTCT.3	PM2	0.827530980110168
WASP96hr_dropseq_PI24CIRC3_GCCGGAACATCT.3	PM2	0.828329026699066
WASP96hr_dropseq_PI24CIRC3_TGCTCGGCTTCG.3	PM2	0.834994971752167
UNinjured_tenx_B3_GTAACGTGTATTACCG	PM2	0.837513983249664
UNinjured_tenx_B3_ACACCAAGTCTTTCAT	PM2	0.841248989105225
WASP96hr_dropseq_PI24CIRC3_ATCTACCCTTCT.3	PM2	0.849820017814636
WASP96hr_dropseq_PI24CIRC3_CCCCTTTGTTGG.3	PM2	0.857873976230621
WASP96hr_dropseq_PI24CIRC3_ATGCCCTCCGGG.3	PM2	0.863862991333008

Cell Barcode	Cluster Name	FBgn0046687
WASP96hr_dropseq_PI24CIRC3_CGTCCGCAGGGA.3	PM2	0.868815004825592
WASP96hr_dropseq_PI24CIRC3_GATCCCATTATA.3	PM2	0.869377017021179
WASP96hr_dropseq_PI24CIRC2_CGGGCGTATCTC.2	PM2	0.870908975601196
WASP96hr_dropseq_PI24CIRC3_TGTGTTCATGGA.3	PM2	0.88646399974823
WASP96hr_dropseq_PI24CIRC3_CGGTAGATAACT.3	PM2	0.887975990772247
injured_tenx_B4_TGCCAAACAGGTTTCA	PM2	0.90489399433136
injured_tenx_B4_TTGCCGTAGGCTAGCA	PM2	0.907001972198486
injured_tenx_B4_TGTTCCGGTGATAAAC	PM2	0.913033008575439
WASP96hr_dropseq_PI24CIRC3_TCATTGCCCCGT.3	PM2	0.913748025894165
injured_tenx_B4_CGGTTAAGTCTAGGTT	PM2	0.917702972888947
WASP96hr_dropseq_PI24CIRC3_CACGTGCGAGTT.3	PM2	0.919694006443024
WASP96hr_dropseq_PI24CIRC3_CCGCCACCTCCT.3	PM2	0.922151029109955
WASP96hr_dropseq_PI24CIRC3_AGGTCGCAAGGA.3	PM2	0.922424018383026
WASP96hr_dropseq_PI24CIRC3_GGGTCCCCGTCC.3	PM2	0.929418981075287
WASP96hr_dropseq_PI24CIRC1_CACAGCGCCTTG.1	PM2	0.943372011184692
UNinjured_tenx_B3_CGATGGCGTCTAGTCA	PM2	0.944139003753662
UNinjured_tenx_B3_CGCGTTTAGAACTGTA	PM2	0.946689009666443
UNinjured_tenx_B3_AGTAGTCGTACTCGCG	PM2	0.947510004043579
WASP96hr_dropseq_PI24CIRC3_TTAGATAGAACG.3	PM2	0.949545979499817
WASP96hr_dropseq_PI24CIRC3_CTCCCTGGTGCG.3	PM2	0.949837028980255
WASP96hr_dropseq_PI24CIRC3_CGTTGTGTCAGA.3	PM2	0.951981008052826
WASP96hr_dropseq_PI24CIRC3_TTTTATCCAGAG.3	PM2	0.95897102355957
UNinjured_tenx_B3_GCGCCAACAGCTGTAT	PM2	0.966880023479462
injured_indrop_PC1_blood2_CTTAATAG.CGTGCAAC.CGTGGATA	PM2	0.971141993999481
WASP96hr_dropseq_PI24CIRC3_GTGGCTCAGCCT.3	PM2	0.976216971874237
UNinjured_indrop_UC1_blood2_TAAGGCTC_AAACACTA_TCCTTATT	PM2	0.977198004722595
WASP96hr_dropseq_PI24CIRC3_ATCAGGTAAGCT.3	PM2	0.979476988315582
injured_tenx_B2_ATCACGACAAGTACCT	PM2	0.981455028057098
WASP96hr_dropseq_PI24CIRC3_GAGTACGGAAAC.3	PM2	0.988497972488403
UNinjured_tenx_B1_TGAGAGGAGATATGGT	PM2	0.993183016777039
WASP96hr_dropseq_PI24CIRC3_TCCCTATGGACG.3	PM2	0.998673021793365
UNinjured_tenx_B3_TGCCCATAGCTGATAA	PM2	1.00411999225616
injured_tenx_B4_CGATGGCTCAACACTG	PM2	1.00731003284454
UNinjured_indrop_UC1_blood2_TAAGGCTC_GGGAGATG_AATGTTTG	PM2	1.01521003246307
WASP96hr_dropseq_PI24CIRC3_GGGCTACACACC.3	PM2	1.01813995838165
WASP96hr_dropseq_PI24CIRC2_GTGCCTAGGTTG.2	PM2	1.02289998531342
WASP96hr_dropseq_PI24CIRC3_TGAGTACATTGA.3	PM2	1.02968001365662
injured_indrop_PC2_blood3_TCGCATAA.ACGTAAAC.TTTGGGAG	PM2	1.03750002384186
UNinjured_tenx_B3_CAGCAGCAGAGCTTCT	PM2	1.04199004173279
injured_tenx_B4_ACTGCTCAGACACGAC	PM2	1.05731999874115
injured_tenx_B4_GGGCACTCAATGTAAG	PM2	1.05915999412537
UNinjured_tenx_B1_AGACGTTCATACTACG	PM2	1.06560003757477
injured_tenx_B4_CGTCTACCATGGGACA	PM2	1.06634998321533
WASP96hr_dropseq_PI24CIRC3_GCGGGCGCTAGG.3	PM2	1.0691100358963
injured_indrop_PC1_blood2_CTTAATAG.TCGACTGC.CTTAGGTA	PM2	1.07824003696442
WASP96hr_dropseq_PI24CIRC3_ACTACGTTACCT.3	PM2	1.0789999961853

Cell Barcode	Cluster Name	FBgn0046687
WASP96hr_dropseq_PI24CIRC2_GACTGACCCTCG.2	PM2	1.08501994609833
WASP96hr_dropseq_PI24CIRC3_ATGGTAGCGCTG.3	PM2	1.08708000183105
injured_indrop_PC1_blood2_CTTAATAG.AGGTTCCC.GTCATATT	PM2	1.08714997768402
WASP96hr_dropseq_PI24CIRC3_GCAAAGCAGCTG.3	PM2	1.08780002593994
injured_tenx_B4_GCGCCAAAGACGCTTT	PM2	1.09396994113922
WASP96hr_dropseq_PI24CIRC3_TCCGTAGATTTC.3	PM2	1.09463000297546
WASP96hr_dropseq_PI24CIRC3_CCTCTGAAGGTC.3	PM2	1.0986100435257
WASP96hr_dropseq_PI24CIRC3_CTGGTAAATTAC.3	PM2	1.10169005393982
injured_tenx_B4_GGATGTTGTGTTGAGG	PM2	1.10558998584747
injured_tenx_B4_TACCTTACATCTCGCT	PM2	1.10871005058289
injured_tenx_B2_GTTTCTAGTGATGTGG	PM2	1.11486995220184
injured_tenx_B4_CAGAGAGAGGAATGGA	PM2	1.11556005477905
UNinjured_tenx_B3_GTGAAGGGTTTGTTTC	PM2	1.11707997322083
injured_tenx_B4_CCTACCAGTGCGGTAA	PM2	1.12110996246338
injured_tenx_B2_TTCGGTCCAAGACACG	PM2	1.12292003631592
injured_tenx_B4_CGGCTAGCACAACTGT	PM2	1.12799000740051
injured_tenx_B4_TGCCCTACAGCTGTTA	PM2	1.15824997425079
UNinjured_indrop_UC1_blood2_TAAGGCTC_ACGCTTAA_GCCAAGAT	PM2	1.16457998752594
injured_tenx_B2_AACTCTTCAGCTCGAC	PM2	1.16823995113373
WASP96hr_dropseq_PI24CIRC2_AAGCTATGCTCG.2	PM2	1.17693996429443
injured_tenx_B4_ACATGGTTCACGCATA	PM2	1.17887997627258
injured_indrop_PC1_blood2_CTTAATAG.CTACGGGA.TGACGGAC	PM2	1.18201005458832
injured_tenx_B4_TAAACCGGTCTGCCAG	PM2	1.18436002731323
injured_indrop_PC1_blood2_CTTAATAG.AAGTTGTC.TCCCGTAG	PM2	1.199049949646
injured_tenx_B4_TTCGAAGAGAAACGAG	PM2	1.20474004745483
injured_tenx_B4_ATCTGCCTCTTCATGT	PM2	1.20572996139526
injured_indrop_PC1_blood2_CTTAATAG.GGCTTTGC.TTAGTCCG	PM2	1.20868003368378
WASP96hr_dropseq_PI24CIRC2_ATGCCACCGCCT.2	PM2	1.22017002105713
injured_indrop_PC2_blood3_TCGCATAA.CCGGAAAT.CTTCTTCG	PM2	1.22525000572205
WASP96hr_dropseq_PI24CIRC3_GATCCTCTAAGC.3	PM2	1.23207998275757
WASP96hr_dropseq_PI24CIRC3_ACGTCGCGCCCA.3	PM2	1.23743999004364
UNinjured_tenx_B3_ATTACTCTCACTCTTA	PM2	1.23987996578217
injured_tenx_B4_CGGAGCTCACTGTCGG	PM2	1.24618995189667
injured_indrop_PC1_blood2_CTTAATAG.CTTTGGAC.GGTCCCTT	PM2	1.2509800195694
injured_tenx_B4_ATTACTCGTCCCTACT	PM2	1.25527000427246
injured_tenx_B4_AAGCCGCAGCGCCTTG	PM2	1.27684998512268
injured_indrop_PC1_blood2_CTTAATAG.AAAGAGGC.ATTTCCGG	PM2	1.2770299911499
injured_indrop_PC1_blood2_CTTAATAG.TCGCAATC.GTGTAACC	PM2	1.28076994419098
injured_indrop_PC1_blood2_CTTAATAG.ATCCGCAT.CAGATTAC	PM2	1.28605997562408
WASP96hr_dropseq_PI24CIRC3_GACCATCGGCCA.3	PM2	1.28718996047974
injured_tenx_B4_GTGTTAGTCCCATTAT	PM2	1.28756999969482
injured_indrop_PC1_blood2_CTTAATAG.AAACGTGA.ACTTCAAT	PM2	1.29387998580933
injured_indrop_PC1_blood2_CTTAATAG.AATATGAC.AAACTCGA	PM2	1.30414998531342
injured_tenx_B2_TCTGAGATCCTTCAAT	PM2	1.30807995796204
injured_indrop_PC2_blood3_TCGCATAA.ATGCATAT.CCCGAATG	PM2	1.30905997753143
UNinjured_tenx_B3_AAAGATGCAATGTTGC	PM2	1.32001996040344

Cell Barcode	Cluster Name	FBgn0046687
UNinjured_indrop_UC1_blood2_TAAGGCTC_ATGCATAT_GAGTTCAC	PM2	1.32042002677917
WASP96hr_dropseq_PI24CIRC2_CCCTCATGCTTC.2	PM2	1.32183003425598
WASP96hr_dropseq_PI24CIRC3_TGGTTAAGGAAG.3	PM2	1.32343995571136
injured_indrop_PC2_blood3_TCGCATAA.TAGATCAA.GGACTTCC	PM2	1.33385002613068
injured_tenx_B4_GATCGCGAGTAGGTGC	PM2	1.33509004116058
UNinjured_indrop_UC1_blood2_TAAGGCTC_CATGACGA_GTCAATAC	PM2	1.33653998374939
injured_tenx_B4_CGGACACTCGTGGTCG	PM2	1.33674001693726
injured_indrop_PC2_blood3_TCGCATAA.CTAACGCC.GAATTCGT	PM2	1.3490799665451
WASP96hr_dropseq_PI24CIRC3_ATTACTCGCCAC.3	PM2	1.35781002044678
WASP96hr_dropseq_PI24CIRC3_CACATCGATACG.3	PM2	1.35824000835419
injured_indrop_PC1_blood2_CTTAATAG.TAATCCAT.ACTAGCCA	PM2	1.35909998416901
UNinjured_tenx_B3_GTACTCCAGTGGACGT	PM2	1.36731994152069
injured_tenx_B2_TATTACCAGGTAAACT	PM2	1.37279999256134
injured_tenx_B2_GACTGCGAGTGTTGAA	PM2	1.39066004753113
injured_indrop_PC1_blood2_CTTAATAG.ACAGATAA.AACCCTTG	PM2	1.39339005947113
WASP96hr_dropseq_PI24CIRC2_TAACGGGACGGA.2	PM2	1.40418004989624
WASP96hr_dropseq_PI24CIRC2_GGGCCAAGTCTG.2	PM2	1.40767002105713
injured_indrop_PC1_blood2_CTTAATAG.GGAAGTCC.TGTTCCAG	PM2	1.40789997577667
injured_tenx_B4_GAGTCCGCAAAGCAAT	PM2	1.41118001937866
WASP96hr_dropseq_PI24CIRC3_AGGAGACCTCCA.3	PM2	1.41422998905182
UNinjured_indrop_UC1_blood2_TAAGGCTC_GCCTTGTG_TCTGTGGT	PM2	1.43241000175476
WASP96hr_dropseq_PI24CIRC3_ATTACTCGCCAA.3	PM2	1.43435001373291
WASP96hr_dropseq_PI24CIRC3_GAACTCAAGTAT.3	PM2	1.44877004623413
injured_tenx_B2_TTGAACGAGACTTTCG	PM2	1.4597100019455
injured_indrop_PC2_blood3_TCGCATAA.AATACATC.TGTAGTTT	PM2	1.46275997161865
injured_tenx_B2_TTCTCAATCTGTGCAA	PM2	1.48693001270294
WASP96hr_dropseq_PI24CIRC3_GAGCATAAAGAC.3	PM2	1.50548005104065
UNinjured_indrop_UC1_blood2_TAAGGCTC_AGGCAATA_AGTTTAGA	PM2	1.51247000694275
injured_tenx_B4_GCAGCCAAGTGTGGCA	PM2	1.52599000930786
injured_tenx_B4_AGTGAGGTCGAGGTAG	PM2	1.52683997154236
WASP96hr_dropseq_PI24CIRC3_CGAGCAAGGCCT.3	PM2	1.53922998905182
injured_tenx_B2_AGCTCTCGTAGCTCCG	PM2	1.53936994075775
WASP96hr_dropseq_PI24CIRC3_GCGCCTGCCCTT.3	PM2	1.55805003643036
injured_indrop_PC1_blood2_CTTAATAG.TCAAGGCG.AGAACGGG	PM2	1.57666003704071
injured_tenx_B4_CCATTCGGTTACAGAA	PM2	1.57696998119354
injured_indrop_PC2_blood3_TCGCATAA.CGATGGCA.GATTGCGA	PM2	1.57788002490997
WASP96hr_dropseq_PI24CIRC3_GGCAAGGTGACT.3	PM2	1.58772003650665
injured_indrop_PC1_blood2_CTTAATAG.GTGTCACC.TAGCTTAT	PM2	1.58896005153656
injured_indrop_PC2_blood3_TCGCATAA.ACATCTCG.GGGAACGA	PM2	1.59676003456116
injured_tenx_B2_TGCCCTAGTCAAGCGA	PM2	1.60084998607635
UNinjured_indrop_UC1_blood2_TAAGGCTC_GTGAACTC_GGTCCCTT	PM2	1.61618995666504
injured_tenx_B4_CATATGGTCAGTTCGA	PM2	1.62793004512787
injured_tenx_B2_CAACCAAGTCTCTTAT	PM2	1.63138997554779
injured_indrop_PC1_blood2_CTTAATAG.CCCTGTTT.CCCATAGC	PM2	1.63987994194031
injured_tenx_B4_CAAGATCGTTGACGTT	PM2	1.64711999893188
WASP96hr_dropseq_PI24CIRC3_GGTTTCTCGTCA.3	PM2	1.68630003929138

Cell Barcode	Cluster Name	FBgn0046687
WASP96hr_dropseq_PI24CIRC3_CGTTCCCCTTGA.3	PM2	1.68880999088287
injured_indrop_PC1_blood2_CTTAATAG.CTGTTTCC.GGGAAGGT	PM2	1.69459998607635
injured_tenx_B2_TGCTGCTTCGAATGCT	PM2	1.70519995689392
injured_indrop_PC2_blood3_TCGCATAA.GCATCACT.TGTAAAGG	PM2	1.72575998306274
injured_tenx_B4_AGCTCCTTCCGCGTTT	PM2	1.73032999038696
WASP96hr_dropseq_PI24CIRC3_TTGTCTTGACCA.3	PM2	1.74603998661041
injured_tenx_B2_GTCATTTCATTAGCCA	PM2	1.75958001613617
UNinjured_indrop_UC1_blood2_TAAGGCTC_CTCACATC_CGAGTCTG	PM2	1.85759997367859
WASP96hr_dropseq_PI24CIRC3_GAAACCCGGAGG.3	PM2	1.86448001861572
injured_tenx_B4_CAAGAAAAGGTGATAT	PM2	1.89433002471924
WASP96hr_dropseq_PI24CIRC2_AACATCGATAGN.2	PM2	1.89665997028351
WASP96hr_dropseq_PI24CIRC3_CGTGGGTTAGGC.3	PM2	1.98046004772186
WASP96hr_dropseq_PI24CIRC3_CGATAAGTAGTC.3	PM2	1.99294996261597
WASP96hr_dropseq_PI24CIRC3_CGCTGACCTACA.3	PM2	2.02196002006531
injured_indrop_PC1_blood2_CTTAATAG.AAACACGG.TTTAACAG	PM2	2.02480006217957
WASP96hr_dropseq_PI24CIRC1_CACCGCCTTACN.1	PM2	2.07703995704651
injured_indrop_PC2_blood3_TCGCATAA.AATCGAAG.TTCGTTCC	PM2	2.09308004379272
injured_tenx_B2_GCGCAACTCGCCAAAT	PM2	2.16722011566162
WASP96hr_dropseq_PI24CIRC1_CGTAGCTCTCGA.1	LM1	0.268141001462936
WASP96hr_dropseq_PI24CIRC1_GAATGTCCGTTT.1	LM1	0.268705993890762
WASP96hr_dropseq_PI24CIRC1_TAGATGGAGAAT.1	LM1	0.282373994588852
WASP96hr_dropseq_PI24CIRC1_CGCCAGAATCTC.1	LM1	0.284583002328873
WASP96hr_dropseq_PI24CIRC1_GATACCGATGCG.1	LM1	0.296548992395401
WASP96hr_dropseq_PI24CIRC1_GGTTGAACAAGT.1	LM1	0.306068986654282
WASP96hr_dropseq_PI24CIRC1_GGCCGGGCGTGG.1	LM1	0.306295990943909
WASP96hr_dropseq_PI24CIRC2_CACGCGCTTAGT.2	LM1	0.332130014896393
WASP96hr_dropseq_PI24CIRC1_CTATGATTGTCT.1	LM1	0.333135008811951
WASP96hr_dropseq_PI24CIRC2_GTTACGAATGGT.2	LM1	0.337078988552094
WASP96hr_dropseq_PI24CIRC2_TTGAATCCGACT.2	LM1	0.344873011112213
WASP96hr_dropseq_PI24CIRC1_GCAGTTTATCGT.1	LM1	0.350490003824234
WASP96hr_dropseq_PI24CIRC2_CACCCCTTGGTC.2	LM1	0.356229990720749
WASP96hr_dropseq_PI24CIRC1_AGCGTTCATTGC.1	LM1	0.361198008060455
WASP96hr_dropseq_PI24CIRC2_TAATTATTGGAG.2	LM1	0.369152992963791
UNinjured_tenx_B3_AAATGCCTCGTTGCCT	LM1	0.37473601102829
WASP96hr_dropseq_PI24CIRC2_GATAGTAATAGG.2	LM1	0.389387995004654
WASP96hr_dropseq_PI24CIRC1_TATGAGCCCGAG.1	LM1	0.389494985342026
WASP96hr_dropseq_PI24CIRC2_GCTAGCATAGCC.2	LM1	0.392031997442245
WASP96hr_dropseq_PI24CIRC2_TTCTAGGGGAGA.2	LM1	0.40760999917984
WASP96hr_dropseq_PI24CIRC2_CGTATCGCCCCT.2	LM1	0.413801997900009
WASP96hr_dropseq_PI24CIRC1_AAGTCTAGTCGC.1	LM1	0.417641997337341
WASP96hr_dropseq_PI24CIRC2_GTACCGGGTCAG.2	LM1	0.420403003692627
WASP96hr_dropseq_PI24CIRC2_GCTCATTTTCGT.2	LM1	0.424825996160507
WASP96hr_dropseq_PI24CIRC2_GCCTCCCCCAGG.2	LM1	0.427222013473511
WASP96hr_dropseq_PI24CIRC1_TGGTTCTGAATC.1	LM1	0.435227990150452
WASP96hr_dropseq_PI24CIRC3_CTGGAATTTAAC.3	LM1	0.441000998020172
WASP96hr_dropseq_PI24CIRC2_CACTCGGTAAAA.2	LM1	0.449259012937546

Cell Barcode	Cluster Name	FBgn0046687
WASP96hr_dropseq_PI24CIRC2_CCTAACACGAAA.2	LM1	0.449362009763718
WASP96hr_dropseq_PI24CIRC1_CGGTACGGGATC.1	LM1	0.450596988201141
WASP96hr_dropseq_PI24CIRC2_GCGCCCCCGAGG.2	LM1	0.451030999422073
WASP96hr_dropseq_PI24CIRC2_TCCGTAGTTATC.2	LM1	0.451341986656189
WASP96hr_dropseq_PI24CIRC1_CGTAAGCCGCAG.1	LM1	0.456243991851807
WASP96hr_dropseq_PI24CIRC2_CCGAGGATCCGC.2	LM1	0.459701001644135
WASP96hr_dropseq_PI24CIRC2_CAACCTTTGCCT.2	LM1	0.465671986341476
UNinjured_tenx_B3_GACTACATCGTCTGAA	LM1	0.467000991106033
WASP96hr_dropseq_PI24CIRC2_GCCATCCGTGTA.2	LM1	0.474105000495911
WASP96hr_dropseq_PI24CIRC2_AAATATCGTTAC.2	LM1	0.484860002994537
WASP96hr_dropseq_PI24CIRC3_CAGCGTTTATCT.3	LM1	0.485772013664246
WASP96hr_dropseq_PI24CIRC2_CGTACGGGACCC.2	LM1	0.496250003576279
WASP96hr_dropseq_PI24CIRC2_TTGTACGGGTAC.2	LM1	0.497965008020401
WASP96hr_dropseq_PI24CIRC1_GAGCTGATCGTC.1	LM1	0.504260003566742
WASP96hr_dropseq_PI24CIRC2_GTTTGATTGGTT.2	LM1	0.507908999919891
WASP96hr_dropseq_PI24CIRC2_TAAATGAGACGA.2	LM1	0.509841024875641
WASP96hr_dropseq_PI24CIRC3_TCGGCCCTTTCT.3	LM1	0.510052978992462
WASP96hr_dropseq_PI24CIRC2_CAGAGTATTTAT.2	LM1	0.51087898015976
WASP96hr_dropseq_PI24CIRC1_AGGCTTAACGCT.1	LM1	0.511359989643097
WASP96hr_dropseq_PI24CIRC2_TCGATGGAGCGT.2	LM1	0.515563011169434
WASP96hr_dropseq_PI24CIRC2_CCATCAGCTCAG.2	LM1	0.516680002212524
WASP96hr_dropseq_PI24CIRC2_CAATGCTCGAAT.2	LM1	0.518020987510681
WASP96hr_dropseq_PI24CIRC2_GGACCTTGTGGA.2	LM1	0.518020987510681
WASP96hr_dropseq_PI24CIRC3_TTGTCTTCCGTC.3	LM1	0.520169973373413
WASP96hr_dropseq_PI24CIRC1_ATACGGAGAAGG.1	LM1	0.524046003818512
WASP96hr_dropseq_PI24CIRC1_GGCCCGCTCTGC.1	LM1	0.526219010353088
injured_tenx_B4_ATAGACCCAGACAAAT	LM1	0.542501986026764
WASP96hr_dropseq_PI24CIRC2_CAGAGGAGGAAT.2	LM1	0.549189984798431
WASP96hr_dropseq_PI24CIRC3_CAGTGCTTTCCG.3	LM1	0.551519989967346
WASP96hr_dropseq_PI24CIRC2_CCATTCAACTTT.2	LM1	0.553696990013123
WASP96hr_dropseq_PI24CIRC3_GCGTCGGTGAGT.3	LM1	0.570378005504608
WASP96hr_dropseq_PI24CIRC2_TCGACCGTAGTT.2	LM1	0.573503971099854
WASP96hr_dropseq_PI24CIRC2_GGTATGAGCACT.2	LM1	0.575402975082397
WASP96hr_dropseq_PI24CIRC2_GGAATTGATATG.2	LM1	0.581422984600067
WASP96hr_dropseq_PI24CIRC2_TCTAAGGCTCCC.2	LM1	0.582746982574463
WASP96hr_dropseq_PI24CIRC2_GCGTTTCTCGAT.2	LM1	0.583552002906799
WASP96hr_dropseq_PI24CIRC2_AGTGATGTGTCA.2	LM1	0.593388020992279
WASP96hr_dropseq_PI24CIRC2_GGCCCGGGACGG.2	LM1	0.593460023403168
WASP96hr_dropseq_PI24CIRC3_TCGTTTATGCTA.3	LM1	0.59799200296402
WASP96hr_dropseq_PI24CIRC2_TTACGTCCTCTT.2	LM1	0.598029017448425
WASP96hr_dropseq_PI24CIRC2_TCTCTAATCCGC.2	LM1	0.598249971866608
injured_tenx_B4_TATCTCATCTTACCTA	LM1	0.600992023944855
WASP96hr_dropseq_PI24CIRC2_AAGTCTGCAGCT.2	LM1	0.615018010139465
WASP96hr_dropseq_PI24CIRC2_ACGTCCGTGAGA.2	LM1	0.622888028621674
injured_tenx_B4_CCTACCAAGCGTCAAG	LM1	0.626515984535217
WASP96hr_dropseq_PI24CIRC3_ACCGACCTTCAC.3	LM1	0.626922011375427

Cell Barcode	Cluster Name	FBgn0046687
WASP96hr_dropseq_PI24CIRC3_GTTTAATCGTAA.3	LM1	0.631339013576508
WASP96hr_dropseq_PI24CIRC3_ACATAATCTTTC.3	LM1	0.632412016391754
WASP96hr_dropseq_PI24CIRC2_CATTCATGTAGA.2	LM1	0.633364975452423
WASP96hr_dropseq_PI24CIRC3_GACTGGCGGGTA.3	LM1	0.634070992469788
WASP96hr_dropseq_PI24CIRC2_GCTGCCAAAGAG.2	LM1	0.635946989059448
WASP96hr_dropseq_PI24CIRC2_CTGCCAGTATCG.2	LM1	0.639649987220764
WASP96hr_dropseq_PI24CIRC2_AGAATGAGGTGA.2	LM1	0.642114996910095
WASP96hr_dropseq_PI24CIRC3_TCATTCCCATAG.3	LM1	0.644684970378876
WASP96hr_dropseq_PI24CIRC1_ATTTCCGCTACN.1	LM1	0.645806014537811
injured_tenx_B2_AAGACCTTCTGTTTGT	LM1	0.647885024547577
WASP96hr_dropseq_PI24CIRC2_CACTCCGCTCCA.2	LM1	0.650328993797302
WASP96hr_dropseq_PI24CIRC2_GGCCTGTTCTTC.2	LM1	0.653918981552124
WASP96hr_dropseq_PI24CIRC2_GGGCTGCAAAGA.2	LM1	0.654762029647827
WASP96hr_dropseq_PI24CIRC1_CCGTAACTTTGT.1	LM1	0.65507298707962
WASP96hr_dropseq_PI24CIRC2_TAGGGGGCACGG.2	LM1	0.657729983329773
WASP96hr_dropseq_PI24CIRC2_TGGCCTACAGCA.2	LM1	0.67044597864151
WASP96hr_dropseq_PI24CIRC2_CCTCGCTGATAA.2	LM1	0.678620994091034
injured_tenx_B2_CTACATTGTCATATGC	LM1	0.680298984050751
WASP96hr_dropseq_PI24CIRC2_ACTCTGTGGCCA.2	LM1	0.68140697479248
WASP96hr_dropseq_PI24CIRC2_CCAGTTACAAAG.2	LM1	0.68791401386261
WASP96hr_dropseq_PI24CIRC3_TTTAGGTCCTAT.3	LM1	0.688825011253357
WASP96hr_dropseq_PI24CIRC3_GCGTATTATTAC.3	LM1	0.693346977233887
WASP96hr_dropseq_PI24CIRC2_AAGTTGATGAGC.2	LM1	0.693798005580902
WASP96hr_dropseq_PI24CIRC2_CCGGTCGGTGTG.2	LM1	0.695152997970581
WASP96hr_dropseq_PI24CIRC2_GTCTCTCGTCGA.2	LM1	0.70134699344635
WASP96hr_dropseq_PI24CIRC3_GCCCAATACGCG.3	LM1	0.702835977077484
WASP96hr_dropseq_PI24CIRC1_ATCCAGCGCACT.1	LM1	0.704572975635529
WASP96hr_dropseq_PI24CIRC2_TAAGTACCCCGA.2	LM1	0.705833971500397
WASP96hr_dropseq_PI24CIRC2_AGGCGGAAAATG.2	LM1	0.712758004665375
WASP96hr_dropseq_PI24CIRC2_GAGCAGTTAACT.2	LM1	0.714067995548248
WASP96hr_dropseq_PI24CIRC2_TCACCCCGGGCG.2	LM1	0.718961000442505
WASP96hr_dropseq_PI24CIRC2_GGCCATATAGCT.2	LM1	0.719878971576691
WASP96hr_dropseq_PI24CIRC2_GCCTTCGGTCTA.2	LM1	0.721777975559235
WASP96hr_dropseq_PI24CIRC2_AAGTCATGATAT.2	LM1	0.722868025302887
WASP96hr_dropseq_PI24CIRC2_GGTGAGAGAGGA.2	LM1	0.723742008209229
WASP96hr_dropseq_PI24CIRC2_TTATGTAAATCA.2	LM1	0.724819004535675
WASP96hr_dropseq_PI24CIRC3_CCGATTGTCGCG.3	LM1	0.732999980449677
WASP96hr_dropseq_PI24CIRC2_ACTCCGGTAGCG.2	LM1	0.742219984531403
WASP96hr_dropseq_PI24CIRC3_CCAGTTCGTCTG.3	LM1	0.744534015655518
UNinjured_tenx_B1_CCTACCACACATCCAA	LM1	0.751568019390106
WASP96hr_dropseq_PI24CIRC1_TGGCACCCGGTT.1	LM1	0.751824975013733
WASP96hr_dropseq_PI24CIRC2_CGTTCGTGATTT.2	LM1	0.753467977046967
WASP96hr_dropseq_PI24CIRC2_ATCCCAAGCAGG.2	LM1	0.754957973957062
WASP96hr_dropseq_PI24CIRC2_AGCGAGTACGAG.2	LM1	0.7622309923172
WASP96hr_dropseq_PI24CIRC1_CGGCATTGCACG.1	LM1	0.763504028320312
injured_tenx_B4_GGCGTGTGTGTTGAGG	LM1	0.769403994083405

Cell Barcode	Cluster Name	FBgn0046687
injured_tenx_B4_AGAGCTTGTTAAGACA	LM1	0.785870015621185
WASP96hr_dropseq_PI24CIRC3_ATTTTAAAGGCT.3	LM1	0.78600001335144
WASP96hr_dropseq_PI24CIRC2_CTGTGCATAAAA.2	LM1	0.786324977874756
WASP96hr_dropseq_PI24CIRC3_ATCCGTTTGGGT.3	LM1	0.788740992546082
UNinjured_tenx_B3_TGTATTCCAATAGCGG	LM1	0.788806974887848
UNinjured_indrop_UC1_blood2_TAAGGCTC_ATGCGGAG_GTCCATGT	LM1	0.78913402557373
WASP96hr_dropseq_PI24CIRC3_TATCATGTTGGC.3	LM1	0.79084300994873
WASP96hr_dropseq_PI24CIRC2_GTCCAAGAAGGC.2	LM1	0.791073977947235
WASP96hr_dropseq_PI24CIRC3_TGAGCTAGGTCT.3	LM1	0.791172981262207
WASP96hr_dropseq_PI24CIRC2_ACGTCAGACGAG.2	LM1	0.793951988220215
WASP96hr_dropseq_PI24CIRC2_CGTAACAATAGT.2	LM1	0.794283986091614
injured_tenx_B2_GGTGCGTTCGTTTGCC	LM1	0.794550001621246
WASP96hr_dropseq_PI24CIRC3_ATTTTTGGCCTT.3	LM1	0.800450026988983
WASP96hr_dropseq_PI24CIRC3_CCTTCATAAGTA.3	LM1	0.807748973369598
WASP96hr_dropseq_PI24CIRC3_CCATCCGTACCT.3	LM1	0.81554102897644
WASP96hr_dropseq_PI24CIRC2_GCTCTCTCGTCC.2	LM1	0.815822005271912
WASP96hr_dropseq_PI24CIRC2_CATGGGACTAGC.2	LM1	0.81786698102951
WASP96hr_dropseq_PI24CIRC3_TGTCTTTTTAGT.3	LM1	0.81971001625061
WASP96hr_dropseq_PI24CIRC3_CGAGTAGTCACC.3	LM1	0.81999397277832
injured_tenx_B4_CAGGTGCTCTTTAGTC	LM1	0.82956600189209
WASP96hr_dropseq_PI24CIRC1_TGGCCCAGGCTT.1	LM1	0.835763990879059
UNinjured_tenx_B3_AGAATAGAGACTTGAA	LM1	0.836326003074646
WASP96hr_dropseq_PI24CIRC2_GCCTCGAACTCT.2	LM1	0.841587007045746
WASP96hr_dropseq_PI24CIRC3_AAGCATTAGCTT.3	LM1	0.848213016986847
WASP96hr_dropseq_PI24CIRC2_CTCCGGTCTGTG.2	LM1	0.851664006710052
injured_tenx_B4_CACATAGTCACCGTAA	LM1	0.852627992630005
WASP96hr_dropseq_PI24CIRC1_CATTTGTGCGCC.1	LM1	0.853672027587891
WASP96hr_dropseq_PI24CIRC3_TGATGGCGAATA.3	LM1	0.856779992580414
WASP96hr_dropseq_PI24CIRC2_CGCTAAGCATAG.2	LM1	0.857249021530151
WASP96hr_dropseq_PI24CIRC1_ATTTCCGCTAGN.1	LM1	0.857756972312927
WASP96hr_dropseq_PI24CIRC3_TCAGTTTGCCGC.3	LM1	0.859520018100739
WASP96hr_dropseq_PI24CIRC2_CAACAAAATTTG.2	LM1	0.862793028354645
WASP96hr_dropseq_PI24CIRC2_GACGGAGCAATA.2	LM1	0.864022016525269
WASP96hr_dropseq_PI24CIRC1_GACTTCTCGAGG.1	LM1	0.870364010334015
WASP96hr_dropseq_PI24CIRC2_TTCTTTCGTTCG.2	LM1	0.872067987918854
WASP96hr_dropseq_PI24CIRC3_GAGCTGAGGCCA.3	LM1	0.874477982521057
WASP96hr_dropseq_PI24CIRC3_TTAACGACTACG.3	LM1	0.880716979503632
WASP96hr_dropseq_PI24CIRC2_ACCGCCAATGAA.2	LM1	0.881711006164551
WASP96hr_dropseq_PI24CIRC2_AGAATTGTCGCT.2	LM1	0.882748007774353
WASP96hr_dropseq_PI24CIRC2_CGGTCCCCCAAT.2	LM1	0.883247017860413
WASP96hr_dropseq_PI24CIRC2_TCTACTGCGCCT.2	LM1	0.893355011940002
injured_tenx_B2_CGATGGCTCGTGGACC	LM1	0.897683024406433
injured_tenx_B4_TTAACTCTCGTCCAGG	LM1	0.898545026779175
WASP96hr_dropseq_PI24CIRC1_GTTCAGGTGAAG.1	LM1	0.899524986743927
injured_indrop_PC1_blood2_CTTAATAG.CTGAGCGT.AACCCTTG	LM1	0.902099013328552
WASP96hr_dropseq_PI24CIRC3_GTGATATCCATA.3	LM1	0.909298002719879

Cell Barcode	Cluster Name	FBgn0046687
WASP96hr_dropseq_PI24CIRC3_GCGCTGCACGAC.3	LM1	0.911695003509521
WASP96hr_dropseq_PI24CIRC2_CCATGCCCCTAG.2	LM1	0.93530398607254
injured_tenx_B2_GCGCAGTTCCTAGGGC	LM1	0.936716973781586
UNinjured_tenx_B3_TTTCCTCCATGGAATA	LM1	0.937471985816956
WASP96hr_dropseq_PI24CIRC3_ACACGTCAGTGC.3	LM1	0.938703000545502
WASP96hr_dropseq_PI24CIRC2_GAAGCGGCGTTG.2	LM1	0.940887987613678
WASP96hr_dropseq_PI24CIRC3_CCCAGGCCCCTC.3	LM1	0.949156999588013
WASP96hr_dropseq_PI24CIRC1_GTGTCGCCCCCA.1	LM1	0.950689017772675
WASP96hr_dropseq_PI24CIRC3_AGACGTCCGCAT.3	LM1	0.950908005237579
WASP96hr_dropseq_PI24CIRC1_TGAGATCCTTCT.1	LM1	0.95362001657486
WASP96hr_dropseq_PI24CIRC2_CGCCACTGTAGG.2	LM1	0.958773016929626
WASP96hr_dropseq_PI24CIRC2_ATGTGCTTATGC.2	LM1	0.966441988945007
injured_tenx_B4_TATGCCCTCTTTACGT	LM1	0.968802988529205
WASP96hr_dropseq_PI24CIRC3_GTACGTACCACC.3	LM1	0.970530986785889
WASP96hr_dropseq_PI24CIRC1_TCGGTTGGGACC.1	LM1	0.970887005329132
injured_indrop_PC2_blood3_TCGCATAA.TGGAAAGC.AGTTTAGA	LM1	0.971243977546692
WASP96hr_dropseq_PI24CIRC3_CGTGGATCGATA.3	LM1	0.973083019256592
UNinjured_indrop_UC1_blood2_TAAGGCTC_CCGTAGCT_ATGACCGA	LM1	0.974622011184692
WASP96hr_dropseq_PI24CIRC2_CCGTAGCCCGTA.2	LM1	0.975102007389069
UNinjured_tenx_B3_GCTGCAGAGATATGGT	LM1	0.976526975631714
injured_tenx_B4_GGGCATCTCAGCATGT	LM1	0.977818012237549
WASP96hr_dropseq_PI24CIRC3_GAATGCAGCACT.3	LM1	0.978542983531952
WASP96hr_dropseq_PI24CIRC3_TCTTGCGCATAG.3	LM1	0.979476988315582
WASP96hr_dropseq_PI24CIRC2_TCCTTACTATCT.2	LM1	0.979927003383636
WASP96hr_dropseq_PI24CIRC2_ATACTCTCCGGC.2	LM1	0.982707977294922
WASP96hr_dropseq_PI24CIRC2_AGGAATTTACTC.2	LM1	0.98770397901535
injured_tenx_B2_GTAACTGGTCACACGC	LM1	0.991580009460449
injured_tenx_B4_CGGCTAGCATTCGACA	LM1	0.996836006641388
injured_tenx_B4_TAAACCGAGATTACCC	LM1	0.999215006828308
WASP96hr_dropseq_PI24CIRC2_AGCGCGCGCTGG.2	LM1	1.00390005111694
WASP96hr_dropseq_PI24CIRC2_CTCACCCAGACC.2	LM1	1.00689995288849
WASP96hr_dropseq_PI24CIRC2_AGGACCCGTAAT.2	LM1	1.00864005088806
injured_tenx_B2_ACCTTTACAAGTAGTA	LM1	1.01330995559692
injured_tenx_B2_TGTCCCATCCAGAAGG	LM1	1.01655995845795
injured_indrop_PC2_blood3_TCGCATAA.CCGGAAAT.AAGCTTCT	LM1	1.017009973526
WASP96hr_dropseq_PI24CIRC2_CGATTGATTGAC.2	LM1	1.0202499628067
WASP96hr_dropseq_PI24CIRC3_AACCATTAAACT.3	LM1	1.02438998222351
injured_tenx_B4_GTACTCCGTCCGAATT	LM1	1.02864003181458
WASP96hr_dropseq_PI24CIRC2_GTACACGGTTTG.2	LM1	1.03058004379272
WASP96hr_dropseq_PI24CIRC2_GTCAGACCTGCA.2	LM1	1.0306099653244
WASP96hr_dropseq_PI24CIRC3_TCGCTATTGATA.3	LM1	1.03119003772736
WASP96hr_dropseq_PI24CIRC3_GTACGCACAGCA.3	LM1	1.03159999847412
WASP96hr_dropseq_PI24CIRC2_GGTTTCTTGCGT.2	LM1	1.0393899679184
WASP96hr_dropseq_PI24CIRC3_ATCCAAACCTCG.3	LM1	1.04128003120422
injured_tenx_B4_CTCTAATCATGGGACA	LM1	1.04544997215271
WASP96hr_dropseq_PI24CIRC3_CTAGGTTGATGT.3	LM1	1.04857003688812

Cell Barcode	Cluster Name	FBgn0046687
WASP96hr_dropseq_PI24CIRC2_GATATGCCATTC.2	LM1	1.04953002929688
injured_tenx_B4_AAGACCTAGAACTGTA	LM1	1.05025994777679
UNinjured_tenx_B3_ACACCAACACATGACT	LM1	1.05499005317688
UNinjured_tenx_B3_GATCAGTGTCAGGACA	LM1	1.05572998523712
WASP96hr_dropseq_PI24CIRC3_GGAATAAAACGC.3	LM1	1.05719995498657
injured_indrop_PC1_blood2_CTTAATAG.GGAGTAAG.TCACCGAG	LM1	1.06397998332977
WASP96hr_dropseq_PI24CIRC3_CTTTCGCGAGCG.3	LM1	1.06417000293732
injured_tenx_B4_GTGGGTCAGGACGAAA	LM1	1.06809997558594
WASP96hr_dropseq_PI24CIRC3_CGTGTCTAATTG.3	LM1	1.07035994529724
WASP96hr_dropseq_PI24CIRC2_CTAAAAAAATTT.2	LM1	1.0791300535202
injured_tenx_B4_AAGACCTCAGAAGCAC	LM1	1.07990002632141
UNinjured_tenx_B3_CATTATCCAAGTAGTA	LM1	1.08092999458313
injured_indrop_PC1_blood2_CTTAATAG.GATGTGGC.GTTCTGTT	LM1	1.08209002017975
injured_indrop_PC1_blood2_CTTAATAG.AATGTATG.TTCCGAGT	LM1	1.08467996120453
WASP96hr_dropseq_PI24CIRC2_TAATCCGCGATT.2	LM1	1.08597004413605
injured_tenx_B4_GCGCAGTCACTTAAGC	LM1	1.09159994125366
WASP96hr_dropseq_PI24CIRC2_AATCAGGTTAGC.2	LM1	1.09790003299713
WASP96hr_dropseq_PI24CIRC3_CAAGGACCCGGG.3	LM1	1.09900999069214
WASP96hr_dropseq_PI24CIRC2_TCAGAAGCCCCC.2	LM1	1.10095000267029
injured_indrop_PC1_blood2_CTTAATAG.TCATACCA.AGAACGGG	LM1	1.10222995281219
WASP96hr_dropseq_PI24CIRC1_AGGATCAGCGTG.1	LM1	1.10395002365112
injured_indrop_PC1_blood2_CTTAATAG.ACTCGGAA.TAGTGTTT	LM1	1.10967004299164
WASP96hr_dropseq_PI24CIRC3_ATACACAGAGTC.3	LM1	1.10967004299164
WASP96hr_dropseq_PI24CIRC3_AAAACCCCCTTT.3	LM1	1.11102998256683
injured_tenx_B4_GCGCCAAAGCTGAACG	LM1	1.11532998085022
WASP96hr_dropseq_PI24CIRC2_CCAGGACTTCGA.2	LM1	1.12189996242523
WASP96hr_dropseq_PI24CIRC2_CTAGTATGAGAC.2	LM1	1.1251699924469
WASP96hr_dropseq_PI24CIRC2_TTCGAAGCGCCT.2	LM1	1.12699997425079
WASP96hr_dropseq_PI24CIRC2_GGTCAAGGGGAA.2	LM1	1.13039004802704
WASP96hr_dropseq_PI24CIRC3_GAACACCCAGTT.3	LM1	1.13209998607635
WASP96hr_dropseq_PI24CIRC2_CCCACACTCGGT.2	LM1	1.13447999954224
WASP96hr_dropseq_PI24CIRC3_AAATTACGTTGT.3	LM1	1.13516998291016
WASP96hr_dropseq_PI24CIRC2_GGTGTAGAAGCA.2	LM1	1.13926994800568
injured_indrop_PC2_blood3_TCGCATAA.GGTTAGGG.CACAAGGC	LM1	1.14188003540039
WASP96hr_dropseq_PI24CIRC2_CACCGGCTACCG.2	LM1	1.14523005485535
WASP96hr_dropseq_PI24CIRC2_CTCACCCTGCTA.2	LM1	1.14625000953674
injured_indrop_PC2_blood3_TCGCATAA.GCGTATTC.TAGTCTAG	LM1	1.15022003650665
WASP96hr_dropseq_PI24CIRC2_CTAGTGCGAAGG.2	LM1	1.15222001075745
WASP96hr_dropseq_PI24CIRC2_GACCCACAATAG.2	LM1	1.15363001823425
WASP96hr_dropseq_PI24CIRC2_CAATTGAGTCCA.2	LM1	1.1557400226593
injured_indrop_PC1_blood2_CTTAATAG.GGCGACAA.GACAACTT	LM1	1.15661001205444
injured_tenx_B4_CTAACTTAGATGTGTA	LM1	1.15900003910065
WASP96hr_dropseq_PI24CIRC2_GGTAACATCGAC.2	LM1	1.161749958992
injured_tenx_B4_AGCGTCGTCCACTCCA	LM1	1.16216003894806
WASP96hr_dropseq_PI24CIRC2_GAGCAGGCAGTA.2	LM1	1.16700994968414
injured_indrop_PC2_blood3_TCGCATAA.GCCAACAT.TCATCCTT	LM1	1.16946005821228

Cell Barcode	Cluster Name	FBgn0046687
injured_indrop_PC1_blood2_CTTAATAG.ACCCGATT.GAAAGACC	LM1	1.16975998878479
injured_indrop_PC1_blood2_CTTAATAG.AAGGCGCT.TTCACATA	LM1	1.17022001743317
injured_tenx_B2_AACTTTCTCAGCGATT	LM1	1.17221999168396
injured_indrop_PC2_blood3_TCGCATAA.AGGTTCCC.CGCTAGTC	LM1	1.17376005649567
WASP96hr_dropseq_PI24CIRC2_ACCCCCTGGTTG.2	LM1	1.17593002319336
WASP96hr_dropseq_PI24CIRC2_GATCTCCACCTA.2	LM1	1.17935001850128
WASP96hr_dropseq_PI24CIRC3_TGATACTCTGAC.3	LM1	1.17957997322083
WASP96hr_dropseq_PI24CIRC2_GATTGGCAGAAT.2	LM1	1.18066000938416
WASP96hr_dropseq_PI24CIRC3_TCAACTCCCATA.3	LM1	1.18154001235962
injured_tenx_B2_CGCTGGACATTCGACA	LM1	1.18201005458832
WASP96hr_dropseq_PI24CIRC1_GCGCCTAGCCGC.1	LM1	1.18322002887726
injured_tenx_B4_AAGACCTGTGATAAAC	LM1	1.18388998508453
WASP96hr_dropseq_PI24CIRC2_GGTTGCGACGTA.2	LM1	1.1866500377655
injured_tenx_B4_AATCGGTGTCGCATAT	LM1	1.18752002716064
injured_indrop_PC2_blood3_TCGCATAA.TGAGAGCG.CAGATTAC	LM1	1.19086003303528
injured_indrop_PC1_blood2_CTTAATAG.TACCTAAG.GTTTACGT	LM1	1.19196999073029
injured_indrop_PC2_blood3_TCGCATAA.GAGGAGTG.ACCCACGA	LM1	1.19872999191284
injured_indrop_PC1_blood2_CTTAATAG.AGCGTGGT.GCCTCTTT	LM1	1.20181000232697
injured_tenx_B4_TGATTTCGTACATGTC	LM1	1.20278000831604
injured_indrop_PC1_blood2_CTTAATAG.TATTAGCG.TCTCACTT	LM1	1.20295000076294
WASP96hr_dropseq_PI24CIRC2_GGTGCATGTTGT.2	LM1	1.20555996894836
injured_tenx_B4_AGACGTTGTGGCGAAT	LM1	1.21332001686096
injured_indrop_PC2_blood3_TCGCATAA.CCTACGCT.CTTTAATC	LM1	1.21397995948792
injured_tenx_B2_GCAATCAGTTGGTTTG	LM1	1.21564996242523
injured_tenx_B4_TTGCGTCTCCCACTTG	LM1	1.22541999816895
injured_tenx_B4_CATGGCGGTATCGCAT	LM1	1.22780001163483
UNinjured_tenx_B3_AAAGATGTCTTTACAC	LM1	1.23207998275757
WASP96hr_dropseq_PI24CIRC1_TAGGAGACCTCG.1	LM1	1.23311996459961
WASP96hr_dropseq_PI24CIRC2_GAGATGGTTGCG.2	LM1	1.23795998096466
WASP96hr_dropseq_PI24CIRC2_AGCCTCTTGATC.2	LM1	1.23847997188568
WASP96hr_dropseq_PI24CIRC3_CTTCGCGTAAGA.3	LM1	1.23917996883392
WASP96hr_dropseq_PI24CIRC2_ATTAGTTGGGGG.2	LM1	1.24390995502472
WASP96hr_dropseq_PI24CIRC2_TAATTTGACTAN.2	LM1	1.2476099729538
WASP96hr_dropseq_PI24CIRC2_CAGCGCTGAAGT.2	LM1	1.24893999099731
UNinjured_tenx_B3_GTAACTGGTTCCTCCA	LM1	1.24937999248505
injured_indrop_PC1_blood2_CTTAATAG.AGTGCTTC.CGAACGTA	LM1	1.25045001506805
injured_indrop_PC1_blood2_CTTAATAG.ACTAGAGC.CACAACAG	LM1	1.25760996341705
injured_indrop_PC1_blood2_CTTAATAG.AAACTACA.GCCGGATT	LM1	1.25868999958038
WASP96hr_dropseq_PI24CIRC1_TCCATTTAATTA.1	LM1	1.26180005073547
WASP96hr_dropseq_PI24CIRC2_GTTCGCGATAGN.2	LM1	1.26195001602173
WASP96hr_dropseq_PI24CIRC3_TTGACTGCCGTG.3	LM1	1.26449000835419
WASP96hr_dropseq_PI24CIRC2_AGATTGTTTGCA.2	LM1	1.26601004600525
WASP96hr_dropseq_PI24CIRC2_TACGGGTGACCT.2	LM1	1.26696002483368
injured_indrop_PC1_blood2_CTTAATAG.TAGGTACG.TGATAACA	LM1	1.27368998527527
injured_indrop_PC2_blood3_TCGCATAA.AGGACACA.CCCGACTT	LM1	1.28266000747681
injured_indrop_PC1_blood2_CTTAATAG.AAGGCGCT.TGCCATCG	LM1	1.28284001350403

Cell Barcode	Cluster Name	FBgn0046687
WASP96hr_dropseq_PI24CIRC3_AGACTGCTGACA.3	LM1	1.2853000164032
WASP96hr_dropseq_PI24CIRC1_GGTGGAGCCCTG.1	LM1	1.28745996952057
WASP96hr_dropseq_PI24CIRC2_CAACTAGAGTTT.2	LM1	1.29014003276825
UNinjured_tenx_B1_CAGCATAAGATGAGAG	LM1	1.29330003261566
WASP96hr_dropseq_PI24CIRC3_GCGTTACGCTCG.3	LM1	1.29445004463196
injured_tenx_B4_GTATTCTCAGAAGCAC	LM1	1.29560995101929
WASP96hr_dropseq_PI24CIRC2_GGGAATTCGGAC.2	LM1	1.29609000682831
WASP96hr_dropseq_PI24CIRC2_CGTCATCGTCGG.2	LM1	1.2994099855423
UNinjured_tenx_B3_GGACAAGCAAGGACTG	LM1	1.30414998531342
WASP96hr_dropseq_PI24CIRC2_TCATGGCTCGCT.2	LM1	1.30445003509521
UNinjured_indrop_UC1_blood2_TAAGGCTC_ACCACAGA_AGGCAGTT	LM1	1.30611002445221
UNinjured_tenx_B3_CGTTAGAGTGTAAGTA	LM1	1.30867004394531
injured_indrop_PC1_blood2_CTTAATAG.AGGGACTG.TTCCGAGT	LM1	1.31063997745514
injured_tenx_B4_CATTATCCAAAGTGCG	LM1	1.3135199546814
injured_indrop_PC1_blood2_CTTAATAG.CGTCGAAT.CCCGACTT	LM1	1.31761002540588
UNinjured_tenx_B3_ACGGGTCCAGGTGCCT	LM1	1.31781005859375
WASP96hr_dropseq_PI24CIRC1_CTATTGGTCTCC.1	LM1	1.3188099861145
WASP96hr_dropseq_PI24CIRC2_TTTCTCCCCAGN.2	LM1	1.32263994216919
WASP96hr_dropseq_PI24CIRC2_GCGACTACCAGC.2	LM1	1.32263994216919
WASP96hr_dropseq_PI24CIRC2_AAGTTCACGTTG.2	LM1	1.32304000854492
WASP96hr_dropseq_PI24CIRC2_CGCCACACCTTA.2	LM1	1.3257600069046
WASP96hr_dropseq_PI24CIRC3_CTGGATTGTGTG.3	LM1	1.32661998271942
injured_indrop_PC2_blood3_TCGCATAA.GGCAAGGT.ATCTTTGT	LM1	1.32872998714447
injured_indrop_PC2_blood3_TCGCATAA.AATAAGGA.GTATGAGT	LM1	1.33077001571655
injured_tenx_B4_CCGGTAGGTTCCATGA	LM1	1.33262002468109
injured_indrop_PC1_blood2_CTTAATAG.AGTCACAA.TCGGTTTA	LM1	1.33467996120453
UNinjured_indrop_UC1_blood2_TAAGGCTC_CGGCTGTA_GGTGACAC	LM1	1.33467996120453
injured_tenx_B2_AAAGCAATCCTCCTAG	LM1	1.34151995182037
injured_tenx_B2_TCATTTGTCGTTACGA	LM1	1.34318995475769
injured_tenx_B4_CACACCTTCACAGGCC	LM1	1.34423995018005
WASP96hr_dropseq_PI24CIRC3_ACAGTTTGCGTA.3	LM1	1.34696996212006
injured_tenx_B4_GCGCAGTCATTGGTAC	LM1	1.35035002231598
injured_tenx_B2_ACGGGTCTCGACAGCC	LM1	1.35225999355316
WASP96hr_dropseq_PI24CIRC1_GCTCAAGAACGT.1	LM1	1.35264003276825
WASP96hr_dropseq_PI24CIRC2_TCTGAGACCATN.2	LM1	1.35566997528076
injured_indrop_PC1_blood2_CTTAATAG.AAACCGCC.CTGTTCTT	LM1	1.35898995399475
injured_indrop_PC1_blood2_CTTAATAG.AGGAGTCG.CTACGAGC	LM1	1.36189997196198
injured_indrop_PC1_blood2_CTTAATAG.ACGGAGCA.GCAACCTG	LM1	1.36427998542786
WASP96hr_dropseq_PI24CIRC3_AGCTGTCACTAG.3	LM1	1.36696004867554
WASP96hr_dropseq_PI24CIRC2_GGCGAGGACATT.2	LM1	1.37015998363495
WASP96hr_dropseq_PI24CIRC2_ATTCCTTCGCTG.2	LM1	1.37638998031616
injured_indrop_PC1_blood2_CTTAATAG.GGCTATAA.AAGTAATC	LM1	1.37876999378204
injured_indrop_PC1_blood2_CTTAATAG.CCATATGA.TAAGACGG	LM1	1.37966001033783
UNinjured_indrop_UC1_blood2_TAAGGCTC_CGGACTAA_GAACGATT	LM1	1.38411998748779
WASP96hr_dropseq_PI24CIRC2_CCGATCGGATGA.2	LM1	1.3846800327301
WASP96hr_dropseq_PI24CIRC2_CGGCCCGCATGA.2	LM1	1.38473999500275

Cell Barcode	Cluster Name	FBgn0046687
injured_tenx_B2_CCCTCCTCATGGAATA	LM1	1.3850200176239
WASP96hr_dropseq_PI24CIRC3_ACTAAGGTCCCT.3	LM1	1.38547003269196
injured_indrop_PC1_blood2_CTTAATAG.GTCTTCCT.ATATGCAT	LM1	1.38952994346619
WASP96hr_dropseq_PI24CIRC2_GACGCCATGTCT.2	LM1	1.39474999904633
injured_indrop_PC1_blood2_CTTAATAG.TGAATCCT.TTAGGACC	LM1	1.39886999130249
WASP96hr_dropseq_PI24CIRC3_CTCGGTACGGTG.3	LM1	1.40118002891541
WASP96hr_dropseq_PI24CIRC2_TAATTTGACTGN.2	LM1	1.40394997596741
injured_tenx_B4_CCTTCGATCCAAACTG	LM1	1.40418004989624
WASP96hr_dropseq_PI24CIRC2_AGCACATACAAA.2	LM1	1.40481996536255
injured_indrop_PC1_blood2_CTTAATAG.GTCTTCCT.CAACGTGG	LM1	1.4104700088501
WASP96hr_dropseq_PI24CIRC2_CACTCAGGGAAC.2	LM1	1.41118001937866
UNinjured_indrop_UC1_blood2_TAAGGCTC_AACGCCAA_ACTAGGAT	LM1	1.41140997409821
WASP96hr_dropseq_PI24CIRC2_GGTTGCCTGCGC.2	LM1	1.41375994682312
WASP96hr_dropseq_PI24CIRC3_ATAACTCATTTA.3	LM1	1.41565001010895
injured_indrop_PC1_blood2_CTTAATAG.ATCCTAGT.TGGTATGA	LM1	1.41612005233765
injured_indrop_PC1_blood2_CTTAATAG.CCGTCTTA.TTATAGCC	LM1	1.41707003116608
injured_indrop_PC2_blood3_TCGCATAA.ATGTGCCG.ACTGAGTT	LM1	1.41895997524261
WASP96hr_dropseq_PI24CIRC3_GCGCAATGCTAT.3	LM1	1.41919994354248
WASP96hr_dropseq_PI24CIRC2_AGTAAATGATCG.2	LM1	1.41979002952576
injured_indrop_PC1_blood2_CTTAATAG.AGACCTCA.AATGACCC	LM1	1.42039000988007
injured_indrop_PC1_blood2_CTTAATAG.CTGAAGGG.TCCAGTCC	LM1	1.4213399887085
injured_tenx_B2_GTTCGGGGTACAAGTA	LM1	1.42541003227234
injured_tenx_B2_GGGTTGCGTTTAGCTG	LM1	1.42830002307892
UNinjured_tenx_B1_GCACTCTCAGCTTAAC	LM1	1.42853999137878
injured_indrop_PC2_blood3_TCGCATAA.TCCCTGGA.GGATAAAG	LM1	1.43435001373291
WASP96hr_dropseq_PI24CIRC3_CGATAGACCACT.3	LM1	1.4354499578476
injured_tenx_B4_CATCAGAGTCTGATCA	LM1	1.43728005886078
WASP96hr_dropseq_PI24CIRC2_GGTGGAGGGCCT.2	LM1	1.43842995166779
injured_indrop_PC1_blood2_CTTAATAG.ACGGGTTA.TACTTGTG	LM1	1.43998003005981
injured_indrop_PC2_blood3_TCGCATAA.CTGCGATG.CTTTCTTT	LM1	1.44096004962921
injured_indrop_PC1_blood2_CTTAATAG.GGCTTTGC.GACAACTT	LM1	1.44342994689941
WASP96hr_dropseq_PI24CIRC2_TAATTTGACTTN.2	LM1	1.44714999198914
injured_tenx_B4_GCTTCCAGTCGCATCG	LM1	1.45013999938965
injured_tenx_B2_GACTACACATTGGCGC	LM1	1.45414996147156
injured_indrop_PC1_blood2_CTTAATAG.TAATGTGG.CACTCCTC	LM1	1.45414996147156
WASP96hr_dropseq_PI24CIRC2_GGATTCTCAATG.2	LM1	1.45561003684998
injured_indrop_PC2_blood3_TCGCATAA.AGCCATCT.TGCCATCG	LM1	1.46098005771637
injured_tenx_B4_TAAGCGTTCATATCGG	LM1	1.46556997299194
injured_indrop_PC1_blood2_CTTAATAG.GAGCGGAA.TGATGCCC	LM1	1.47329998016357
injured_tenx_B4_CTTGGCTGTGATAAAC	LM1	1.47459995746613
injured_indrop_PC1_blood2_CTTAATAG.GGGATTAC.GTACCTTG	LM1	1.47720003128052
injured_indrop_PC1_blood2_CTTAATAG.ATACACCC.GTTACCGC	LM1	1.47798001766205
injured_indrop_PC2_blood3_TCGCATAA.ACGACGAC.GCTCTAGT	LM1	1.48296999931335
injured_indrop_PC1_blood2_CTTAATAG.TCCTATAT.AATGAATG	LM1	1.48533999919891
WASP96hr_dropseq_PI24CIRC2_TTCGCGCCTAAC.2	LM1	1.4864000082016
injured_indrop_PC2_blood3_TCGCATAA.GCCTGGTA.TTTAACAG	LM1	1.49680995941162

Cell Barcode	Cluster Name	FBgn0046687
WASP96hr_dropseq_PI24CIRC2_TCTATTTGCCTC.2	LM1	1.49816000461578
WASP96hr_dropseq_PI24CIRC3_TTATGTAATACA.3	LM1	1.49951004981995
injured_indrop_PC2_blood3_TCGCATAA.CGTTTCGT.GTTTGTTT	LM1	1.50466001033783
injured_tenx_B4_CATGGCGTCCAAGTAC	LM1	1.50574994087219
WASP96hr_dropseq_PI24CIRC2_GCTACACCAGAT.2	LM1	1.50601994991302
injured_indrop_PC1_blood2_CTTAATAG.ATATGGGT.CGTGGATA	LM1	1.50616002082825
injured_indrop_PC2_blood3_TCGCATAA.GACTAGCG.TAACCATC	LM1	1.50739002227783
UNinjured_indrop_UC1_blood2_TAAGGCTC_GCAAACTG_CGGACAAC	LM1	1.50794005393982
injured_tenx_B4_CAACCAAAGCGCCTTG	LM1	1.50986003875732
injured_tenx_B4_AATCCAGGTAGAAGGA	LM1	1.51040995121002
injured_tenx_B4_CACAGGCGTAAACACA	LM1	1.51496005058289
WASP96hr_dropseq_PI24CIRC3_ATACATCGCTCC.3	LM1	1.5223400592804
WASP96hr_dropseq_PI24CIRC2_ATCGCATTCCCA.2	LM1	1.52417004108429
WASP96hr_dropseq_PI24CIRC2_AATGGATGGCAA.2	LM1	1.52705001831055
injured_tenx_B4_TTAACTCTCGTGACAT	LM1	1.52769005298615
WASP96hr_dropseq_PI24CIRC2_CCGATGCAGATG.2	LM1	1.53051996231079
injured_tenx_B4_CCAGCGACACTTACGA	LM1	1.53735995292664
UNinjured_indrop_UC1_blood2_TAAGGCTC_GATTAAAG_ATGAAGCA	LM1	1.54167997837067
WASP96hr_dropseq_PI24CIRC2_ACGGGCCGCTAA.2	LM1	1.54870998859406
injured_indrop_PC2_blood3_TCGCATAA.CTGAAGGG.ATTGGGCC	LM1	1.54952001571655
injured_tenx_B4_AGAATAGCAAGGTGTG	LM1	1.55185997486115
injured_indrop_PC2_blood3_TCGCATAA.ACACCAAG.AGGATTCA	LM1	1.55953001976013
injured_indrop_PC1_blood2_CTTAATAG.CTCTATAG.CGGGCTTT	LM1	1.56728994846344
injured_indrop_PC1_blood2_CTTAATAG.ACGAATTC.CGTATCGG	LM1	1.56939005851746
WASP96hr_dropseq_PI24CIRC2_CCGGAGATGCAA.2	LM1	1.57271003723145
WASP96hr_dropseq_PI24CIRC2_TCTGAGACCACN.2	LM1	1.57635998725891
injured_indrop_PC2_blood3_TCGCATAA.AGCTTCGA.CCGCTGTT	LM1	1.57910001277924
injured_tenx_B4_CAGTAACTCAAGCCTA	LM1	1.58062994480133
injured_indrop_PC1_blood2_CTTAATAG.AAGTATTG.CAGTCCCT	LM1	1.58340001106262
injured_indrop_PC1_blood2_CTTAATAG.CTCCCAAA.ACGGTAGC	LM1	1.58896005153656
WASP96hr_dropseq_PI24CIRC2_AACGAAAGGAAC.2	LM1	1.58947002887726
WASP96hr_dropseq_PI24CIRC3_AGAGGTAGTAGA.3	LM1	1.60847997665405
injured_indrop_PC1_blood2_CTTAATAG.AAACGATC.GTGGATGG	LM1	1.6088000535965
WASP96hr_dropseq_PI24CIRC2_GTTCGCGATACN.2	LM1	1.63287997245789
injured_indrop_PC2_blood3_TCGCATAA.GTCAGACC.TTGTTTAC	LM1	1.63920998573303
injured_indrop_PC2_blood3_TCGCATAA.AAGGATGA.GCACCTCT	LM1	1.64289999008179
injured_indrop_PC1_blood2_CTTAATAG.GACACAAA.TGTACACG	LM1	1.64356994628906
injured_tenx_B4_GAGTCCGCATTCCTGC	LM1	1.64716994762421
WASP96hr_dropseq_PI24CIRC3_CTCCTAATGTCC.3	LM1	1.64932000637054
injured_indrop_PC2_blood3_TCGCATAA.CCCTCTTG.CCAACCGT	LM1	1.65409004688263
injured_indrop_PC1_blood2_CTTAATAG.AGAGGTGC.CGGGTAGT	LM1	1.65821003913879
injured_indrop_PC1_blood2_CTTAATAG.CAGACTCG.GCCGGATT	LM1	1.66096997261047
injured_tenx_B2_CTCGGAGGTCGAGATG	LM1	1.66165995597839
injured_indrop_PC1_blood2_CTTAATAG.AGAAACCA.CGTGGATA	LM1	1.67105996608734
injured_tenx_B4_GAACGGACAACGATCT	LM1	1.69532001018524
WASP96hr_dropseq_PI24CIRC2_TTATGAGTATAA.2	LM1	1.69814002513885

Cell Barcode	Cluster Name	FBgn0046687
injured_indrop_PC2_blood3_TCGCATAA.GGGAGATG.CCCTTGGT	LM1	1.70483005046844
WASP96hr_dropseq_PI24CIRC3_CCAGTTTGAGGG.3	LM1	1.70483005046844
injured_indrop_PC2_blood3_TCGCATAA.GAATCCCA.AGACACCA	LM1	1.70594000816345
injured_indrop_PC1_blood2_CTTAATAG.ACATCTCG.CATCTCCC	LM1	1.71447002887726
UNinjured_indrop_UC1_blood2_TAAGGCTC_CGTGTGTT_AAACTGGT	LM1	1.71709001064301
injured_tenx_B2_GTCTCGTTCGCAAGCC	LM1	1.72500002384186
injured_tenx_B4_CATGCCTGTGCGCTTG	LM1	1.73646998405457
injured_indrop_PC1_blood2_CTTAATAG.CTGTCGCA.CTGGTGCA	LM1	1.75165998935699
injured_indrop_PC1_blood2_CTTAATAG.GTTGTCCG.ATCCGCTA	LM1	1.75224995613098
injured_indrop_PC1_blood2_CTTAATAG.TACCTAAG.GTTGCACG	LM1	1.75838994979858
WASP96hr_dropseq_PI24CIRC2_TGGTAATTTTAT.2	LM1	1.77163004875183
injured_tenx_B4_ATTACTCCAAACTGCT	LM1	1.77691996097565
injured_indrop_PC1_blood2_CTTAATAG.GCTAAGTT.GACAGATA	LM1	1.78946995735168
injured_tenx_B2_ACACCCTAGAAGGGTA	LM1	1.81419003009796
WASP96hr_dropseq_PI24CIRC2_CTGTAGTAAATA.2	LM1	1.82196998596191
injured_tenx_B4_AGGGAGTCAAGCGTAG	LM1	1.82386004924774
WASP96hr_dropseq_PI24CIRC2_GATAAAGCCTAA.2	LM1	1.8254599571228
injured_tenx_B2_ACATCAGCATACCATG	LM1	1.82808005809784
injured_indrop_PC1_blood2_CTTAATAG.TAGATCAA.AATGTTTG	LM1	1.83402001857758
injured_indrop_PC2_blood3_TCGCATAA.ACCACAGA.TTCCGAGT	LM1	1.84268999099731
injured_tenx_B2_ACTGAGTAGCCGATTT	LM1	1.84403002262115
WASP96hr_dropseq_PI24CIRC2_ATGCGCATTAGG.2	LM1	1.84469997882843
injured_tenx_B4_CGCGGTATCTGGTGTA	LM1	1.85169005393982
injured_tenx_B2_GTGTGCGAGATAGCAT	LM1	1.85576999187469
WASP96hr_dropseq_PI24CIRC3_GCCCTTGAGACG.3	LM1	1.8589700460434
WASP96hr_dropseq_PI24CIRC2_ACTCATGATCCN.2	LM1	1.8637900352478
injured_indrop_PC1_blood2_CTTAATAG.CACAACCT.CTACTTGT	LM1	1.8677099943161
injured_tenx_B4_GCTCTGTTCTCTTATG	LM1	1.87328004837036
WASP96hr_dropseq_PI24CIRC3_CAGTCCAAGGCT.3	LM1	1.88526999950409
injured_indrop_PC1_blood2_CTTAATAG.GTCCTTGC.GACAACTT	LM1	1.9025399684906
injured_indrop_PC1_blood2_CTTAATAG.TGCTTCAT.TCTGTGGT	LM1	1.91402995586395
WASP96hr_dropseq_PI24CIRC3_TTCTCCTGTATG.3	LM1	1.91624999046326
injured_indrop_PC2_blood3_TCGCATAA.ATGTGCCG.CGCCTTGA	LM1	1.92997002601624
WASP96hr_dropseq_PI24CIRC2_AATCGTATCTCT.2	LM1	1.93654000759125
UNinjured_tenx_B1_TGTATTCCACAAGACG	LM1	1.96290004253387
injured_tenx_B2_CTCGGGAGTCTTGTCC	LM1	1.96501004695892
injured_tenx_B4_CGGTTAATCACGATGT	LM1	1.97335994243622
injured_tenx_B2_TGCTGCTCAAGGACTG	LM1	2.02196002006531
injured_tenx_B2_CTGCTGTTCTTGAGGT	LM1	2.05892992019653
injured_tenx_B2_CTTTGCGTCGTCGTTC	LM1	2.06281995773315
injured_indrop_PC1_blood2_CTTAATAG.CTGTTTCC.AGTGAAAG	LM1	2.07470011711121
injured_indrop_PC2_blood3_TCGCATAA.GGCGACAA.GCTTTCCA	LM1	2.07611989974976
injured_tenx_B2_ATCACGACAAGGTGTG	LM1	2.07979011535645
injured_indrop_PC2_blood3_TCGCATAA.AACTGCCT.GAGGCTGA	LM1	2.08316993713379
WASP96hr_dropseq_PI24CIRC3_AATAGAGCCCTG.3	LM1	2.08656001091003
injured_indrop_PC2_blood3_TCGCATAA.CGTGCAAC.CTTTAATC	LM1	2.09506011009216

Cell Barcode	Cluster Name	FBgn0046687
injured_indrop_PC1_blood2_CTTAATAG.ACTCGGAA.GATGTATT	LM1	2.09650993347168
WASP96hr_dropseq_PI24CIRC2_TAATTCTTGGAG.2	LM1	2.10279989242554
injured_tenx_B4_GATCGCGTCGTTTATC	LM1	2.12954998016357
injured_tenx_B4_CGGTTAATCACAAACC	LM1	2.17202997207642
UNinjured_indrop_UC2_blood3_TAAGGCTC_TACGTTCG_AATGAATG	LM1	2.19226002693176
injured_indrop_PC1_blood2_CTTAATAG.AATGTATG.TCCAGTCC	LM1	2.19343996047974
injured_tenx_B2_AAAGCAAGTGTGAATA	LM1	2.22214007377625
UNinjured_tenx_B1_TAAACCGAGCCTCGTG	LM1	2.23135995864868
injured_tenx_B4_TACTCGCAGCGTCAAG	LM1	2.30510997772217
injured_indrop_PC1_blood2_CTTAATAG.AGGCCGAA.GAGAGTAT	LM1	2.31656002998352
injured_indrop_PC1_blood2_CTTAATAG.GTGGTGCT.AGCTACGG	LM1	2.39902997016907
WASP96hr_dropseq_PI24CIRC2_GCGACTACCAGT.2	LM1	2.42812991142273
WASP96hr_dropseq_PI24CIRC3_AATTAGTTGGCT.3	LM1	2.4379301071167
injured_indrop_PC2_blood3_TCGCATAA.AGAATCAA.CGTAGAAG	LM1	2.48751997947693
injured_indrop_PC1_blood2_CTTAATAG.ATAAGCTA.CGTAGAAG	LM1	2.62140989303589
WASP96hr_dropseq_PI24CIRC2_TCGCCTGAATAC.2	LM1	2.80360007286072
injured_indrop_PC1_blood2_CTTAATAG.TGGTTAAC.CTAGATTG	LM1	2.92847990989685
injured_tenx_B4_CATGCCTGTCTTGATG	LM1	3.29675006866455
WASP96hr_dropseq_PI24CIRC2_GATAGGCCAATG.2	PM3	0.459035992622375
WASP96hr_dropseq_PI24CIRC2_ACATGGGTATCC.2	PM3	0.471666991710663
WASP96hr_dropseq_PI24CIRC2_GCCTGACATTCA.2	PM3	0.507408976554871
WASP96hr_dropseq_PI24CIRC2_GCACCACTGCAC.2	PM3	0.508834004402161
WASP96hr_dropseq_PI24CIRC2_CTAGCAGCCTCC.2	PM3	0.515834987163544
WASP96hr_dropseq_PI24CIRC2_AAGTAGGCAGCG.2	PM3	0.536273002624512
WASP96hr_dropseq_PI24CIRC2_ATCTATAGGAAC.2	PM3	0.541087985038757
WASP96hr_dropseq_PI24CIRC2_GCAGATCATCAA.2	PM3	0.5434690117836
WASP96hr_dropseq_PI24CIRC2_ATGGTGTTTTAG.2	PM3	0.557353973388672
WASP96hr_dropseq_PI24CIRC2_TATTGCAACATT.2	PM3	0.559240996837616
WASP96hr_dropseq_PI24CIRC2_ATAGACTTTGAT.2	PM3	0.564752995967865
WASP96hr_dropseq_PI24CIRC2_GAGGGACAGTTA.2	PM3	0.56834602355957
WASP96hr_dropseq_PI24CIRC2_TTATATACGTCC.2	PM3	0.58236300945282
WASP96hr_dropseq_PI24CIRC2_ACCCAACACACG.2	PM3	0.583446979522705
WASP96hr_dropseq_PI24CIRC2_GGGTTTGGGTTT.2	PM3	0.586687028408051
WASP96hr_dropseq_PI24CIRC2_TCCGACACGTTT.2	PM3	0.586934983730316
WASP96hr_dropseq_PI24CIRC2_CAGCTTATTGCA.2	PM3	0.592410027980804
WASP96hr_dropseq_PI24CIRC2_GTCCAGACCCTG.2	PM3	0.603872001171112
WASP96hr_dropseq_PI24CIRC2_TAAGGGCAGTGC.2	PM3	0.627530992031097
WASP96hr_dropseq_PI24CIRC2_ATTCATATCAGC.2	PM3	0.648711979389191
WASP96hr_dropseq_PI24CIRC2_CAGTGCTTCCGC.2	PM3	0.652967989444733
WASP96hr_dropseq_PI24CIRC2_ACTGGGCCACTT.2	PM3	0.65701299905777
WASP96hr_dropseq_PI24CIRC2_GCCATCTCTAGA.2	PM3	0.662245988845825
WASP96hr_dropseq_PI24CIRC2_CGAATGTTCCGA.2	PM3	0.664116978645325
WASP96hr_dropseq_PI24CIRC2_CGTCCATGAGCG.2	PM3	0.669003009796143
WASP96hr_dropseq_PI24CIRC2_CTTCTTATTGGA.2	PM3	0.671240985393524
WASP96hr_dropseq_PI24CIRC2_AGGTTAAGCAGT.2	PM3	0.676093995571136
WASP96hr_dropseq_PI24CIRC2_CTGACGATGATT.2	PM3	0.677379012107849

Cell Barcode	Cluster Name	FBgn0046687
WASP96hr_dropseq_PI24CIRC2_AACTCACGCATG.2	PM3	0.678286015987396
WASP96hr_dropseq_PI24CIRC2_GAGACTATCTAG.2	PM3	0.683295011520386
WASP96hr_dropseq_PI24CIRC2_TTCTCATCTGTG.2	PM3	0.685730993747711
WASP96hr_dropseq_PI24CIRC2_GCTCCCCTGATT.2	PM3	0.687593996524811
WASP96hr_dropseq_PI24CIRC2_GATACGGGGAGC.2	PM3	0.691850006580353
WASP96hr_dropseq_PI24CIRC2_CTACAGTTCCCG.2	PM3	0.696060001850128
WASP96hr_dropseq_PI24CIRC2_AGGTTGTGGAAG.2	PM3	0.701295971870422
WASP96hr_dropseq_PI24CIRC2_ACCATCCGGACA.2	PM3	0.702835977077484
WASP96hr_dropseq_PI24CIRC2_CCTTTTATTGGG.2	PM3	0.707552015781403
WASP96hr_dropseq_PI24CIRC2_TTTTTCCATGGC.2	PM3	0.716916978359222
WASP96hr_dropseq_PI24CIRC2_TCTCGAGAAGGA.2	PM3	0.717613995075226
WASP96hr_dropseq_PI24CIRC2_CTCGCCGGCGAC.2	PM3	0.729812026023865
WASP96hr_dropseq_PI24CIRC2_GCGGGTTAGGTT.2	PM3	0.73565000295639
WASP96hr_dropseq_PI24CIRC2_AGCGCACCCGCT.2	PM3	0.739920973777771
WASP96hr_dropseq_PI24CIRC2_ACCGATGGTTCC.2	PM3	0.740839004516602
injured_tenx_B4_CAAGTTGCAGCTGCAC	PM3	0.743085980415344
WASP96hr_dropseq_PI24CIRC2_TGCTGTCCCATG.2	PM3	0.745697021484375
WASP96hr_dropseq_PI24CIRC2_CGACTGGCATGA.2	PM3	0.749796986579895
WASP96hr_dropseq_PI24CIRC2_GAGTCCGCGCCT.2	PM3	0.751390993595123
WASP96hr_dropseq_PI24CIRC2_ACGAGACAAGAT.2	PM3	0.76080197095871
WASP96hr_dropseq_PI24CIRC2_GGGTCGCACCGA.2	PM3	0.762506008148193
WASP96hr_dropseq_PI24CIRC2_GTACTGCGAGGT.2	PM3	0.763055980205536
WASP96hr_dropseq_PI24CIRC2_TCCCGCGTAGAC.2	PM3	0.765507996082306
WASP96hr_dropseq_PI24CIRC2_GAACGGTCTTGA.2	PM3	0.767728984355927
WASP96hr_dropseq_PI24CIRC2_TCTGATGCAATG.2	PM3	0.769342005252838
WASP96hr_dropseq_PI24CIRC2_GTTTGGAGATAA.2	PM3	0.776498019695282
WASP96hr_dropseq_PI24CIRC2_AACCTGCCCCCT.2	PM3	0.784960985183716
WASP96hr_dropseq_PI24CIRC2_TGTTCGAATGGA.2	PM3	0.785870015621185
WASP96hr_dropseq_PI24CIRC2_TCGTGCCATGCT.2	PM3	0.78913402557373
WASP96hr_dropseq_PI24CIRC2_ACGCCACACTCG.2	PM3	0.79635101556778
WASP96hr_dropseq_PI24CIRC2_TTAAAACAGGGG.2	PM3	0.796886026859283
WASP96hr_dropseq_PI24CIRC2_GGAGAGATTATC.2	PM3	0.811034023761749
WASP96hr_dropseq_PI24CIRC2_GTCACTCGCTGA.2	PM3	0.818503975868225
WASP96hr_dropseq_PI24CIRC2_CGAAGGGTTTAC.2	PM3	0.819496989250183
WASP96hr_dropseq_PI24CIRC2_CTGGGGAACTGT.2	PM3	0.824427008628845
WASP96hr_dropseq_PI24CIRC2_GGGCAGATAGAA.2	PM3	0.824931025505066
WASP96hr_dropseq_PI24CIRC2_GAGATTACGGCT.2	PM3	0.827603995800018
injured_tenx_B2_AAGCCGCGTCTTGATG	PM3	0.831318020820618
WASP96hr_dropseq_PI24CIRC2_ATAAAAGGTGGT.2	PM3	0.840874016284943
WASP96hr_dropseq_PI24CIRC2_GGCAATGTTACG.2	PM3	0.863545000553131
WASP96hr_dropseq_PI24CIRC2_GTCCCGGGGAGC.2	PM3	0.865851998329163
WASP96hr_dropseq_PI24CIRC2_ACATCTGTTTCT.2	PM3	0.867610991001129
WASP96hr_dropseq_PI24CIRC2_AACCCTCGGTTG.2	PM3	0.874071002006531
injured_tenx_B2_ACACCAACAGTACACT	PM3	0.893653988838196
injured_tenx_B2_TCAGGTAGTATTCTCT	PM3	0.894550979137421
WASP96hr_dropseq_PI24CIRC2_TGGTCAACAAGT.2	PM3	0.903581976890564

Cell Barcode	Cluster Name	FBgn0046687
WASP96hr_dropseq_PI24CIRC2_TGTTTCGTCAGT.2	PM3	0.929883003234863
WASP96hr_dropseq_PI24CIRC2_GTGTGTGTGTCC.2	PM3	0.930347979068756
WASP96hr_dropseq_PI24CIRC2_TGACTGCACTCC.2	PM3	0.932771980762482
WASP96hr_dropseq_PI24CIRC2_CTCCATTCGTTA.2	PM3	0.936528027057648
WASP96hr_dropseq_PI24CIRC2_TTTCGTTAGTTT.2	PM3	0.936716973781586
WASP96hr_dropseq_PI24CIRC2_ACGTGATTAAGA.2	PM3	0.939652025699615
WASP96hr_dropseq_PI24CIRC2_GGGGTTGGGTGT.2	PM3	0.943754971027374
WASP96hr_dropseq_PI24CIRC3_GCTCTCGTTGCT.3	PM3	0.948768973350525
WASP96hr_dropseq_PI24CIRC2_TGCCTCATCTCG.2	PM3	0.95042097568512
WASP96hr_dropseq_PI24CIRC3_CCGACGCGTCTG.3	PM3	0.950614988803864
WASP96hr_dropseq_PI24CIRC2_CTCCGAGTCCGT.2	PM3	0.953644990921021
WASP96hr_dropseq_PI24CIRC2_GCGTAAGTGGCC.2	PM3	0.962758004665375
WASP96hr_dropseq_PI24CIRC2_TCGCGTGACCTG.2	PM3	0.963208019733429
WASP96hr_dropseq_PI24CIRC2_GCATCAATCGCT.2	PM3	0.971346020698547
WASP96hr_dropseq_PI24CIRC2_AAGCATTCATAT.2	PM3	0.977301001548767
WASP96hr_dropseq_PI24CIRC2_ACTTGACTGAGA.2	PM3	0.977818012237549
WASP96hr_dropseq_PI24CIRC2_TTTCCATATATC.2	PM3	0.977922022342682
WASP96hr_dropseq_PI24CIRC2_AAGGTGGGATCG.2	PM3	0.981245994567871
WASP96hr_dropseq_PI24CIRC2_GATAGTTACAGA.2	PM3	0.985225021839142
WASP96hr_dropseq_PI24CIRC2_TGAAGACTTTCC.2	PM3	0.98617297410965
WASP96hr_dropseq_PI24CIRC2_CAGCAGCCCGAG.2	PM3	0.993183016777039
WASP96hr_dropseq_PI24CIRC2_CCATAGTTTTAT.2	PM3	0.997915983200073
WASP96hr_dropseq_PI24CIRC2_CTATTCATGACC.2	PM3	1.00117003917694
WASP96hr_dropseq_PI24CIRC2_ATTAGAATAGCA.2	PM3	1.00127995014191
WASP96hr_dropseq_PI24CIRC2_TAATCTGACATC.2	PM3	1.00181996822357
WASP96hr_dropseq_PI24CIRC2_GATGTTTTATCG.2	PM3	1.00636994838715
WASP96hr_dropseq_PI24CIRC2_GAGCTTGTTTCT.2	PM3	1.01129996776581
WASP96hr_dropseq_PI24CIRC2_AGGTCCGAACCT.2	PM3	1.01678001880646
WASP96hr_dropseq_PI24CIRC2_AAGCCAGCAGGA.2	PM3	1.02634000778198
WASP96hr_dropseq_PI24CIRC2_GATCAGCCAAAG.2	PM3	1.0278400182724
WASP96hr_dropseq_PI24CIRC2_AGATGATGTTCG.2	PM3	1.02847003936768
WASP96hr_dropseq_PI24CIRC2_ACGCACACTGGT.2	PM3	1.02980005741119
WASP96hr_dropseq_PI24CIRC2_GCCTGAGCTCGA.2	PM3	1.03154003620148
WASP96hr_dropseq_PI24CIRC3_GTGAAAGCCACT.3	PM3	1.03504002094269
WASP96hr_dropseq_PI24CIRC2_GCTTAGATATGA.2	PM3	1.03773999214172
WASP96hr_dropseq_PI24CIRC2_CGGGAATGGGTA.2	PM3	1.04151999950409
UNinjured_tenx_B1_ATCCACCAGATGTTAG	PM3	1.04653000831604
WASP96hr_dropseq_PI24CIRC2_CCCAAACCCCAG.2	PM3	1.05111002922058
WASP96hr_dropseq_PI24CIRC2_CCGTCGGGTGAT.2	PM3	1.05147004127502
WASP96hr_dropseq_PI24CIRC2_GCGTGTTTGTTT.2	PM3	1.0521399974823
WASP96hr_dropseq_PI24CIRC2_GCTTGGTGTATC.2	PM3	1.05390000343323
WASP96hr_dropseq_PI24CIRC2_CAGGGAAGCTTG.2	PM3	1.05408000946045
injured_tenx_B2_TTGGCAACAGTCGATT	PM3	1.05524003505707
WASP96hr_dropseq_PI24CIRC2_TTCTGACAGATC.2	PM3	1.05755996704102
WASP96hr_dropseq_PI24CIRC2_ATTAATCGTGAC.2	PM3	1.06023001670837
WASP96hr_dropseq_PI24CIRC2_CGTAATTGGGAT.2	PM3	1.06076002120972

Cell Barcode	Cluster Name	FBgn0046687
injured_tenx_B2_GATCGTACATAGGATA	PM3	1.06175005435944
WASP96hr_dropseq_PI24CIRC2_CATATTATTTGC.2	PM3	1.06547999382019
WASP96hr_dropseq_PI24CIRC2_CTATTTCTGGAT.2	PM3	1.06673002243042
WASP96hr_dropseq_PI24CIRC3_GTTATTCGCGTA.3	PM3	1.07035994529724
WASP96hr_dropseq_PI24CIRC2_AGCGGGCCATGT.2	PM3	1.08080005645752
WASP96hr_dropseq_PI24CIRC2_CTGTATTCGGGC.2	PM3	1.09808003902435
WASP96hr_dropseq_PI24CIRC2_TGTAAGCCGGTC.2	PM3	1.09927999973297
WASP96hr_dropseq_PI24CIRC2_CGGTTTGATATA.2	PM3	1.10035002231598
WASP96hr_dropseq_PI24CIRC2_TGCTCTCCGACA.2	PM3	1.10209000110626
WASP96hr_dropseq_PI24CIRC2_ACAATTGTCTAG.2	PM3	1.10383999347687
WASP96hr_dropseq_PI24CIRC2_CATGAGTCAGGT.2	PM3	1.10912001132965
WASP96hr_dropseq_PI24CIRC2_TTTGGACTCGAG.2	PM3	1.11068999767303
WASP96hr_dropseq_PI24CIRC2_GGGCAATATGTC.2	PM3	1.11846995353699
injured_indrop_PC1_blood2_CTTAATAG.GGGTCTAG.AGATGGCT	PM3	1.12727999687195
UNinjured_indrop_UC1_blood2_TAAGGCTC_CTAGCACG_ATCCGCTA	PM3	1.13095998764038
WASP96hr_dropseq_PI24CIRC2_TACCTGCTTACG.2	PM3	1.13595998287201
injured_tenx_B2_CCTTCGAGTTACCAGT	PM3	1.14216995239258
WASP96hr_dropseq_PI24CIRC2_TCCCCGGATCCG.2	PM3	1.14581000804901
WASP96hr_dropseq_PI24CIRC2_GCGATTGTAGCT.2	PM3	1.14734995365143
WASP96hr_dropseq_PI24CIRC2_TCCCAGTTGTCC.2	PM3	1.15269005298615
WASP96hr_dropseq_PI24CIRC2_GTAGCACCTTCG.2	PM3	1.15467000007629
WASP96hr_dropseq_PI24CIRC2_GTGTACTGTAAG.2	PM3	1.15626001358032
WASP96hr_dropseq_PI24CIRC2_CGCCCGGGTGCT.2	PM3	1.15817999839783
WASP96hr_dropseq_PI24CIRC2_CCCGTACTGAAC.2	PM3	1.15869998931885
injured_tenx_B2_TCGGGACGTTCCGGCA	PM3	1.16366994380951
injured_indrop_PC1_blood2_CTTAATAG.GCATCGAG.ATACTCTT	PM3	1.16489005088806
injured_indrop_PC1_blood2_CTTAATAG.AGTCCGTG.GATGTGAG	PM3	1.16807997226715
injured_tenx_B2_AGAGTGGCAATCTACG	PM3	1.17577004432678
WASP96hr_dropseq_PI24CIRC2_CATAAGCTACGG.2	PM3	1.17981994152069
injured_tenx_B4_CTAATGGCAGACGCCT	PM3	1.18325996398926
WASP96hr_dropseq_PI24CIRC2_AGGATCAGTTAG.2	PM3	1.18561995029449
injured_indrop_PC1_blood2_CTTAATAG.AAGAAGGT.CATCGCAG	PM3	1.18942999839783
WASP96hr_dropseq_PI24CIRC2_GTCGGCCCATTA.2	PM3	1.19404995441437
WASP96hr_dropseq_PI24CIRC2_GATAGATTGAGG.2	PM3	1.19597995281219
WASP96hr_dropseq_PI24CIRC2_GGATAAGAACTT.2	PM3	1.1964099407196
WASP96hr_dropseq_PI24CIRC2_GCTAGATTCAGA.2	PM3	1.19791996479034
injured_tenx_B4_TTGGCAAGTAGCGTAG	PM3	1.20067000389099
injured_tenx_B4_GGGACCTTCACAGTAC	PM3	1.20246005058289
WASP96hr_dropseq_PI24CIRC2_ACCCTGCCGAAA.2	PM3	1.20310997962952
WASP96hr_dropseq_PI24CIRC2_AGCCGGCGTACC.2	PM3	1.20343995094299
WASP96hr_dropseq_PI24CIRC2_CATACTCTTTAT.2	PM3	1.21381998062134
WASP96hr_dropseq_PI24CIRC2_TGTGATCATTCC.2	PM3	1.21530997753143
WASP96hr_dropseq_PI24CIRC2_GGCTTTACCTCG.2	PM3	1.21894001960754
WASP96hr_dropseq_PI24CIRC2_GAGAGAGCCTTA.2	PM3	1.22491002082825
WASP96hr_dropseq_PI24CIRC2_AAGTTCCACACA.2	PM3	1.23345994949341
WASP96hr_dropseq_PI24CIRC2_CTAGGTTGGATA.2	PM3	1.23476004600525

Cell Barcode	Cluster Name	FBgn0046687
WASP96hr_dropseq_PI24CIRC2_CGATGCACGCAG.2	PM3	1.23571002483368
UNinjured_tenx_B3_CCTCAGTCAGACGCCT	PM3	1.23987996578217
WASP96hr_dropseq_PI24CIRC2_CAACGAGTCCTA.2	PM3	1.24074995517731
injured_indrop_PC1_blood2_CTTAATAG.CCAGCGAA.CGAGATGT	PM3	1.24636995792389
WASP96hr_dropseq_PI24CIRC2_TTACGGCGCCAG.2	PM3	1.24914002418518
WASP96hr_dropseq_PI24CIRC2_AACAAGAATCGC.2	PM3	1.25009000301361
WASP96hr_dropseq_PI24CIRC2_CAGCAGAGTTGA.2	PM3	1.25080001354218
injured_indrop_PC1_blood2_CTTAATAG.TGAGAGCG.ACATCTAT	PM3	1.25418996810913
injured_tenx_B2_GGGCATCCAAAGGTGC	PM3	1.25508999824524
WASP96hr_dropseq_PI24CIRC2_GAGTATTGCCGT.2	PM3	1.25680005550385
UNinjured_tenx_B1_AACTCTTTCTACTATC	PM3	1.25724995136261
WASP96hr_dropseq_PI24CIRC2_CGAATTAACGAG.2	PM3	1.25923001766205
WASP96hr_dropseq_PI24CIRC2_GCTATCGCATCT.2	PM3	1.25923001766205
WASP96hr_dropseq_PI24CIRC2_TTGGTTAGGCCC.2	PM3	1.26130998134613
WASP96hr_dropseq_PI24CIRC2_AGAGGCTAGGAA.2	PM3	1.26421999931335
WASP96hr_dropseq_PI24CIRC2_CACTTTACGTAT.2	PM3	1.27601003646851
WASP96hr_dropseq_PI24CIRC2_TGGGACAATACA.2	PM3	1.28031003475189
injured_indrop_PC1_blood2_CTTAATAG.CGTGGGAT.CTTCAGGT	PM3	1.28095996379852
WASP96hr_dropseq_PI24CIRC2_AACCGACCGCAG.2	PM3	1.28891003131866
WASP96hr_dropseq_PI24CIRC2_CCACGTATCACC.2	PM3	1.29683005809784
WASP96hr_dropseq_PI24CIRC2_CGTGGAGTCAGT.2	PM3	1.29928004741669
WASP96hr_dropseq_PI24CIRC2_CATACAGCCTAG.2	PM3	1.30435001850128
injured_indrop_PC1_blood2_CTTAATAG.AGAAACCA.GCTCTAGT	PM3	1.30965995788574
WASP96hr_dropseq_PI24CIRC2_GGCACTCTGTTC.2	PM3	1.31113994121552
WASP96hr_dropseq_PI24CIRC2_AGTAAGATTAGC.2	PM3	1.3120299577713
WASP96hr_dropseq_PI24CIRC2_GTAGACGGTGAT.2	PM3	1.31421995162964
WASP96hr_dropseq_PI24CIRC2_GTTCTTGTCTGA.2	PM3	1.31921005249023
WASP96hr_dropseq_PI24CIRC2_CAGCAAGACATG.2	PM3	1.32375001907349
WASP96hr_dropseq_PI24CIRC2_GTATGAACGCCC.2	PM3	1.33653998374939
WASP96hr_dropseq_PI24CIRC2_CAGTAGGCGGTT.2	PM3	1.3377799987793
injured_tenx_B2_GAAATGACAGGACCCT	PM3	1.34591996669769
WASP96hr_dropseq_PI24CIRC2_TCTGTCAAATCG.2	PM3	1.34887003898621
WASP96hr_dropseq_PI24CIRC2_GACGATTAGCAC.2	PM3	1.35013997554779
WASP96hr_dropseq_PI24CIRC2_TCGATGAGTAAG.2	PM3	1.3515499830246
WASP96hr_dropseq_PI24CIRC2_CTCACCTAATCA.2	PM3	1.3578599691391
WASP96hr_dropseq_PI24CIRC2_GACTAGTCGAGG.2	PM3	1.36601996421814
injured_tenx_B2_CATTCGCGTTCCCGAG	PM3	1.36623001098633
WASP96hr_dropseq_PI24CIRC2_GCTGCATCTTAC.2	PM3	1.36775994300842
WASP96hr_dropseq_PI24CIRC2_ATGGCCTGCGCC.2	PM3	1.37147998809814
WASP96hr_dropseq_PI24CIRC2_CAGAACTAGTAA.2	PM3	1.37511003017426
WASP96hr_dropseq_PI24CIRC2_ACGCACGATCGA.2	PM3	1.37688004970551
WASP96hr_dropseq_PI24CIRC2_CAGAAACCAATG.2	PM3	1.38997995853424
WASP96hr_dropseq_PI24CIRC2_CCTACTTATAAC.2	PM3	1.39134001731873
UNinjured_indrop_UC1_blood2_TAAGGCTC_GGTAGCCA_CAAGTCAT	PM3	1.3931599855423
injured_indrop_PC1_blood2_CTTAATAG.CTGTTGTG.GTTTACGT	PM3	1.39339005947113
injured_indrop_PC1_blood2_CTTAATAG.TCAGCCTC.AACCTGAC	PM3	1.39590001106262

Cell Barcode	Cluster Name	FBgn0046687
WASP96hr_dropseq_PI24CIRC2_GCTCTAGAAGAA.2	PM3	1.40094995498657
injured_tenx_B4_CGCCAAGCAGCGATCC	PM3	1.40627002716064
WASP96hr_dropseq_PI24CIRC2_ACTCATGATCAN.2	PM3	1.4118800163269
injured_tenx_B4_ACATACGGTCCAACTA	PM3	1.41258001327515
WASP96hr_dropseq_PI24CIRC2_GTGACGCGGCCT.2	PM3	1.41381001472473
injured_indrop_PC1_blood2_CTTAATAG.ATCTTGGC.TGATGCCC	PM3	1.4321700334549
injured_tenx_B4_AGCAGCCTCGGATGTT	PM3	1.43630003929138
injured_tenx_B2_CTAACTTCAAACCCAT	PM3	1.43801999092102
WASP96hr_dropseq_PI24CIRC2_ACGAGTCTTTCC.2	PM3	1.44516003131866
WASP96hr_dropseq_PI24CIRC2_CCTATACTGCGA.2	PM3	1.44888997077942
UNinjured_tenx_B1_TCAATCTTCACTTCAT	PM3	1.45516002178192
injured_indrop_PC1_blood2_CTTAATAG.AACGATTT.CACTCCTC	PM3	1.4586900472641
injured_tenx_B4_TGAGCATGTGGACGAT	PM3	1.4592000246048
injured_indrop_PC1_blood2_CTTAATAG.ATGCATAT.CAACGTGG	PM3	1.46174001693726
WASP96hr_dropseq_PI24CIRC3_CGACGGTTTTAC.3	PM3	1.46378004550934
WASP96hr_dropseq_PI24CIRC2_CGAACTGAGGCG.2	PM3	1.46505999565125
WASP96hr_dropseq_PI24CIRC2_TTTTTCTGTAGA.2	PM3	1.46505999565125
WASP96hr_dropseq_PI24CIRC3_TACAGGTTTTCG.3	PM3	1.4864000082016
WASP96hr_dropseq_PI24CIRC2_CGGTTTCTATTA.2	PM3	1.48834002017975
injured_tenx_B2_CGATTGAAGAGTCTGG	PM3	1.49251997470856
WASP96hr_dropseq_PI24CIRC2_TGACATGGCATG.2	PM3	1.49251997470856
injured_indrop_PC2_blood3_TCGCATAA.TCGCAATC.CAAGTCAT	PM3	1.4930499792099
UNinjured_indrop_UC1_blood2_TAAGGCTC_GGTCTTTC_CGGCACAT	PM3	1.49439001083374
injured_indrop_PC1_blood2_CTTAATAG.GGCGACAA.CTCCGCAT	PM3	1.49707996845245
WASP96hr_dropseq_PI24CIRC2_CTACCCGGCAAG.2	PM3	1.49869000911713
injured_tenx_B2_CAAGTTGTCACCAGGC	PM3	1.50031995773315
UNinjured_indrop_UC1_blood2_TAAGGCTC_TAAGGGCC_TGATGCCC	PM3	1.50302994251251
injured_tenx_B4_GCGCAACCACGACTCG	PM3	1.51426994800568
injured_indrop_PC1_blood2_CTTAATAG.GAGGAGTG.ACGCAGAG	PM3	1.51648998260498
injured_tenx_B2_TTTACTGCAATAACGA	PM3	1.52318000793457
UNinjured_indrop_UC1_blood2_TAAGGCTC_CGAAACTC_TCATGAGG	PM3	1.52487003803253
UNinjured_indrop_UC1_blood2_TAAGGCTC_AGAAACCA_AGGAGCTT	PM3	1.53079998493195
WASP96hr_dropseq_PI24CIRC2_AGTTATGTCGGA.2	PM3	1.53550004959106
injured_tenx_B4_GGCGACTAGGCTCAGA	PM3	1.54543995857239
WASP96hr_dropseq_PI24CIRC2_CATAAGCTACGC.2	PM3	1.54981005191803
injured_tenx_B4_CATCGAACACGAAACG	PM3	1.55157005786896
WASP96hr_dropseq_PI24CIRC2_ATATAGTTAATC.2	PM3	1.55561995506287
WASP96hr_dropseq_PI24CIRC3_TGACTGGATCGG.3	PM3	1.55774998664856
WASP96hr_dropseq_PI24CIRC2_ATCCCGATTGAG.2	PM3	1.5648900270462
WASP96hr_dropseq_PI24CIRC2_TAGTTGCTTTAG.2	PM3	1.56999003887177
UNinjured_indrop_UC1_blood2_TAAGGCTC_TGGAAAGC_TCCCAATC	PM3	1.57089996337891
WASP96hr_dropseq_PI24CIRC2_ACTATCCTCCAT.2	PM3	1.57113003730774
UNinjured_indrop_UC1_blood2_TAAGGCTC_GATGTGGC_ACTGCCGT	PM3	1.57422995567322
WASP96hr_dropseq_PI24CIRC2_GGCGGGCCAACN.2	PM3	1.57757997512817
injured_indrop_PC1_blood2_CTTAATAG.ATATGCAA.GGAATAGA	PM3	1.58864998817444
WASP96hr_dropseq_PI24CIRC2_AACCGACCGCAC.2	PM3	1.60054004192352

Cell Barcode	Cluster Name	FBgn0046687
WASP96hr_dropseq_PI24CIRC2_AGATAAGGATGC.2	PM3	1.60212004184723
WASP96hr_dropseq_PI24CIRC2_AGATCAACGAGT.2	PM3	1.610720038414
WASP96hr_dropseq_PI24CIRC2_TCCTTGCTCTAT.2	PM3	1.61586999893188
WASP96hr_dropseq_PI24CIRC2_ACTATTTGACTG.2	PM3	1.61684000492096
WASP96hr_dropseq_PI24CIRC2_GGCGGATCGTAN.2	PM3	1.61846005916595
injured_indrop_PC1_blood2_CTTAATAG.ACGTAAAC.GCAAAGCC	PM3	1.62106001377106
injured_indrop_PC2_blood3_TCGCATAA.TAGTAGCC.TTGCACGC	PM3	1.62595999240875
UNinjured_indrop_UC1_blood2_TAAGGCTC_ATTGGCCC_GGGAACCT	PM3	1.63155996799469
WASP96hr_dropseq_PI24CIRC2_TGCCTAGTACTG.2	PM3	1.63287997245789
UNinjured_indrop_UC1_blood2_TAAGGCTC_CTAACGCC_CAGGTGTC	PM3	1.63287997245789
WASP96hr_dropseq_PI24CIRC2_GACGATTAGCAT.2	PM3	1.64256000518799
WASP96hr_dropseq_PI24CIRC2_CATACAATGAGG.2	PM3	1.64425003528595
WASP96hr_dropseq_PI24CIRC2_CATACATGGTAG.2	PM3	1.65033996105194
injured_indrop_PC1_blood2_CTTAATAG.TAGTAGCC.CACTTTCT	PM3	1.65102005004883
injured_indrop_PC1_blood2_CTTAATAG.ACCCGATT.CTTCGATT	PM3	1.65340995788574
injured_tenx_B2_CTGGTCTAGGCCGAAT	PM3	1.65717995166779
UNinjured_indrop_UC1_blood2_TAAGGCTC_GCGTATTC_CTTCAGGT	PM3	1.65821003913879
injured_tenx_B4_ACTTACTGTTCAACCA	PM3	1.66130995750427
injured_tenx_B4_GATCGATAGACAAAGG	PM3	1.66408002376556
injured_indrop_PC1_blood2_CTTAATAG.CATGACGA.TCCTTATT	PM3	1.66825997829437
injured_indrop_PC1_blood2_CTTAATAG.AGTAGTTA.AAGTAATC	PM3	1.67175996303558
WASP96hr_dropseq_PI24CIRC2_CACGATTATACG.2	PM3	1.67703998088837
injured_indrop_PC1_blood2_CTTAATAG.GCTATGGG.CTTCAGGT	PM3	1.68487000465393
injured_indrop_PC1_blood2_CTTAATAG.CTTCTACG.GGGAGGTA	PM3	1.69097995758057
WASP96hr_dropseq_PI24CIRC2_TTTACGAATTGA.2	PM3	1.70018005371094
WASP96hr_dropseq_PI24CIRC2_CACAACCCGTGC.2	PM3	1.70097005367279
WASP96hr_dropseq_PI24CIRC2_AGCAGCATTACT.2	PM3	1.71185994148254
UNinjured_indrop_UC1_blood2_TAAGGCTC_AGTCACAA_CGAGTCTG	PM3	1.7288099527359
injured_tenx_B2_GAATGAAAGTCTCAAC	PM3	1.73378002643585
WASP96hr_dropseq_PI24CIRC2_GATAGAAGAACT.2	PM3	1.73839998245239
WASP96hr_dropseq_PI24CIRC2_GATAGAAGAACC.2	PM3	1.7457799911499
WASP96hr_dropseq_PI24CIRC2_CCCATCCCGGAC.2	PM3	1.75086998939514
injured_indrop_PC2_blood3_TCGCATAA.GTGATAAA.CTCGATGC	PM3	1.75639998912811
WASP96hr_dropseq_PI24CIRC2_CTGCGCCAATTA.2	PM3	1.7575900554657
WASP96hr_dropseq_PI24CIRC2_CGGCATCGGATA.2	PM3	1.76408004760742
injured_tenx_B2_CACACCTGTTCGTTGA	PM3	1.76637995243073
injured_tenx_B2_ATAAGAGAGTGGAGAA	PM3	1.78059995174408
WASP96hr_dropseq_PI24CIRC2_GCCATTAAGTGC.2	PM3	1.78760004043579
injured_tenx_B2_CGCGTTTTCTATGTGG	PM3	1.81354999542236
WASP96hr_dropseq_PI24CIRC2_TAACCACTGTGT.2	PM3	1.81505000591278
injured_tenx_B2_AGCGTCGCATTAACCG	PM3	1.82023000717163
injured_indrop_PC2_blood3_TCGCATAA.ACCTGAAG.GGGTTGGT	PM3	1.84718000888824
injured_tenx_B4_ACAGCCGCAGGTCCAC	PM3	1.85258996486664
injured_tenx_B4_TGACAACAGTCATGCT	PM3	1.86195003986359
WASP96hr_dropseq_PI24CIRC2_TACTCTCAACAG.2	PM3	1.87890005111694
UNinjured_tenx_B3_AACTCCCAGTCAAGGC	PM3	1.88338005542755

Cell Barcode	Cluster Name	FBgn0046687
WASP96hr_dropseq_PI24CIRC2_AGGGCAAAACTA.2	PM3	1.88384997844696
WASP96hr_dropseq_PI24CIRC2_TGGAGAAGATTT.2	PM3	1.96606004238129
injured_tenx_B2_CTGAAACGTTGCGCAC	PM3	1.96923995018005
UNinjured_tenx_B3_TTCTACACACGACTCG	PM3	1.99185001850128
injured_tenx_B2_GTCTTCGAGAGTCGGT	PM3	2.02823996543884
WASP96hr_dropseq_PI24CIRC2_AAATTTGTTCGG.2	PM3	2.03004002571106
WASP96hr_dropseq_PI24CIRC2_AACTGTCCGTCC.2	PM3	2.03504991531372
WASP96hr_dropseq_PI24CIRC2_CGCGTTGCACCG.2	PM3	2.0378999710083
WASP96hr_dropseq_PI24CIRC2_GCGCTGCCTCGT.2	PM3	2.04790997505188
WASP96hr_dropseq_PI24CIRC2_ATGGATCCCCCN.2	PM3	2.06491994857788
WASP96hr_dropseq_PI24CIRC3_GATTCGCAGGCT.3	PM3	2.07489991188049
WASP96hr_dropseq_PI24CIRC2_TATTATTCAGCG.2	PM3	2.10028004646301
injured_indrop_PC1_blood2_CTTAATAG.AAACACTA.GTCAATAC	PM3	2.14359998703003
injured_indrop_PC1_blood2_CTTAATAG.AAGGATGA.GGTCACAG	PM3	2.14560008049011
injured_tenx_B2_TTGCGTCTCACATGCA	PM3	2.2166600227356
injured_tenx_B2_CTAACTTTCGGCTACG	PM3	2.22888994216919
injured_tenx_B2_CGAGAAGCATTAGGCT	PM3	2.22914004325867
UNinjured_indrop_UC1_blood2_TAAGGCTC_ATAAGCTA_GACAGATA	PM3	2.25240993499756
injured_tenx_B4_ACACCCTGTAAGGATT	PM3	2.3014600276947
injured_tenx_B2_CGGCTAGCACCAGTTA	PM3	2.36929988861084
WASP96hr_dropseq_PI24CIRC2_CATCGCGACTCT.2	PM3	2.80155992507935
WASP96hr_dropseq_PI24CIRC2_AATTACTAGTTA.2	PM4	0.362881004810333
WASP96hr_dropseq_PI24CIRC3_CGGATGCCCCGT.3	PM4	0.536743998527527
WASP96hr_dropseq_PI24CIRC3_GGGCCCACACGA.3	PM4	0.543983995914459
WASP96hr_dropseq_PI24CIRC3_GGATGACAAAAT.3	PM4	0.56920999288559
WASP96hr_dropseq_PI24CIRC3_TCGGTCTAGCGT.3	PM4	0.602596998214722
WASP96hr_dropseq_PI24CIRC3_AACTGGATATTG.3	PM4	0.645245015621185
WASP96hr_dropseq_PI24CIRC3_GCCATTAAACTG.3	PM4	0.728201985359192
WASP96hr_dropseq_PI24CIRC3_CCTCATATCTGC.3	PM4	0.74991500377655
WASP96hr_dropseq_PI24CIRC3_ATATGACGGTTA.3	PM4	0.767111003398895
WASP96hr_dropseq_PI24CIRC3_GCGCCGCCCTTT.3	PM4	0.77208799123764
WASP96hr_dropseq_PI24CIRC2_TCTGGCAACCTT.2	PM4	0.821133017539978
WASP96hr_dropseq_PI24CIRC2_ATGAAGCGTGCA.2	PM4	0.849897027015686
injured_tenx_B4_TGATTTCGTCAATGTC	PM4	0.873013973236084
WASP96hr_dropseq_PI24CIRC3_CATTTTAAAATG.3	PM4	0.880634009838104
WASP96hr_dropseq_PI24CIRC3_GGACCAAACACG.3	PM4	0.898114025592804
WASP96hr_dropseq_PI24CIRC3_ATAGCGTGCAAA.3	PM4	0.920421004295349
WASP96hr_dropseq_PI24CIRC2_GAGACGGCCGAG.2	PM4	0.948575019836426
WASP96hr_dropseq_PI24CIRC3_GCGTCCAACAGA.3	PM4	0.953056991100311
WASP96hr_dropseq_PI24CIRC1_GGTACTACTGGA.1	PM4	1.04568994045258
WASP96hr_dropseq_PI24CIRC3_CACAAACGATGC.3	PM4	1.04928994178772
WASP96hr_dropseq_PI24CIRC3_TTGTACATAAGT.3	PM4	1.05633997917175
WASP96hr_dropseq_PI24CIRC3_TTCATCGTCCGA.3	PM4	1.07035994529724
WASP96hr_dropseq_PI24CIRC3_GAGTTCTTTTCC.3	PM4	1.10062003135681
WASP96hr_dropseq_PI24CIRC2_AGATTGGAAAAG.2	PM4	1.10790002346039
WASP96hr_dropseq_PI24CIRC2_CCTATATGGGTC.2	PM4	1.1275600194931

Cell Barcode	Cluster Name	FBgn0046687
WASP96hr_dropseq_PI24CIRC3_CATGAACGATCT.3	PM4	1.14014005661011
WASP96hr_dropseq_PI24CIRC3_GCTAATCAGTAC.3	PM4	1.14362001419067
UNinjured_tenx_B3_AACGTTGTCTATGTGG	PM4	1.20457994937897
WASP96hr_dropseq_PI24CIRC3_TCGGGGATCTAC.3	PM4	1.20605003833771
injured_tenx_B4_TGAAAGAGTTTAGCTG	PM4	1.21547996997833
WASP96hr_dropseq_PI24CIRC3_ACCTACTACGCA.3	PM4	1.2386599779129
WASP96hr_dropseq_PI24CIRC3_ATGATAGCTTTA.3	PM4	1.24319994449615
WASP96hr_dropseq_PI24CIRC3_CATTCGCTTGTA.3	PM4	1.25045001506805
injured_tenx_B4_GCAGTTAAGATGCCAG	PM4	1.2520500421524
UNinjured_tenx_B3_TCAATCTCACATAACC	PM4	1.26013004779816
injured_tenx_B4_CGTCAGGCAAGGACTG	PM4	1.27553999423981
injured_tenx_B4_GCACATATCAACTCTT	PM4	1.27553999423981
WASP96hr_dropseq_PI24CIRC3_CCTCGAGTAAGC.3	PM4	1.28653001785278
UNinjured_indrop_UC1_blood2_TAAGGCTC_GAGCGGAA_GTTCTGTT	PM4	1.29042994976044
injured_tenx_B4_CTAAGACTCTCCAGGG	PM4	1.29157996177673
WASP96hr_dropseq_PI24CIRC3_ACCAGCCCCGAG.3	PM4	1.29735004901886
injured_tenx_B4_ACGTCAACAGACAAGC	PM4	1.30044996738434
injured_tenx_B4_AAAGCAAGTGGCCCTA	PM4	1.30493998527527
UNinjured_indrop_UC1_blood2_TAAGGCTC_GGTTGAGA_CCTATTCA	PM4	1.31520998477936
WASP96hr_dropseq_PI24CIRC2_GGCCCTTCACAT.2	PM4	1.32304000854492
WASP96hr_dropseq_PI24CIRC3_TCTGCATCCAGA.3	PM4	1.3361200094223
UNinjured_indrop_UC1_blood2_TAAGGCTC_AATGTATG_AGGATTCA	PM4	1.34591996669769
WASP96hr_dropseq_PI24CIRC3_GAAAACAGCTGT.3	PM4	1.34739005565643
WASP96hr_dropseq_PI24CIRC3_TGCCTGGATCAA.3	PM4	1.35225999355316
injured_tenx_B4_GTTTCTAGTCCTGCTT	PM4	1.37258005142212
injured_indrop_PC2_blood3_TCGCATAA.TAACTTCT.TAGTGGAC	PM4	1.37478005886078
injured_tenx_B4_TGCCAAAGTTTACTCT	PM4	1.37565994262695
UNinjured_tenx_B3_CTAAGACTCACAGTAC	PM4	1.38345003128052
injured_tenx_B4_GCTGGGTTCCGCATAA	PM4	1.40094995498657
injured_tenx_B4_TATCAGGTCAGCTGGC	PM4	1.4099999666214
UNinjured_indrop_UC1_blood2_TAAGGCTC_CCTTTGTC_TTCCAGAC	PM4	1.42733001708984
injured_tenx_B4_AACTCCCCATCCCATC	PM4	1.43070995807648
injured_indrop_PC2_blood3_TCGCATAA.AGGGTAAA.GTACACTC	PM4	1.44863998889923
UNinjured_indrop_UC1_blood2_TAAGGCTC_ATCCTAGT_GATCGTTT	PM4	1.45314002037048
WASP96hr_dropseq_PI24CIRC3_GTGTCTACAACA.3	PM4	1.46429002285004
injured_indrop_PC1_blood2_CTTAATAG.AGCACGTA.GTTCAACT	PM4	1.48296999931335
UNinjured_indrop_UC1_blood2_TAAGGCTC_ACGGCAGT_TCGACGGT	PM4	1.49038004875183
WASP96hr_dropseq_PI24CIRC3_TGCACGCTCGGC.3	PM4	1.50085997581482
injured_tenx_B4_GACGGCTGTAGCGTAG	PM4	1.50656998157501
UNinjured_indrop_UC1_blood2_TAAGGCTC_GGTCCTAA_AGTTTAGA	PM4	1.51538002490997
WASP96hr_dropseq_PI24CIRC2_CTGTGTTGAGGA.2	PM4	1.51954996585846
WASP96hr_dropseq_PI24CIRC3_TCAGGCGGGCTA.3	PM4	1.5302300453186
WASP96hr_dropseq_PI24CIRC3_ATGACGAAATTC.3	PM4	1.53038001060486
UNinjured_indrop_UC1_blood2_TAAGGCTC_CCATATGA_TCCCTGAT	PM4	1.54197001457214
injured_tenx_B4_GTTAAGCGTATTCTCT	PM4	1.54659998416901
injured_tenx_B4_CGATGTATCAACGCTA	PM4	1.55244994163513

Cell Barcode	Cluster Name	FBgn0046687
UNinjured_indrop_UC1_blood2_TAAGGCTC_GTAAGCCG_AAACAGGG	PM4	1.56789004802704
injured_tenx_B4_CATGACACAGGCAGTA	PM4	1.57301998138428
WASP96hr_dropseq_PI24CIRC2_GTGTGTTAGCCC.2	PM4	1.58155000209808
injured_indrop_PC2_blood3_TCGCATAA.TGACGTCG.TCACCGAG	PM4	1.60433995723724
injured_indrop_PC2_blood3_TCGCATAA.TATCTGTC.TGAAATGA	PM4	1.61652004718781
WASP96hr_dropseq_PI24CIRC3_CTCGCAAGACGN.3	PM4	1.61715996265411
WASP96hr_dropseq_PI24CIRC3_CACGCAAGATCG.3	PM4	1.62007999420166
injured_tenx_B4_TAGACCAGTTCAGTAC	PM4	1.63388001918793
UNinjured_indrop_UC1_blood2_TAAGGCTC_TATCTGTC_CGGAATTT	PM4	1.6358699798584
injured_tenx_B4_TCTGAGAGTTATGTGC	PM4	1.64055001735687
WASP96hr_dropseq_PI24CIRC2_TGTGGTCAGGCG.2	PM4	1.64268004894257
injured_tenx_B4_CACAGTACAGATCCAT	PM4	1.67175996303558
UNinjured_indrop_UC1_blood2_TAAGGCTC_CTGCGATG_CGTATCGG	PM4	1.68414998054504
injured_tenx_B4_TTTGTCACAATCAGAA	PM4	1.7063000202179
UNinjured_indrop_UC1_blood2_TAAGGCTC_ATCCCACC_TCGACACC	PM4	1.71972000598907
injured_tenx_B4_GTTCATTTCGGTCCGA	PM4	1.72197997570038
injured_indrop_PC1_blood2_CTTAATAG.TATCTGTC.CGACGTCA	PM4	1.74422001838684
injured_indrop_PC2_blood3_TCGCATAA.ACGTAAAC.TAGTGGAC	PM4	1.7532399892807
injured_tenx_B2_CTGAAGTCACACCGAC	PM4	1.76277005672455
injured_tenx_B4_GGACGTCGTGCCTGCA	PM4	1.78968000411987
injured_indrop_PC2_blood3_TCGCATAA.AGTCCGTG.CACAGTTT	PM4	1.79929995536804
injured_indrop_PC2_blood3_TCGCATAA.AGCTCCAC.TACCAGGC	PM4	1.81763994693756
WASP96hr_dropseq_PI24CIRC3_CACATTAAGCTC.3	PM4	1.81957995891571
WASP96hr_dropseq_PI24CIRC2_TATTTTAACGGG.2	PM4	1.83527004718781
WASP96hr_dropseq_PI24CIRC2_GCTAGCGCTCTA.2	PM4	1.84493005275726
UNinjured_tenx_B3_CACTCCACACGTTGGC	PM4	1.85531997680664
WASP96hr_dropseq_PI24CIRC3_TTTTATACTTGA.3	PM4	2.13369011878967
injured_tenx_B2_ACACCGGCATGATCCA	PM4	2.13763999938965
injured_tenx_B4_ATTACTCGTAGGCATG	PM4	2.16210007667542
WASP96hr_dropseq_PI24CIRC3_GGTGCGGCGGAG.3	PM4	2.30064988136292
injured_tenx_B4_CCTTACGCAAGTAATG	PM4	2.32818007469177
WASP96hr_dropseq_PI24CIRC3_ATGAACTGCGCT.3	PM4	2.96088004112244
UNinjured_tenx_B3_CAGCTGGAGTGATCGG	PM5	0.45541900396347
WASP96hr_dropseq_PI24CIRC1_GCTAGCCAACCC.1	PM5	0.462951004505157
WASP96hr_dropseq_PI24CIRC2_GATTTGGTGTGT.2	PM5	0.482712000608444
WASP96hr_dropseq_PI24CIRC3_TTCCCTCAAATC.3	PM5	0.49905601143837
WASP96hr_dropseq_PI24CIRC2_AAGTTCGAGTAA.2	PM5	0.504377007484436
WASP96hr_dropseq_PI24CIRC2_CGGGGGTCCCTA.2	PM5	0.599026024341583
WASP96hr_dropseq_PI24CIRC2_GCTGGCGAGCCA.2	PM5	0.615917026996613
WASP96hr_dropseq_PI24CIRC2_CGCGGGCAATCG.2	PM5	0.651293992996216
WASP96hr_dropseq_PI24CIRC2_CTTTGTGATGCA.2	PM5	0.663385987281799
WASP96hr_dropseq_PI24CIRC3_TATCAGCAGGCT.3	PM5	0.680876970291138
WASP96hr_dropseq_PI24CIRC3_CCGGCTACATTA.3	PM5	0.685584008693695
WASP96hr_dropseq_PI24CIRC3_GTTTGGTAGCGG.3	PM5	0.68833202123642
WASP96hr_dropseq_PI24CIRC3_ATGTTCCTTTTC.3	PM5	0.690457999706268
WASP96hr_dropseq_PI24CIRC2_ATTCATCTAAAA.2	PM5	0.730871975421906

Cell Barcode	Cluster Name	FBgn0046687
WASP96hr_dropseq_PI24CIRC2_AATTTCTTTGAG.2	PM5	0.737664997577667
WASP96hr_dropseq_PI24CIRC2_TCGCCGGGCCAG.2	PM5	0.758803009986877
WASP96hr_dropseq_PI24CIRC3_TCCTCCCCGGCT.3	PM5	0.759891986846924
WASP96hr_dropseq_PI24CIRC3_ACGTAGACATGC.3	PM5	0.784506976604462
injured_tenx_B2_GAACGGAGTGCGCTTG	PM5	0.78802102804184
WASP96hr_dropseq_PI24CIRC3_CCATGAAAGCTC.3	PM5	0.805962979793549
WASP96hr_dropseq_PI24CIRC3_ACACGCTCATCT.3	PM5	0.80747401714325
WASP96hr_dropseq_PI24CIRC3_GACCTAGACACG.3	PM5	0.812810003757477
WASP96hr_dropseq_PI24CIRC2_ACCTTGGTTTCC.2	PM5	0.837440013885498
WASP96hr_dropseq_PI24CIRC3_GCTGACGGCTTG.3	PM5	0.83900398015976
WASP96hr_dropseq_PI24CIRC3_GGAGTCTCAGCC.3	PM5	0.839824974536896
WASP96hr_dropseq_PI24CIRC2_CGCTCGAGCCTA.2	PM5	0.867170989513397
UNinjured_tenx_B3_CTAATGGTCAGCATGT	PM5	0.894336998462677
WASP96hr_dropseq_PI24CIRC3_TCATGCCAGCCA.3	PM5	0.90371298789978
WASP96hr_dropseq_PI24CIRC3_CTTATTACCTTA.3	PM5	0.916800975799561
WASP96hr_dropseq_PI24CIRC3_GCGTCGCTCGGT.3	PM5	0.918516993522644
WASP96hr_dropseq_PI24CIRC3_TCATGAGCGCCG.3	PM5	0.933145999908447
WASP96hr_dropseq_PI24CIRC2_CCAATCGTCGAC.2	PM5	0.934082984924316
UNinjured_indrop_UC1_blood2_TAAGGCTC_CTAACGCC_CTTAGGTA	PM5	0.963158011436462
WASP96hr_dropseq_PI24CIRC2_CACGATGTTGCG.2	PM5	0.96806001663208
WASP96hr_dropseq_PI24CIRC3_TTGGCTCTAACG.3	PM5	0.981455028057098
WASP96hr_dropseq_PI24CIRC3_GGATAAAGTGCG.3	PM5	1.00785994529724
UNinjured_indrop_UC1_blood2_TAAGGCTC_GTGAGGCA_GTCTCTCT	PM5	1.0166699886322
WASP96hr_dropseq_PI24CIRC3_CTACTCTTCGAA.3	PM5	1.0289900302887
WASP96hr_dropseq_PI24CIRC3_GACTTGCGCCCC.3	PM5	1.04022002220154
UNinjured_tenx_B3_CACACTCTCGGAGGTA	PM5	1.04246997833252
WASP96hr_dropseq_PI24CIRC2_GGCAGGTACGCC.2	PM5	1.05061995983124
WASP96hr_dropseq_PI24CIRC3_TCGTTTATTGAG.3	PM5	1.05086004734039
WASP96hr_dropseq_PI24CIRC3_TCACGGACCGCA.3	PM5	1.05413997173309
injured_tenx_B4_GCACATAGTTGTCTTT	PM5	1.05664002895355
WASP96hr_dropseq_PI24CIRC2_GACACGGATATA.2	PM5	1.06834995746613
WASP96hr_dropseq_PI24CIRC3_ATACATTTAGAG.3	PM5	1.07824003696442
WASP96hr_dropseq_PI24CIRC3_AAGTGATCACTG.3	PM5	1.0791300535202
injured_tenx_B4_CTACGTCTCGGATGGA	PM5	1.08415997028351
WASP96hr_dropseq_PI24CIRC2_TACATCGTTGAG.2	PM5	1.08687996864319
WASP96hr_dropseq_PI24CIRC2_CAACTCAAGCCC.2	PM5	1.09475994110107
WASP96hr_dropseq_PI24CIRC2_ACATCTAGCCAG.2	PM5	1.09652996063232
injured_tenx_B4_GCTGGGTAGCGATGAC	PM5	1.11583995819092
WASP96hr_dropseq_PI24CIRC3_CCTTCAAGTGAC.3	PM5	1.11998999118805
WASP96hr_dropseq_PI24CIRC2_TACATCGTTGAC.2	PM5	1.13338005542755
WASP96hr_dropseq_PI24CIRC3_TTGTAGTTTATT.3	PM5	1.13381004333496
injured_indrop_PC2_blood3_TCGCATAA.CACTAACC.ACGCTCAG	PM5	1.13480997085571
UNinjured_indrop_UC1_blood2_TAAGGCTC_AAGAAGGT_AAAGTCGG	PM5	1.13582003116608
injured_tenx_B4_AAACGGGTCCTTCAAT	PM5	1.16140997409821
WASP96hr_dropseq_PI24CIRC3_TGTCTGTCGCCT.3	PM5	1.17809998989105
injured_tenx_B2_GCCTCTAAGGTCGGAT	PM5	1.18515002727509

Cell Barcode	Cluster Name	FBgn0046687
injured_tenx_B4_TCGGGACTCACAATGC	PM5	1.19372999668121
WASP96hr_dropseq_PI24CIRC3_TTACATCTCATC.3	PM5	1.20381999015808
WASP96hr_dropseq_PI24CIRC2_CACGTTACATCT.2	PM5	1.22220003604889
injured_indrop_PC2_blood3_TCGCATAA.TATCCACG.TGTAGTTT	PM5	1.22525000572205
UNinjured_indrop_UC1_blood2_TAAGGCTC_GCTAAGTT_ATCCGCTA	PM5	1.22780001163483
injured_indrop_PC1_blood2_CTTAATAG.CTTCTACG.CGAACGTA	PM5	1.23190999031067
WASP96hr_dropseq_PI24CIRC3_GGGAATTGTGTC.3	PM5	1.2364000082016
UNinjured_indrop_UC1_blood2_TAAGGCTC_TGGACGGA_AACCTGAC	PM5	1.23830997943878
WASP96hr_dropseq_PI24CIRC3_CCCACTGGGGTC.3	PM5	1.25329995155334
WASP96hr_dropseq_PI24CIRC3_GAGTCAATTTTG.3	PM5	1.26449000835419
WASP96hr_dropseq_PI24CIRC3_GTGTGACGTCGT.3	PM5	1.28001999855042
WASP96hr_dropseq_PI24CIRC3_CTATCTTTCTAA.3	PM5	1.28454005718231
WASP96hr_dropseq_PI24CIRC3_TGTTACAATTCT.3	PM5	1.30357003211975
injured_indrop_PC2_blood3_TCGCATAA.GACTAGCG.TCTGTGGT	PM5	1.30357003211975
WASP96hr_dropseq_PI24CIRC3_GCGTGTTTCCGC.3	PM5	1.32243001461029
WASP96hr_dropseq_PI24CIRC2_AGTCGTCATGAC.2	PM5	1.32617998123169
injured_indrop_PC1_blood2_CTTAATAG.GGGCATCA.TTCCAGAC	PM5	1.32913994789124
injured_tenx_B4_CTCCTAGCAACGATGG	PM5	1.33965003490448
injured_tenx_B4_ACACCCTTCCCATTAT	PM5	1.36907005310059
WASP96hr_dropseq_PI24CIRC2_CCGAAGACTCAC.2	PM5	1.37589001655579
UNinjured_indrop_UC1_blood2_TAAGGCTC_CACTTGAT_ACCACGCT	PM5	1.39089000225067
injured_tenx_B2_CATCAAGGTTGTACAC	PM5	1.3970400094986
WASP96hr_dropseq_PI24CIRC3_ACTCTAAGCCGA.3	PM5	1.40922999382019
injured_tenx_B4_TGAGCATTCACCTTAT	PM5	1.41729998588562
UNinjured_indrop_UC1_blood2_TAAGGCTC_ACCCTCAA_GTTCAACT	PM5	1.42157995700836
WASP96hr_dropseq_PI24CIRC2_AAAGTCCGTCTA.2	PM5	1.42396998405457
WASP96hr_dropseq_PI24CIRC3_GAGTCTAGAGGT.3	PM5	1.42420995235443
WASP96hr_dropseq_PI24CIRC2_CCCTGCCCATAA.2	PM5	1.44354999065399
WASP96hr_dropseq_PI24CIRC3_GCGTGTTTCCGG.3	PM5	1.44516003131866
injured_indrop_PC2_blood3_TCGCATAA.GATGCAGA.AACCCTTG	PM5	1.46890997886658
injured_indrop_PC2_blood3_TCGCATAA.AAGAGTAT.TCCTTATT	PM5	1.47045004367828
WASP96hr_dropseq_PI24CIRC3_AAACTGACCCCG.3	PM5	1.47329998016357
injured_indrop_PC1_blood2_CTTAATAG.GCCTGGTA.GTCATATT	PM5	1.48719000816345
WASP96hr_dropseq_PI24CIRC2_CTTAGACAGGGG.2	PM5	1.51133000850677
injured_indrop_PC1_blood2_CTTAATAG.GTGAACTC.GCGCATTC	PM5	1.51481997966766
injured_indrop_PC1_blood2_CTTAATAG.CAAGGGTT.TCGACGGT	PM5	1.52066004276276
injured_indrop_PC1_blood2_CTTAATAG.GACACAAA.CGTACCTA	PM5	1.521360039711
WASP96hr_dropseq_PI24CIRC2_TCCTCCTCGTCG.2	PM5	1.56508994102478
injured_tenx_B4_TTCGAAGCAACAACCT	PM5	1.56728994846344
UNinjured_tenx_B3_CTTAGGACATGCCACG	PM5	1.57211005687714
injured_indrop_PC1_blood2_CTTAATAG.CTCCCAAA.ACGCAGAG	PM5	1.59801995754242
WASP96hr_dropseq_PI24CIRC3_TGCTGCGTAGAA.3	PM5	1.61942994594574
injured_tenx_B4_ACATCAGCATAAGACA	PM5	1.63554000854492
injured_tenx_B2_TCGTACCTCTCGATGA	PM5	1.64898002147675
WASP96hr_dropseq_PI24CIRC3_GGGTGACTCGCC.3	PM5	1.66304004192352
UNinjured_tenx_B3_TGGTTAGTCGGATGGA	PM5	1.66478002071381

Cell Barcode	Cluster Name	FBgn0046687
UNinjured_indrop_UC1_blood2_TAAGGCTC_GCTAAGTT_ACAGCGGA	PM5	1.67917001247406
UNinjured_tenx_B3_TAGGCATAGTCATGCT	PM5	1.68379998207092
injured_tenx_B2_CGTTAGATCAGAGGTG	PM5	1.70594000816345
UNinjured_indrop_UC1_blood2_TAAGGCTC_CCGAGATC_GGATAAAG	PM5	1.75720000267029
injured_indrop_PC2_blood3_TCGCATAA.CTTCTACG.TGGCCTGT	PM5	1.75958001613617
UNinjured_indrop_UC1_blood2_TAAGGCTC_ACAAGTAG_ACAGCGGA	PM5	1.80140995979309
injured_tenx_B4_ACTTACTTCGCCAGCA	PM5	1.81850004196167
WASP96hr_dropseq_PI24CIRC3_GGCCACGGTTAG.3	PM5	1.90100002288818
injured_indrop_PC2_blood3_TCGCATAA.TAGCCACA.GTCTCTCT	PM5	1.90278005599976
injured_tenx_B2_AAGGCAGAGTACATGA	PM5	1.9145200252533
UNinjured_indrop_UC1_blood2_TAAGGCTC_AACTGCCT_AAAGTCGG	PM5	1.94214999675751
WASP96hr_dropseq_PI24CIRC3_CAGCCCGTCCCA.3	PM5	2.02081990242004
injured_tenx_B2_AAGGCAGCAGATGGGT	PM5	2.05982995033264
injured_tenx_B2_AAGTCTGAGAATTCCC	PM5	2.05982995033264
injured_tenx_B2_AAGACCTTCCCTCTTT	PM5	2.180340051651
WASP96hr_dropseq_PI24CIRC3_GAGTGGCGTGTG.3	PM5	2.40336990356445
injured_tenx_B2_TGCGGGTGTCTCTCGT	PM5	2.64783000946045
injured_tenx_B4_GACTGCGAGAAGGTTT	CC2	0.383298009634018
injured_tenx_B4_TAGAGCTAGAAACCAT	CC2	0.45184001326561
UNinjured_tenx_B3_CATGACATCCCTGACT	CC2	0.480042010545731
injured_tenx_B4_CCGTACTAGAGTACAT	CC2	0.490635007619858
WASP96hr_dropseq_PI24CIRC2_GGACCCGCTGGC.2	CC2	0.626677989959717
injured_tenx_B2_TGGCGCACACCAGGCT	CC2	0.662064015865326
injured_tenx_B2_CTCATTAAGGCCATAG	CC2	0.874152004718781
WASP96hr_dropseq_PI24CIRC2_TGAGCGGTACCA.2	CC2	0.887723982334137
WASP96hr_dropseq_PI24CIRC3_GGTATATTATTT.3	CC2	0.89647901058197
injured_tenx_B2_GCTGCAGCACGGTAAG	CC2	0.919331014156342
WASP96hr_dropseq_PI24CIRC2_CATAGACAATTC.2	CC2	0.985751986503601
WASP96hr_dropseq_PI24CIRC3_ACTCGCCGGTCC.3	CC2	1.01431000232697
WASP96hr_dropseq_PI24CIRC2_TCATGACAGAAT.2	CC2	1.04330003261566
WASP96hr_dropseq_PI24CIRC3_TTTACGCCCCGG.3	CC2	1.05305004119873
WASP96hr_dropseq_PI24CIRC2_CGTGCAGGGGGC.2	CC2	1.1251699924469
WASP96hr_dropseq_PI24CIRC2_AAAGGGCGCTCG.2	CC2	1.14757001399994
WASP96hr_dropseq_PI24CIRC2_GTATGGACATTC.2	CC2	1.18768000602722
WASP96hr_dropseq_PI24CIRC3_TCATATCGGACC.3	CC2	1.21648001670837
WASP96hr_dropseq_PI24CIRC3_AGCAACTGAATT.3	CC2	1.28662002086639
injured_tenx_B4_AGAGCTTGTCAGAATA	CC2	1.29560995101929
WASP96hr_dropseq_PI24CIRC3_AAATCCTTAGGC.3	CC2	1.32042002677917
injured_indrop_PC1_blood2_CTTAATAG.ATCGCGCT.GACAACTT	CC2	1.36340999603271
WASP96hr_dropseq_PI24CIRC2_TACAAGGGGGTT.2	CC2	1.43753004074097
injured_indrop_PC1_blood2_CTTAATAG.GCTACCGT.GAAGGCTT	CC2	1.48852002620697
WASP96hr_dropseq_PI24CIRC3_ACACGCAGGCCA.3	CC2	1.51538002490997
injured_indrop_PC2_blood3_TCGCATAA.CCATCGTC.TTAAGCGT	CC2	1.54051995277405
WASP96hr_dropseq_PI24CIRC3_GCAACATGAGAG.3	CC2	1.58864998817444
WASP96hr_dropseq_PI24CIRC2_TCCTTCACGATG.2	CC2	1.64525997638702
WASP96hr_dropseq_PI24CIRC3_GAGCCCTGGGTC.3	CC2	1.85327994823456

Cell Barcode	Cluster Name	FBgn0046687
injured_tenx_B2_TACTTGTTCACAAACC	CC2	1.88384997844696
WASP96hr_dropseq_PI24CIRC2_GACCCCGTCACG.2	CC2	2.06792998313904
WASP96hr_dropseq_PI24CIRC3_CTTCCTCGATGT.3	PM8	0.571819007396698
WASP96hr_dropseq_PI24CIRC3_TCGCCATCACAT.3	PM8	0.591112971305847
WASP96hr_dropseq_PI24CIRC2_GGCGCGGCATTC.2	PM8	0.770837008953094
WASP96hr_dropseq_PI24CIRC1_TCTTATTCAGGT.1	PM8	0.785090982913971
WASP96hr_dropseq_PI24CIRC3_CTATCCGCTTGA.3	PM8	0.795548975467682
UNinjured_tenx_B3_AGAATAGAGGCATGTG	PM8	0.806922972202301
injured_tenx_B2_GTCGTAATCAGCTCTC	PM8	0.883871972560883
WASP96hr_dropseq_PI24CIRC2_AAGTACCGTCCG.2	PM8	0.892630994319916
WASP96hr_dropseq_PI24CIRC3_GAACATCGAGAT.3	PM8	0.944715023040771
WASP96hr_dropseq_PI24CIRC3_CTAGGTCGGATG.3	PM8	0.996944010257721
WASP96hr_dropseq_PI24CIRC3_ATTGGAGTAAAA.3	PM8	1.00063002109528
WASP96hr_dropseq_PI24CIRC3_ACTTGTGAGGCA.3	PM8	1.00599002838135
injured_tenx_B2_GAATAAGCATGGGAAC	PM8	1.01197004318237
WASP96hr_dropseq_PI24CIRC2_TAACTATGGGTG.2	PM8	1.01296997070312
WASP96hr_dropseq_PI24CIRC2_CCGTAGAGCTGG.2	PM8	1.04472994804382
WASP96hr_dropseq_PI24CIRC3_GCATAGTCCAAG.3	PM8	1.05305004119873
injured_tenx_B4_GCGAGAACACGCTTTC	PM8	1.05438005924225
UNinjured_tenx_B3_TACTCATCAACACGCC	PM8	1.06747996807098
WASP96hr_dropseq_PI24CIRC3_ACAACGAAAGCG.3	PM8	1.10008001327515
WASP96hr_dropseq_PI24CIRC3_ACTTGTTTTCGT.3	PM8	1.10181999206543
WASP96hr_dropseq_PI24CIRC3_TATATCCGTAGA.3	PM8	1.10236001014709
injured_tenx_B4_AAATGCCTCAACACAC	PM8	1.11860001087189
WASP96hr_dropseq_PI24CIRC3_TGTTAAGGAAAT.3	PM8	1.20310997962952
WASP96hr_dropseq_PI24CIRC3_CTGTGGTGGATC.3	PM8	1.22237002849579
WASP96hr_dropseq_PI24CIRC3_GCGATGTGTTCA.3	PM8	1.23071002960205
WASP96hr_dropseq_PI24CIRC3_TGGAATTCATGG.3	PM8	1.25347995758057
UNinjured_indrop_UC1_blood2_TAAGGCTC_AAAGAAAG_GCAACCTG	PM8	1.28910005092621
WASP96hr_dropseq_PI24CIRC3_GAGCCTCTACAC.3	PM8	1.30768001079559
WASP96hr_dropseq_PI24CIRC3_CTAAGGTCTACG.3	PM8	1.31500995159149
UNinjured_tenx_B3_AGTGGGAAGCGTTTAC	PM8	1.36775994300842
injured_tenx_B4_CGGGTCATCTCCTATA	PM8	1.39750003814697
injured_tenx_B4_CTAGAGTAGGATGCGT	PM8	1.39865005016327
UNinjured_tenx_B3_ACTGCTCGTCCAGTAT	PM8	1.56518995761871
injured_tenx_B2_CAGGTGCCAATGAAAC	PM8	1.60561001300812
injured_tenx_B2_TACAGTGGTCTTGTCC	PM8	1.64223003387451
injured_tenx_B4_TACTCATTCGTGGTCG	PM8	1.74032998085022
UNinjured_indrop_UC1_blood2_TAAGGCTC_AAGTCGGG_GTCAATAC	PM8	1.76759004592896
injured_indrop_PC2_blood3_TCGCATAA.ATCCGCAT.AAATGTCG	PM8	1.80183005332947
WASP96hr_dropseq_PI24CIRC3_ACTGGGACGACG.3	PM8	1.82939994335175
WASP96hr_dropseq_PI24CIRC2_GGCGTTCGAGTT.2	PM8	1.8874100446701
injured_tenx_B2_TCGTAGATCAATCACG	PM8	2.00899004936218
WASP96hr_dropseq_PI24CIRC3_TCTACCCCACGC.3	PM8	2.11360001564026
injured_tenx_B2_CGAATGTAGATTACCC	PM8	2.16652989387512
injured_tenx_B2_TGAAAGAGTTCGGCAC	PM8	2.2701199054718

Cell Barcode	Cluster Name	FBgn0046687
UNinjured_indrop_UC1_blood2_TAAGGCTC_TCCCTGGA_CATTTGTT	PM9	1.03892004489899
injured_indrop_PC2_blood3_TCGCATAA.ACGGTTGG.CATACATT	PM9	1.0568300485611
UNinjured_indrop_UC1_blood2_TAAGGCTC_AACGGTAG_TTCCGAGT	PM9	1.09224998950958
UNinjured_indrop_UC1_blood2_TAAGGCTC_CAATTCTC_AGGATTCA	PM9	1.11321997642517
UNinjured_tenx_B3_TATGCCCAGAAACCGC	PM9	1.11679995059967
UNinjured_tenx_B3_AGGCCACTCAAGATCC	PM9	1.17717003822327
injured_indrop_PC1_blood2_CTTAATAG.CCGTAGCT.AAATCGTT	PM9	1.21731996536255
injured_tenx_B2_GACACGCCAGTGAGTG	PM9	1.22508001327515
injured_indrop_PC1_blood2_CTTAATAG.ACGTAAAC.GGAATAGA	PM9	1.27610003948212
UNinjured_indrop_UC1_blood2_TAAGGCTC_TAAGGGCC_TTCGCTGG	PM9	1.28624999523163
injured_indrop_PC2_blood3_TCGCATAA.GGAACGAA.CCGCTGTT	PM9	1.31541001796722
UNinjured_tenx_B1_AAGGAGCTCTCGCTTG	PM9	1.32954001426697
injured_indrop_PC1_blood2_CTTAATAG.CCGATACG.TTCACATA	PM9	1.32974994182587
UNinjured_tenx_B3_GGGCATCGTTACGCGC	PM9	1.33324003219604
injured_tenx_B4_TTCGAAGCAAGTTGTC	PM9	1.37235999107361
injured_indrop_PC2_blood3_TCGCATAA.ATCAATCG.GGTCTGAC	PM9	1.37390005588531
UNinjured_indrop_UC1_blood2_TAAGGCTC_AGCAACGC_TCTAGCAG	PM9	1.39021003246307
injured_indrop_PC2_blood3_TCGCATAA.TCATTTCA.GGCATGCT	PM9	1.40977001190186
injured_tenx_B2_CACACTCGTCTCCACT	PM9	1.42396998405457
injured_indrop_PC1_blood2_CTTAATAG.GGTAAATC.ATTGGGCC	PM9	1.45818996429443
injured_tenx_B4_TGTCCCAAGTCTCGGC	PM9	1.49038004875183
injured_indrop_PC2_blood3_TCGCATAA.ATTACCTT.GCACCTCT	PM9	1.50004994869232
UNinjured_indrop_UC1_blood2_TAAGGCTC_TGCACCAG_GTCGTCGT	PM9	1.5192699432373
injured_indrop_PC1_blood2_CTTAATAG.TCTAAACT.ATTCAGTA	PM9	1.54428005218506
injured_indrop_PC1_blood2_CTTAATAG.GTACGGAC.TTCGGCCT	PM9	1.55774998664856
UNinjured_indrop_UC1_blood2_TAAGGCTC_CACTTGAT_CCGTGTTT	PM9	1.58802998065948
injured_tenx_B2_TACGGTAAGGTACTCT	PM9	1.58802998065948
injured_indrop_PC2_blood3_TCGCATAA.CCTACGCT.TTCGTTCC	PM9	1.59613001346588
injured_tenx_B4_TATTACCTCATCTGCC	PM9	1.65992999076843
UNinjured_indrop_UC1_blood2_TAAGGCTC_TCCTATAT_GAATACGC	PM9	1.66061997413635
injured_indrop_PC2_blood3_TCGCATAA.GTGATAAA.GCAAAGCC	PM9	1.67386996746063
injured_tenx_B2_CCACCTAAGCCACGCT	PM9	1.83957004547119
injured_tenx_B2_AGAATAGCATCACGAT	PM9	1.86817002296448
injured_indrop_PC2_blood3_TCGCATAA.ATGCGGAG.CAGACCAT	PM9	1.98884999752045
injured_indrop_PC1_blood2_CTTAATAG.CCTATTTA.TGGTTTCT	PM9	2.00148010253906
